# Design, Synthesis and Biological Evaluation of 1-Phenyl-2-(phenylamino) Ethanone Derivatives as Novel MCR-1 Inhibitors

**DOI:** 10.3390/molecules24152719

**Published:** 2019-07-26

**Authors:** Xiu-juan Lan, Hai-tao Yan, Feng Lin, Shi Hou, Chen-chen Li, Guang-shu Wang, Wei Sun, Jun-hai Xiao, Song Li

**Affiliations:** 1 School of Pharmaceutical Sciences, Jilin University, Changchun 130021, China; 2 National Engineering Research Center for Strategic Drugs, Beijing Institute of Pharmacology and Toxicology, Beijing 100850, China; 3 State Key Laboratory of Toxicology and Medical Countermeasures, Beijing Institute of Pharmacology and Toxicology, Beijing 100850, China; 4 School of Life Sciences, Jilin University, Changchun 130021, China

**Keywords:** Colistin resistance, virtual screening, MCR-1 inhibitors, 1-phenyl-2-(phenylamino) ethanone derivatives

## Abstract

Polymyxins are considered to be the last-line antibiotics that are used to treat infections caused by multidrug-resistant (MDR) gram-negative bacteria; however, the plasmid-mediated transferable colistin resistance gene (*mcr-1*) has rendered polymyxins ineffective. Therefore, the protein encoded by *mcr-1*, MCR-1, could be a target for structure-based design of inhibitors to tackle polymyxins resistance. Here, we identified racemic compound **3** as a potential MCR-1 inhibitor by virtual screening, and 26 compound **3** derivatives were synthesized and evaluated in vitro. In the cell-based assay, compound **6g**, **6h**, **6i**, **6n**, **6p**, **6q**, and **6r** displayed more potent activity than compound **3**. Notably, 25 μΜ of compound **6p** or **6q** combined with 2 μg·mL-1 colistin could completely inhibit the growth of BL21(DE3) expressing *mcr-1*, which exhibited the most potent activity. In the enzymatic assay, we elucidate that **6p** and **6q** could target the MCR-1 to inhibit the activity of the protein. Additionally, a molecular docking study showed that **6p** and **6q** could interact with Glu246 and Thr285 via hydrogen bonds and occupy well the cavity of the MCR-1 protein. These results may provide a potential avenue to overcome colistin resistance, and provide some valuable information for further investigation on MCR-1 inhibitors.

## 1. Introduction

Nowadays, antibiotic resistance in gram-negative pathogens is recognized as one of the most serious global threats to public health [1]. The increase of multidrug-resistant (MDR) organisms results in more than 700,000 deaths worldwide each year and poses a heavy burden on healthcare costs [2]. The lack of effective antibiotics to treat infections caused by MDR bacteria is pushing us to the edge of a postantibiotic era. To address this situation, polymyxins, which are old antibiotics, have been reused in clinics to combat the infections caused by MDR pathogens [3], and they are now used as one of the “last line” treatments for MDR pathogens [4].

Polymyxins are cationic antimicrobial peptides (CAMP) that were isolated from *Paenibacillus polymyxa* in 1947. There are five types of polymyxins: A, B, C, D and E, and among these, polymyxin B and polymyxin E (colistin) are used in clinical therapies [3,5]. In the past years, the polymyxin resistance mechanisms are mediated chromosomally, such as the two-component regulatory systems *PhoPQ* and *PmrAB*, as well as the inactivation of *mgrB*, leading to the modification of lipid A with phosphoethanolamine (PEA) or 4-amino-4-arabinose [6,7,8]. Recently, in late 2015, the first plasmid-mediated colistin resistance gene, *mcr-1*, was reported in China. The *mcr-1* gene encodes PEA transferase, leading to the addition of the PEA moiety, which is from the phosphatidylethanolamine (PE) donor substrate, to lipid A, decreasing the negative charges of lipid A and impacting the interactions between colistin and the membrane of pathogens, that is, colistin resistance has occurred [9].

The emergence of *mcr-1* is likely to bring on the rapid dissemination of polymyxin resistance, due to the horizontal transfer of plasmids in various bacteria strains. To date, *mcr-1* has been found in food, animal, and human samples [10]. Additionally, it is worrisome that a growing number of *Enterobacteriaceae* strains with *mcr-1* coexisting with carbapenem resistance genes, such as *NDM-5* and *NDM-9*, have been identified [11,12,13]. *Mcr-1* has been isolated from more than 50 countries distributed on five continents, and there is an increasing risk that pan-drug-resistant bacteria will emerge globally.

**Figure 1:**
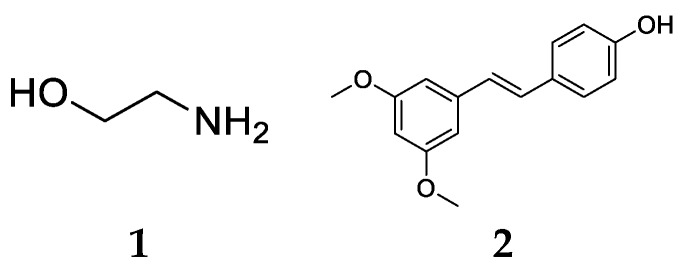
Structures of (**1**) ethanolamine (ETA), and (**2**) pterostilbene.

Currently, there are few research projects on MCR-1 inhibitors have been reported, such as those generating ethanolamine (ETA) [14] and pterostilbene [15,16], with the structures shown in Figure 1. The combination of 10 mM ETA with 4 μg·mL-1 polymyxin B inhibits the growth of *Escherichia coli* strain Rosetta cells expressing *mcr-1*; pterostilbene could restore susceptibility to colistin against *mcr-1*-positive *Escherichia coli* strains, and *mcr-1*-positive *K. pneumoniae* isolates when it was respectively 32 μg·mL-1 and 16 μg·mL-1. Both of them displayed low activity, and the mechanism remains to be further clarified. Thus, the development of novel agents, to combat colistin-resistance, was warranted. We herein report that compound **3** was selected as a potential MCR-1 inhibitor by virtual screening for the first time, and 26 compound **3** derivatives were designed, synthesized, and evaluated in vitro, and an enzymatic assay was performed to elucidate that active compounds could target the MCR-1 protein. Additionally, structure-activity relationship (SAR) studies and molecular docking analysis were performed to determine critical structure responsible for their MCR-1 inhibitory activity. The results might provide some information to address the threats caused by *mcr-1*.

## 2. Results and Discussion

### 2.1. Finding Potential MCR-1 Inhibitors by Virtual Screening

The full-length MCR-1 protein is composed of an N-terminal transmembrane domain and a periplasmic C-terminal catalytic domain. However, only the crystal structure of the C-terminal catalytic domain was reported [17,18,19,20,21]. The active site of the MCR-1 catalytic domain is considered to be the region of one zinc surrounded by E246, T285, D465, and H466, and T285 was believed to act as the acceptor for the PEA group during the transfer reaction. However, the current data makes the role of zinc in the PEA transfer reaction uncertain. In view of the research on the MCR-1 catalytic domain, we screened over 8000 organic compounds in our in-house compound library based on the MCR-1 crystal structure (PDB: 5GRR) [19]. The molecules were ranked according to their docking scores, and the molecules which have shape complementarity and form more hydrogen bonds with the active site of MCR-1 were selected. Ultimately, a total of 30 compounds were selected as potential MCR-1 inhibitors.

### 2.2. Evaluation of the Activity of Potential MCR-1 Inhibitors

**Figure 2:**
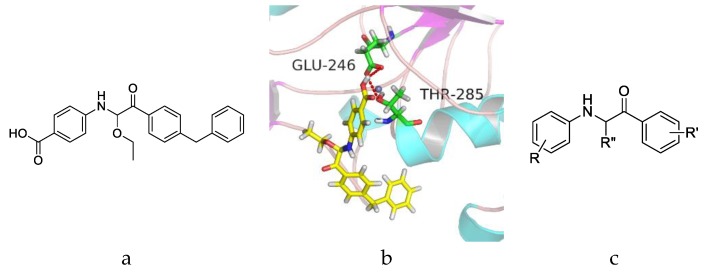
(**a**) Structure of racemic compound **3**. (**b**) Docking pattern of the interactions between compound **3** and MCR-1 catalytic domain with H-bonds shown by red lines. (**c**) The core structure of 1-phenyl-2-(phenylamino) ethanone.

We here evaluated the activities of the above 30 compounds by applying the broth microdilution method [22], and the strain of *E. coli* BL21(DE3) was used to perform the cell-inhibitory assay. In this assay, BL21(DE3) and BL21(DE3) transformed with only the null pET-28a(+) plasmid were used as a negative control (NC), and BL21(DE3) transformed with the pET-28a(+) plasmid carrying the full-length *mcr-1* gene was used as the wild type (WT) control. The Clinical and Laboratory Standards Institute (CLSI) defines colistin resistance as ≥8 μg·mL^−1^ and colistin susceptibility as ≤2 μg·mL^−1^. The MICs of colistin against BL21(DE3), BL21(DE3)+pET-28a(+), and BL21(DE3)+pET-28a(+)+*mcr-1* are shown in Table 1. As can be seen, the MICs of colistin against the negative control bacterias are 2 μg·mL^−1^, and the MIC of colistin against BL21(DE3) transformed with the *mcr-1* gene is 8 μg·mL^−1^, the results indicated that BL21(DE3) expressing *mcr-1* confers colistin resistance. Therefore, the MIC assay using the BL21(DE3)+pET-28a(+)+*mcr-1* was performed to screen the efficacies of the 30 compounds. First, we tested whether the compounds alone could influence the growth of the above strain at a concentration of 100 μΜ. The wells of the microwell plates were turbid, and the results showed that none of the compounds influenced the growth of the strain. Then, we tested which compound could decrease the colistin resistance of 8 μg·mL^−1^ to colistin susceptibility of 2 μg·mL^−1^. The concentrations of the compounds used were 50 μΜ and 100 μΜ. Among the 30 screened compounds, 100 μΜ compound **3** combined with 2 μg·mL^−1^ colistin could completely inhibit the growth of BL21(DE3)+pET-28a(+)+*mcr-1*, and all of the results suggested that compound **3** may inhibit the activity of MCR-1 and restore the susceptibility of colistin. Therefore, compound **3** is identified as a potential MCR-1 inhibitor. The structure of compound **3** is shown in Figure 2a.

### 2.3. Compound Design

The active site of 5GRR occupies a shallow depression on a relatively flat face of the protein. However, the zinc was so far away from the shallow depression that molecules could not coordinate with zinc, while molecules could interact with key amino acids. The docking results between MCR-1 catalytic domain and compound **3** are presented in Figure 2b. The results showed that compound **3** occupies well the shallow depression of MCR-1 catalytic domain, and the carboxyl group interacted with Glu246 and Thr285 by hydrogen bonding (H-bond). The above observations indicated that 1-phenyl-2-(phenylamino) ethanone which mainly contributed to occupying the cavity and the carboxyl group which forms H-bonds with essential amino acids might be important for the activity against MCR-1, therefore, the structure of 1-phenyl-2-(phenylamino) ethanone was chosen as the core structure (Figure 2c), and the effects of the carboxyl group (R), R′ and R′′ substituents were investigated on SARs.

Recently, the modelled structure of the full-length MCR-1 through structural modelling with the *Neisseria meningitidis* EptA [23] (*Nm*EptA, PDB: 5FGN) as a template was reported, and the study suggested that the trans-membrane domain was essential for MCR-1 activity and there is a cavity for PE substrate entry into MCR-1. The cavity which is composed of a catalytic site and a PE lipid substrate-binding site was considered to be the active site of MCR-1 [24]. Based on the above study, we attempted to synthesize a series of compounds with a linear alkyl chain at R′, which were similar to the fatty chain of PE substrate, and investigated its effect on activity.

### 2.4. Chemistry

**Scheme 1:**
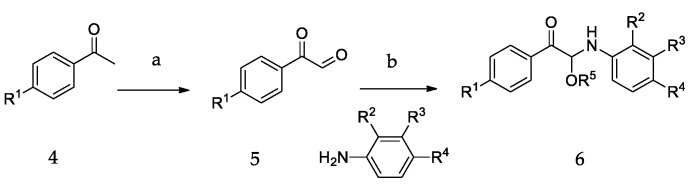
The synthetic routes of **6a**–**6r**. Reagents and conditions: (**a**) SeO_2_, 1,4-dioxane, H_2_O, reflux; (**b**) ROH (CH_3_OH, EtOH, (CH_3_)_2_CHOH), r.t.

**Scheme 2:**
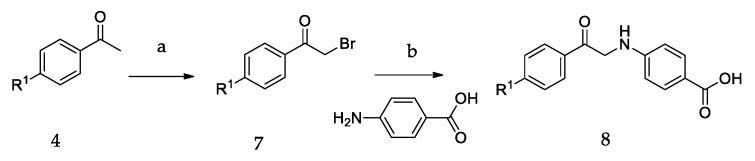
The synthetic routes of **8a**–**8d**. Reagents and conditions: (**a**) CuBr_2_, EtOAc, reflux; (**b**) EtOH, r.t.

**Scheme 3:**
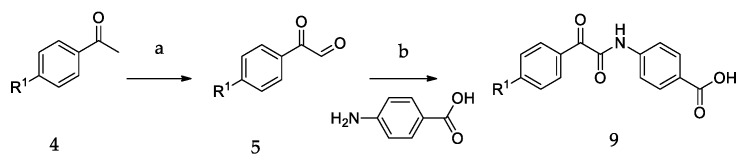
The synthetic routes of **9a**–**9d**. Reagents and conditions: (**a**) SeO_2_, 1,4-dioxane, H_2_O, reflux; (**b**) SeO_2_, Py, MeCN, reflux.

The synthetic routes of all of the new compounds in this study are shown in Scheme 1, Scheme 2 and Scheme 3. In Scheme 1, commercially available acetophenone with various substituent groups **4** was oxidized by selenium dioxide, yielding corresponding phenylglyoxals **5**, and then, **5** was reacted with primary amines to yield **6a**–**6r**. In Scheme 2, the synthesis of **8a**–**8d** also started from various acetophenones **4**, by adding copper (II) bromide, and the bromation was accomplished to give **7**. Then, 4-aminobenzoic acid was added to an ethanolic solution of **7**, yielding **8a**–**8d**. In Scheme 3, the acetophenones with various substituent groups **4** were oxidized by selenium dioxide, yielding the corresponding phenylglyoxals **5**. Then, 4-aminobenzoic acid reacted with **5** in the presence of selenium dioxide and pyridine, an oxidation reaction occurs, and **9a**–**9d** were obtained.

### 2.5. Activity Evaluation and SAR Study

As mentioned in Section 2.2, first, we tested whether the synthesized compounds alone could influence the growth of the strain, the wells of the microwell plates were turbid when the concentration of the compounds used was 100 μΜ, and the results showed that none of the novel compounds influenced the growth of the strain. After that, the activities of the compounds in combination with 2 μg·mL^−1^ colistin were tested. The concentrations of the compounds used were 12.5 μΜ, 25 μΜ, 50 μΜ, and 100 μΜ. The results of the activity evaluation of the synthesized compounds are listed in Table 2 and Table 3. As can be seen, seven compounds (**6g**, **6h**, **6i**, **6n**, **6p**, **6q**, and **6r**) displayed more potent activity than compound **3**. Among them, 25 μΜ of compound **6p** and **6q** in combination with 2 μg·mL^−1^ colistin could completely inhibit the growth of BL21(DE3) expressing *mcr-1*, which exhibited the most potent activity.

For the first series of compounds **6a**–**6i**, shortening the length of the R^1^ substituent was investigated, and the benzyl was replaced by smaller fluorine, hydroxyl, piperidine, and cyclohexyl groups; meanwhile, the R^4^ position was substituted with carboxyl, ester, or nitro group. As shown in Table 2, the introduction of fluorine, hydroxyl, or piperidine at R^1^ (**6a–6d**) led to a complete loss of activity. As for the cyclohexyl substituent (**6e–6i**), the esterification or the introduction of nitro group at R^4^ resulted in the loss of activity, and retaining the carboxyl of the R^4^ group (**6g**, **6h**, and **6i**) could increase the MCR-1 inhibitory activity.

Then, the compounds **6j–6r**, which switched the benzyl group of R^1^ to a linear alkane chain, were synthesized based on the study of the modelled structure of the full-length MCR-1 [24]. As shown in Table 2, compounds **6j–6l**, which introduced n-propyl at R^1^, lost potency for MCR-1. When R^1^ was substituted by n-hexyl or n-octyl, the compounds (**6m–6r**) which keep the carboxyl group at R^4^ exhibited MCR-1 inhibitory activity, especially **6p** and **6q**, which displayed the most potent activity. Surprisingly, compared to **6n**, the introduction of methyl at R^2^ (**6p**) resulted in a significant increase in the inhibitory activity. However, the introduction of a methyl group at R^3^ (**6o** and **6r**) led to a decreased activity. Additionally, from **6j**, **6n**, and **6q**, we found that the extension of carbon chains is more conducive to MCR-1 inhibitory activity with n-octyl group > n-hexyl > n-propyl. Therefore, the SARs existing in these compounds listed in Table 2 indicated that the R^1^ and R^4^ substituents play important roles in the MCR-1 inhibitory activity, and the compounds in which R^1^ was substituted by alaphatic group (with a cyclohexyl, n-hexyl or n-octyl group) and the carboxyl was kept constant at R^4^ displayed inhibitory activity against MCR-1. Additionally, the length of the chain substitution had a significant influence on the MCR-1 inhibitory activity, the longer the substitution was, the better the activity. Moreover, the activity of **6o** or **6r** substituted with –CH_3_ at R^3^ was lower than **6p** substituted with –CH_3_ at R^2^.

Having produced optimized substituents at positions R^1^ and R^4^, we turned our attention to the R^5^ substituent. Based on the compounds **6g**, **6h**, and **6i**, which displayed the same activities with the only difference in R^5^, it was reasonable to assume that the removal of the R^5^ substituent will still result in the maintenance of some activity. Thus, the compounds **8a–8d** and **9a–9d** that removed the alkoxy substituents were synthesized. The results listed in Table 3 showed that the removal of the R^5^ substituent resulted in activity losses, and the results indicated that the R^5^ substituents are also essential to the MCR-1 inhibitory activity.

Taking into account the above SAR studies, we could conclude that the combination of R^1^, R^4^, and R^5^ substituents is very important for MCR-1 inhibitory activity, and the SARs of these compound **3** derivatives was that compounds substituted with a lipophilic R^1^ (cyclohexyl, n-hexyl or n-octyl group) and the maintenance of the carboxyl and EtO at R^4^ and R^5^ displayed better MCR-1 inhibitory activity. Additionally, the activity of the compound substituted with –CH_3_ at R^2^ was superior to that of the compound substituted with –CH_3_ at R^3^.

### 2.6. Inhibitory Effects Against MCR-1

**Figure 3:**
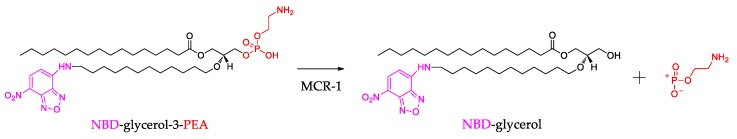
The scheme for the enzymatic reaction catalyzed by the MCR-1 protein.

In order to elucidate that the synthesized compounds could inhibit the activity of MCR-1 protein, we performed an enzymatic assay to evaluate whether synthesized compounds could target the MCR-1. In this assay, the MCR-1 protein was purified as Xu and coauthors described [24], and the PEA donor substrate with a fluorescent label (NBD-glycerol-3-PEA) was used [23]. As shown in Figure 3, the MCR-1 protein could remove the PEA moiety from the substrate NBD-glycerol-3-PEA and give a fluorescent product NBD-glycerol. The substrate NBD-glycerol-3-PEA and the product NBD-glycerol could exhibit different Rf value by thin layer chromatography (TLC) using blue light (455–485 nm), therefore, the TLC was used to separate the mixture in the enzymatic reaction [23]. If the synthesized compounds could inhibit the reaction of PEA transfer catalyzed by MCR-1, and the TLC will mainly show the substrate NBD-glycerol-3-PEA band. Thus, this system might be used to test the inhibitory effect against MCR-1 protein.

**Figure 4:**
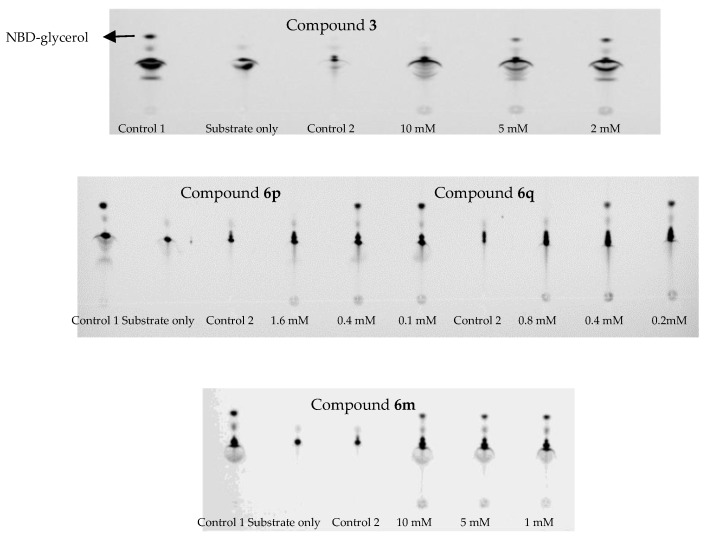
The bands of compounds **3**, **6p**, **6q**, and **6m** interacted with MCR-1 protein for inhibiting the reaction of phosphoethanolamine (PEA) transfer by thin layer chromatography (TLC); Control 1: The bands of substrate interacted with MCR-1 protein; Control 2: The bands of tested compounds interacted with the substrate.

In this assay, 0.2 mM substrate NBD-glycerol-3-PE was used, and the results are shown in Figure 4, the substrate NBD-glycerol-3-PEA was the lower spot, and the control 1 showed that the upper spot was the product NBD-glycerol [24]. The control 2 showed that compound **3**, **6p**, **6q** or **6m** (**6m** was one of the inactive compounds) could not influence the spot of the substrate. Therefore, this system was used to test whether these selected compounds could target the MCR-1 protein. For compound **3**, the TLC exhibited one spot of the substrate when it was 10 mM, and the results indicated that compound **3** could inhibit the reaction of PEA transfer catalyzed by MCR-1. For **6p** and **6q**, the TLC exhibited one spot of the substrate when they were respectively 1.6 mM and 0.8 mM, and the results showed that compound **6p** and **6q** also could inhibit the reaction of PEA transfer catalyzed by MCR-1, and compound **6q** showed more potent inhibition than **6p**. Obviously, both of them exhibited more potent inhibition against MCR-1 protein than compound **3**. However, compound **6m** still clearly exhibited two spots of the substrate and the product when it was 10 mM, and the results indicated that **6m** could not inhibit the reaction of PEA transfer catalyzed by MCR-1, which was consistent with the loss of inhibitory activity in the cell-based assay. The above results suggested that the active compounds **6p** and **6q** could interact with MCR-1 and inhibit the activity of the protein.

### 2.7. Molecular Docking

**Figure 5:**
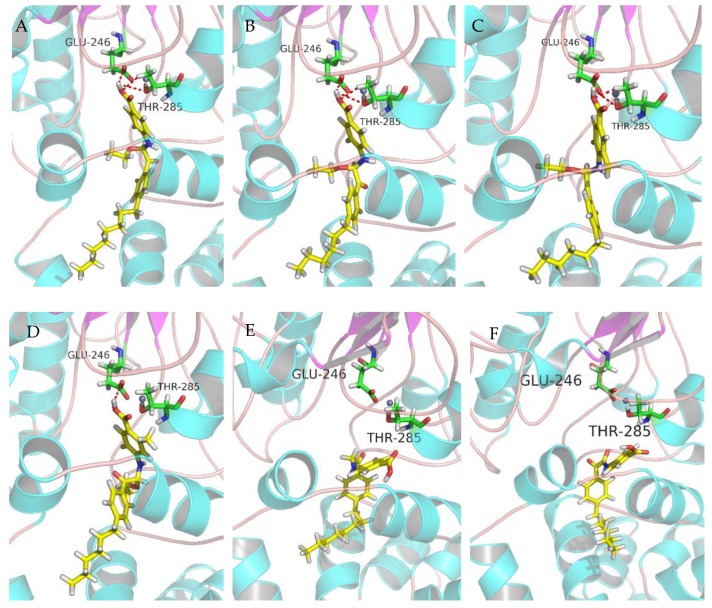
Docking patterns of the interaction between compounds **A**: **6q**; **B**: **6n**; **C**: **6p**; **D**: **6o**; **E**: **8d**; **F**: **9d** and the full-length MCR-1 protein with H-bonds shown by red lines. (**A**)–(**F**) is that docking pattern of the interaction between every selected compound and the full-length MCR-1 protein with H-bonds.

After the previous test of biological activity, in order to further obtain the relationship between the structure of the active compounds and the inhibition of MCR-1 protein, several compounds were molecularly docked with the full-length MCR-1 modelled by Swiss-Model. The molecular docking simulation results are presented in Figure 5. Since the studies have suggested that Thr285 was believed to act as the acceptor for the PEA group during the transfer reaction, therefore, the interaction with Thr285 is crucial to MCR-1 inhibitory activity. As shown in Figure 5a–c, the carboxyl group of the active compound **6q**, **6n** or **6p** bonds to Glu246 and Thr285 via hydrogen bonds, and the fatty chain of compounds occupies well the cavity of MCR-1 protein. Comparing the docking result of **6p**, compound **6o** lost the hydrogen bond interaction with the Thr285 (Figure 5d), therefore, the decrease in the anti-MCR-1 activity of **6o** might be due to the loss of the H-bond with Thr285. Additionally, molecular docking was also performed to study the binding mode of compound **8d** and **9d** (the compounds of series 2 and series 3), and the docking results are shown in Figure 5e,f. As can be seen, both of them lost the H-bonds with Glu246 and Thr285, which played an important role in anti-MCR-1 activity. This result might explain why **8d** and **9d** lost their activity against MCR-1 protein, and the results also elucidate the SARs that the R^5^ substituents are also essential to the MCR-1 inhibitory activity.

Additionally, another configuration of these compounds was also molecularly docked with the full-length MCR-1. The docking results showed that the two configurations of the molecules did not display an obvious difference in the docking modes, and the cavity of MCR-1 have a good match with the two configurations. Therefore, on the basis of the docking results, the configuration of compounds may not contribute to the MCR-1 inhibitory activity. The docking modes of another configuration are provided in the Supplementary Data.

## 3. Materials and Methods

### 3.1. Structure-Based Virtual Screening

The MCR-1 crystal structure (PDB: 5GRR) was downloaded from the Protein Data Bank (PDB), and the molecular docking was carried out using Gold (The Cambridge Crystallographic Data Centre). Before docking calculations, the Zn601 which surrounded by E246, T285, D465, and H466 was kept, the other zinc and the water molecules of the crystal structure were deleted, and hydrogen atoms were added. The structures of 8141 compounds in our in-house compound library were transferred from two-dimensional to three-dimensional by concord4.0 (Tripos, USA) with energy minimization automatically for the next docking simulations. During docking calculations, zinc was selected as the active site, and the cavity was set to 10 Å; template was set to chemscore_kinase; number of GA Runs were set to 30; scoring function was set to GoldScore; GA search options was set to slow. After the docking process was finished, the docking scores were in order from high scores to low scores, firstly, the compounds which scored more than 50 were selected, and 286 compounds were obtained, and then, the molecules which have shape complementarity and form more hydrogen bonds with the active site of MCR-1 were used for the analysis of docking and the interactions.

### 3.2. Chemistry

Solvents and reagents used in the synthesis were obtained from Beijing Innochem Science and Technology Co. Ltd. (Beijing, China) and J&K Scientific Ltd. (Beijing, China). Chemical reactions were monitored by thin layer chromatography (TLC). The structures of the products were identified by ^1^H and ^13^C-NMR spectroscopy (JNM-ECA-400, Japan), with DMSO-*d*_6_ used as the solvent. The molecular weights of the products were measured by high resolution mass spectrometry (HRMS) with electrospray ionization (ESI) as the ionization mode (Agilent 1260-G6230A, Germany). The synthesis of the compounds, NMR and mass spectra are provided in the Supplementary Materials.

Methyl 4-((1-Ethoxy-2-(4-fluorophenyl)-2-oxoethyl)amino)benzoate (**6a**) ^1^H-NMR (400 MHz, DMSO-*d*_6_) δ 8.17 (dd, *J* = 8.9, 5.6 Hz, 2H), 7.76 (d, *J* = 8.8 Hz, 2H), 7.46–7.38 (m, 3H), 7.02 (d, *J* = 8.9 Hz, 2H), 6.29 (d, *J* = 8.6 Hz, 1H), 3.77 (s, 3H), 3.56–3.49 (m, 1H), 3.44–3.37 (m, 1H), 1.03 (t, *J* = 7.0 Hz, 3H). ^13^C-NMR (101 MHz, DMSO-*d*_6_) δ 192.94, 166.73, 150.90, 132.63, 131.32, 118.97, 116.45, 116.23, 113.50, 81.86, 60.81, 51.96, 15.76. HRMS(ESI) (*m/z*): Calad. for C_18_H_18_FNO_4_ 331.3382; found 286.0875 [M − C_2_H_5_O]^+^.

Methyl 4-((1-Ethoxy-2-(4-hydroxyphenyl)-2-oxoethyl)amino)benzoate (**6b**) ^1^H-NMR (400 MHz, DMSO-*d*_6_) δ 10.55 (s, 1H), 8.00 (d, *J* = 8.8 Hz, 2H), 7.76 (d, *J* = 8.8 Hz, 2H), 7.35 (d, *J* = 8.4 Hz, 1H), 7.02 (d, *J* = 8.9 Hz, 2H), 6.88 (d, *J* = 8.8 Hz, 2H), 6.9 (d, *J* = 8.4 Hz, 1H), 3.77 (s, 3H), 3.53–3.45 (m, 1H), 3.41–3.33 (m, 1H), 1.02 (t, *J* = 7.0 Hz, 3H). ^13^C-NMR (101 MHz, DMSO-*d*_6_) δ 192.15, 166.76, 163.29, 151.00, 132.33, 131.30, 126.10, 118.77, 115.87, 113.47, 81.29, 60.38, 51.95, 15.80. HRMS(ESI) (*m/z*): Calad. for C_18_H_19_NO_5_ 329.3472; found 284.0919 [M-C_2_H_5_O]^+^.

4-((1-Ethoxy-2-oxo-2-(4-(piperidin-1-yl)phenyl)ethyl)amino)-2-methylbenzoic Acid (**6c**) ^1^H-NMR (400 MHz, DMSO-*d*_6_) δ 12.05 (s, 1H), 7.93 (d, *J* = 9.1 Hz, 2H), 7.72 (d, *J* = 9.3 Hz, 1H), 7.05 (d, *J* = 8.5 Hz, 1H), 6.96 (d, *J* = 9.1 Hz, 2H), 6.82 (d, *J* = 6.7 Hz, 2H), 6.13 (d, *J* = 8.5 Hz, 1H), 3.51–3.44 (m, 1H), 3.41 (brs, 4H), 3.37–3.32 (m, 1H), 2.47 (s, 3H), 1.58 (brs, 6H), 1.02 (t, *J* = 7.0 Hz, 3H). ^13^C-NMR (101 MHz, DMSO-*d*_6_) δ 191.21, 168.73, 154.60, 149.81, 142.15, 133.15, 131.84,122.84, 118.86, 116.33, 113.14, 111.02, 81.01, 60.09, 48.08, 25.40, 24.54, 22.89, 15.87. HRMS(ESI) (*m/z*): Calad. for C_23_H_28_N_2_O_4_ 396.4794; found 351.1708 [M-C_2_H_5_O]^+^.

2-Ethoxy-2-((4-nitrophenyl)amino-1-(4-(piperidin-1-yl)phenyl)ethanone (**6d**) ^1^H-NMR (400 MHz, DMSO-*d*_6_) δ 8.06 (d, *J* = 8.9 Hz, 2H), 7.95 (d, *J* = 8.6 Hz, 2H), 7.88 (d, *J* = 7.9 Hz, 1H), 7.10 (d, *J* = 8.9 Hz, 2H), 6.97 (d, *J* = 8.2 Hz, 2H), 6.22 (d, *J* = 7.8 Hz, 1H), 3.52–3.48 (m, 1H), 3.42 (brs, 4H), 3.40–3.32 (m, 1H), 1.58 (brs, 6H), 1.03 (t, *J* = 6.9 Hz, 3H). ^13^C-NMR (101 MHz, DMSO-*d*_6_) δ 190.68, 154.68, 153.13, 138.19, 131.94, 126.30, 122.58, 113.48, 113.11, 80.94, 60.45, 48.05, 25.42, 24.53, 15.79. HRMS(ESI) (*m/z*): Calad. for C_21_H_25_N_3_O_4_ 383.4409; found 384.1918 [M + H]^+^.

Methyl 4-((2-(4-CYCLOHEXYLPHENYl)-1-ethoxy-2-oxoethyl)amino)benzoate (**6e**) ^1^H-NMR (400 MHz, DMSO-*d*_6_) δ 8.02 (d, *J* = 8.3 Hz, 2H), 7.77 (d, *J* = 8.8 Hz, 2H), 7.44 (d, *J* = 8.6 Hz, 1H), 7.40 (d, *J* = 8.4 Hz, 2H), 7.02 (d, *J* = 8.8 Hz, 2H), 6.26 (d, *J* = 8.6 Hz, 1H), 3.77 (s, 3H), 3.56–3.48 (m, 1H), 3.45–3.37 (m, 1H), 2.62–2.55 (m, 1H), 1.80–1.69 (m, 5H), 1.47–1.22 (m, 5H), 1.04 (t, *J* = 7.0 Hz, 3H). ^13^C-NMR (101 MHz, DMSO-*d*_6_) δ 193.63, 166.74, 154.50, 150.97, 132.61, 131.32, 129.81, 127.59, 118.87, 113.46, 81.68, 60.73, 51.95, 44.41, 33.98, 26.71, 26.00, 15.80. HRMS(ESI) (*m/z*): Calad. for C_24_H_29_NO_4_ 395.4914; found 350.1751 [M − C_2_H_5_O]^+^.

1-(4-Cyclohexylphenyl)-2-ethoxy-2-((4-nitrophenyl)amino)ethanone (**6f**) ^1^H-NMR (400 MHz, DMSO-*d*_6_) δ 8.08 (d, *J* = 9.2 Hz, 2H), 8.04–8.01 (m, 3H), 7.42 (d, *J* = 8.3 Hz, 2H), 7.10 (d, *J* = 9.3 Hz, 2H), 6.35 (d, *J* = 8.3 Hz, 1H), 3.59–3.51 (m, 1H), 3.46–3.39 (m, 1H), 2.63–2.57 (m, 1H), 1.81–1.69 (m, 5H), 1.48–1.23 (m, 5H), 1.05 (t, *J* = 7.0 Hz, 3H). ^13^C-NMR (101 MHz, DMSO-*d*_6_) δ 193.27, 154.66, 153.02, 138.36, 132.45, 129.88, 127.62, 126.32, 113.49, 81.55, 61.05, 44.42, 33.99, 26.70, 26.00, 15.75. HRMS(ESI) (*m/z*): Calad. for C_22_H_26_N_2_O_4_ 382.4528; found 337.1546 [M − C_2_H_5_O]^+^.

4-((2-(4-Cyclohexylphenyl)-1-ethoxy-2-oxoethyl)amino)benzoic Acid (**6g**) ^1^H-NMR (400 MHz, DMSO-*d*_6_) δ 12.26 (s, 1H), 8.01 (d, *J* = 8.4 Hz, 2H), 7.73 (d, *J* = 8.8 Hz, 2H), 7.41 (d, *J* = 8.4 Hz, 2H), 7.35 (d, *J* = 8.7 Hz, 1H), 6.99 (d, *J* = 8.9 Hz, 2H), 6.24 (d, *J* = 8.6 Hz, 1H), 3.55–3.48 (m, 1H), 3.45–3.37 (m, 1H), 2.62–2.57 (m, 1H), 1.80–1.69 (m, 5H), 1.48–1.22 (m, 5H), 1.04 (t, *J* = 7.0 Hz, 3H). ^13^C-NMR (101 MHz, DMSO-*d*_6_) δ 193.68, 167.88, 154.48, 150.62, 132.63, 131.47, 129.79, 127.60, 120.01, 113.31, 81.73, 60.73, 44.40, 33.99, 26.71, 26.00, 15.82. HRMS(ESI) (*m/z*): Calad. for C_23_H_27_NO_4_ 381.4648; found 336.1594 [M − C_2_H_5_O]^+^.

4-((2-(4-Cyclohexylphenyl)-1-methoxy-2-oxoethyl)amino)benzoic Acid (**6h**) ^1^H-NMR (400 MHz, DMSO-*d*_6_) δ 12.34 (s, 1H), 8.02 (d, *J* = 8.3 Hz, 2H), 7.76 (d, *J* = 8.7 Hz, 2H), 7.40 (d, *J* = 8.3 Hz, 2H), 7.37 (d, *J* = 8.6 Hz, 1H), 7.02 (d, *J* = 8.8 Hz, 2H), 6.25 (d, *J* = 8.5 Hz, 1H), 3.17 (s, 3H), 2.62–2.56 (m 1H), 1.80–1.69 (m, 5H), 1.47–1.22 (m, 5H). ^13^C-NMR (101 MHz, DMSO-*d*_6_) δ 193.46, 167.88, 154.57, 150.47, 132.60, 131.46, 129.82, 127.62, 120.18, 113.44, 82.22, 52.33, 44.42, 33.99, 26.71, 26.00. HRMS(ESI) (*m/z*): Calad. for C_22_H_25_NO_4_ 367.4382; found 336.1594 [M − CH_3_O]^+^.

4-((2-(4-Cyclohexylphenyl)-1-isopropoxy-2-oxoethyl)amino)benzoic Acid (**6i**) ^1^H-NMR (400 MHz, DMSO-*d*_6_) δ 12.26 (s, 1H), 8.01 (d, *J* = 8.4 Hz, 2H), 7.73 (d, *J* = 8.8 Hz, 2H), 7.40 (d, *J* = 8.3 Hz, 2H), 7.34 (d, *J* = 8.8 Hz, 1H), 6.96 (d, *J* = 8.9 Hz, 2H), 6.22 (d, *J* = 8.7 Hz, 1H), 3.87–3.81 (m, 1H), 2.61–2.56 (m, 1H), 1.80–1.69 (m, 5H), 1.47–1.22 (m, 5H), 1.06 (d, *J* = 6.1 Hz, 3H), 1.03 (d, *J* = 6.1 Hz, 3H). ^13^C-NMR (101 MHz, DMSO-*d*_6_) δ 194.13, 167.87, 154.33, 150.67, 132.75, 131.46, 129.80, 127.54, 119.90, 113.27, 80.89, 67.92, 44.38, 33.99, 26.71, 26.00, 23.43, 23.29. HRMS(ESI) (*m/z*): Calad. for C_24_H_29_NO_4_ 395.4914; found 336.1594 [M − C_3_H_7_O]^+^.

4-((1-Ethoxy-2-oxo-2-(4-propylphenyl)ethyl)amino)benzoic Acid (**6j**) ^1^H-NMR (400 MHz, DMSO-*d*_6_) δ 12.26 (s, 1H), 8.01 (d, *J* = 8.3 Hz, 2H), 7.74 (d, *J* = 8.8 Hz, 2H), 7.38 (d, *J* = 8.4 Hz, 2H), 7.35 (d, *J* = 8.8 Hz, 1H), 7.00 (d, *J* = 8.8 Hz, 2H), 6.25 (d, *J* = 8.6 Hz, 1H), 3.56–3.48 (m, 1H), 3.45–3.37 (m, 1H), 2.63 (t, *J* = 7.2 Hz, 2H), 1.66–1.57 (m, 2H), 1.04 (t, *J* = 7.0 Hz, 3H), 0.90 (t, *J* = 7.3 Hz, 3H). ^13^C-NMR (101 MHz, DMSO-*d*_6_) δ 193.76, 167.89, 150.63, 149.38, 132.52, 131.47, 129.68, 129.22, 120.01, 113.32, 81.68, 60.71, 37.75, 24.26, 15.81, 14.16. HRMS(ESI) (*m/z*): Calad. for C_20_H_23_NO_4_ 341.4009; found 296.1282 [M − C_2_H_5_O]^+^.

Methyl 4-((1-Ethoxy-2-oxo-2-(4-propylphenyl)ethyl)amino)benzoate (**6k**) methyl 4-aminobenzoate (0.50 g, 3.31 mmol) was added to a solution of 2-oxo-2-(4-propylphenyl)acetaldehyde (0.58 g, 3.31 mmol) in EtOH (20 mL), and the solution was stirred at room temperature overnight. The solvent was evaporated under reduced pressure, the crude residue was purified using silica gel chromatography and was eluted with PE/CH_3_COCH_3_ (v:v=8:1) to give 6k as white solid in a yield of 22%. ^1^H-NMR (400 MHz, DMSO-*d*_6_) δ 8.03 (d, *J* = 8.4 Hz, 2H), 7.78 (d, *J* = 8.8 Hz, 2H), 7.44 (d, *J* = 8.6 Hz, 1H), 7.37 (d, *J* = 8.4 Hz, 2H), 7.04 (d, *J* = 8.9 Hz, 2H), 6.28 (d, *J* = 8.6 Hz, 1H), 3.78 (s, 3H), 3.57–3.49 (m, 1H), 3.45–3.38 (m, 1H), 2.63 (t, *J* = 7.2 Hz, 2H), 1.66–1.57 (m, 2H), 1.04 (t, *J* = 7.0 Hz, 3H), 0.90 (t, *J* = 7.3 Hz, 3H). ^13^C-NMR (101 MHz, DMSO-*d*_6_) δ 193.71, 166.74, 150.97, 149.40, 132.49, 131.31, 129.69, 129.21, 118.86, 113.46, 81.62, 60.70, 51.95, 37.75, 24.26, 15.79, 14.15. HRMS(ESI) (*m/z*): Calad. for C_21_H_25_NO_4_ 355.4275; found 310.1438 [M − C_2_H_5_O]^+^.

2-Ethoxy-2-((4-nitrophenyl)amino)-1-(4-propylphenyl)ethanone (**6l**) ^1^H-NMR (400 MHz, DMSO-*d*_6_) δ 8.08 (d, *J* = 9.3 Hz, 2H), 8.04 (d, *J* = 8.4 Hz, 2H), 8.01 (s, 1H), 7.39 (d, *J* = 8.4 Hz, 2H), 7.11 (d, *J* = 9.3 Hz, 2H), 6.36 (d, *J* = 8.3 Hz, 1H), 3.59–3.52 (m, 1H), 3.46–3.39 (m, 1H), 2.64 (t, *J* = 7.2 Hz, 2H), 1.67–1.58 (m, 2H), 1.05 (t, *J* = 7.0 Hz, 3H), 0.90 (t, *J* = 7.3 Hz, 3H). ^13^C-NMR (101 MHz, DMSO-*d*_6_) δ 193.36, 153.02, 149.56, 138.35, 132.34, 129.76, 129.24, 126.32, 113.49, 81.51, 61.04, 37.76, 24.25, 15.75, 14.14. HRMS(ESI) (*m/z*): Calad. for C_19_H_22_N_2_O_4_ 342.3890; found 297.1234 [M − C_2_H_5_O]^+^.

2-Ethoxy-1-(4-hexylphenyl)-2-((4-nitrophenyl)amino)ethanone (**6m**) ^1^H-NMR (400 MHz, DMSO-*d*_6_) δ 8.07 (d, *J* = 8.8 Hz, 2H), 8.02 (d, *J* = 7.3 Hz, 2H), 7.99 (s, 1H), 7.38 (d, *J* = 7.7 Hz, 2H), 7.10 (d, *J* = 9.3 Hz, 2H), 6.35 (d, *J* = 8.3 Hz, 1H), 3.58–3.50 (m, 1H), 3.45–3.37 (m, 1H), 2.65 (t, *J* = 7.6 Hz, 2H), 1.58 (brs, 2H), 1.26 (brs, 6H), 1.04 (t, *J* = 7.0 Hz, 3H), 0.84 (brs, 3H). ^13^C-NMR (101 MHz, DMSO-*d*_6_) δ 193.39, 153.04, 149.82, 138.35, 132.31, 129.79, 129.21, 126.33, 113.50, 81.52, 61.06, 35.72, 31.59, 31.0, 28.85, 22.58, 15.75, 14.48. HRMS(ESI) (*m/z*): Calad. for C_22_H_28_N_2_O_4_ 384.4687; found 339.1704 [M − C_2_H_5_O]^+^.

4-((1-Ethoxy-2-(4-hexylphenyl)-2-oxoethyl)amino)benzoic Acid (**6n**) ^1^H-NMR (400 MHz, DMSO-*d*_6_) δ 12.29 (s, 1H), 8.01 (d, *J* = 8.4 Hz, 2H), 7.74 (d, *J* = 8.8 Hz, 2H), 7.38–7.34 (m, 3H), 7.00 (d, *J* = 8.9 Hz, 2H), 6.25 (d, *J* = 8.6 Hz, 1H), 3.55–3.48 (m, 1H), 3.44–3.39 (m, 1H), 2.65 (t, *J* = 7.2 Hz, 2H), 1.60–1.56 (m, 2H), 1.27 (brs, 6H), 1.03 (t, *J* = 7.0 Hz, 3H), 0.85 (t, *J* = 7.2 Hz, 3H). ^13^C-NMR (101 MHz, DMSO-*d*_6_) δ 193.76, 167.88, 150.62, 149.63, 132.49, 131.46, 129.70, 129.16, 120.01, 113.33, 81.68, 60.71, 35.71, 31.59, 31.05, 28.86, 22.58, 15.80, 14.48. HRMS(ESI) (*m/z*): Calad. for C_23_H_29_NO_4_ 383.4807; found 338.1749 [M − C_2_H_5_O]^+^.

4-((1-Ethoxy-2-(4-hexylphenyl)-2-oxoethyl)amino)-2-methylbenzoic Acid (**6o**) ^1^H-NMR (400 MHz, DMSO-*d*_6_) δ 12.09 (s, 1H), 8.01 (d, *J* = 7.7 Hz, 2H), 7.73 (d, *J* = 8.4 Hz, 1H), 7.37 (d, *J* = 7.2 Hz, 2H), 7.18 (d, *J* = 8.7 Hz, 1H), 6.83 (s, 2H), 6.25 (d, *J* = 8.8 Hz, 1H), 3.56–3.48 (m, 1H), 3.45–3.37 (m, 1H), 2.65 (t, *J* = 7.4 Hz, 2H), 2.48 (s, 3H), 1.59–1.56 (m, 2H), 1.27 (brs, 6H), 1.04 (t, *J* = 7.0 Hz, 3H), 0.85 (t, *J* = 5.7 Hz, 3H). ^13^C-NMR (101 MHz, DMSO-*d*_6_) δ 193.77, 168.68, 149.73, 149.59, 142.18, 133.17, 132.49, 129.69, 129.15, 119.04, 116.33, 111.02, 81.57, 60.68, 35.71, 31.59, 31.06, 28.86, 22.87, 22.58, 15.82, 14.48. HRMS(ESI) (*m/z*): Calad. for C_24_H_31_NO_4_ 397.5072; found 352.1909 [M − C_2_H_5_O]^+^.

4-((1-Ethoxy-2-(4-hexylphenyl)-2-oxoethyl)amino)-3-methylbenzoic Acid (**6p**) ^1^H-NMR (400 MHz, DMSO-*d*_6_) δ 12.27 (s, 1H), 8.04 (d, *J* = 8.2 Hz, 2H), 7.70–7.66 (m, 2H), 7.37 (d, *J* = 8.3 Hz, 2H), 7.06 (d, *J* = 8.6 Hz, 1H), 6.28 (d, *J* = 6.8 Hz, 2H), 3.57–3.50 (m, 1H), 3.39–3.36 (m, 1H), 2.65 (t, *J* = 7.6 Hz, 2H), 2.20 (s, 3H), 1.60–1.57 (m, 2H), 1.27 (brs, 6H), 1.02 (t, *J* = 7.0 Hz, 3H), 0.85 (t, *J* = 6.8 Hz, 3H). ^13^C-NMR (101 MHz, DMSO-*d*_6_) δ.194.10, 167.97, 149.72, 147.90, 132.30, 132.01, 129.83, 129.46, 129.12, 122.55, 120.18, 111.67, 81.79, 60.14, 35.72, 31.58, 31.02, 28.85, 22.58, 17.85, 15.75, 14.48. HRMS(ESI) (*m/z*): Calad. for C_24_H_31_NO_4_ 397.5072; found 352.1909 [M − C_2_H_5_O]^+^.

4-((1-Ethoxy-2-(4-octylphenyl)-2-oxoethyl)amino)benzoic Acid (**6q**) ^1^H-NMR (400 MHz, DMSO-*d*_6_) δ 12.26 (s, 1H), 8.01 (d, *J* = 8.3 Hz, 2H), 7.74 (d, *J* = 8.8 Hz, 2H), 7.38–7.33 (m, 3H), 7.00 (d, *J* = 8.9 Hz, 2H), 6.25 (d, *J* = 8.6 Hz, 1H), 3.55–3.48 (m, 1H), 3.43–3.37 (m, 1H), 2.65 (t, *J* = 7.6 Hz, 2H), 1.60–1.56 (m, 2H), 1.27–1.23 (m, 10H), 1.03 (t, *J* = 7.0 Hz, 3H), 0.85 (t, *J* = 6.8 Hz, 3H). ^13^C-NMR (101 MHz, DMSO-*d*_6_) δ 193.75, 167.88, 150.62, 149.62, 132.48, 131.46, 129.69, 129.16, 120.01, 113.32, 81.67, 60.70, 35.71, 31.80, 31.09, 29.33, 29.19, 22.62, 15.80, 14.50. HRMS(ESI) (*m/z*): Calad. for C_25_H_33_NO_4_ 411.5338; found 366.2067 [M − C_2_H_5_O]^+^.

4-((1-Ethoxy-2-(4-octylphenyl)-2-oxoethyl)amino)-2-methylbenzoic Acid (**6r**) ^1^H-NMR (400 MHz, DMSO-*d*_6_) δ 12.08 (s, 1H), 8.00 (d, *J* = 8.3 Hz, 2H), 7.73 (d, *J* = 9.2 Hz, 1H), 7.37 (d, *J* = 8.2 Hz, 2H), 7.17 (d, *J* = 8.7 Hz, 1H), 6.83–6.82 (m, 2H), 6.24 (d, *J* = 8.7 Hz, 1H), 3.56–3.48 (m, 1H), 3.45–3.37 (m, 1H), 2.65 (t, *J* = 7.6 Hz, 2H), 2.47 (s, 3H), 1.60–1.56 (m, 2H), 1.27–1.23 (m, 10H), 1.03 (t, *J* = 7.0 Hz, 3H), 0.85 (t, *J* = 6.8 Hz, 2H). ^13^C-NMR (101 MHz, DMSO-*d*_6_) δ 193.76, 168.68, 149.73, 149.59, 142.18, 133.17,132.49, 129.69, 129.14, 119.04, 116.34, 111.02, 81.57, 60.67, 35.71, 31.81, 31.10, 29.34, 29.20, 22.87, 22.63, 15.82, 14.50. HRMS(ESI) (*m/z*): Calad. for C_26_H_35_NO_4_ 425.5604; found 380.2222 [M − C_2_H_5_O]^+^.

4-((2-(4-Cyclohexylphenyl)-2-oxoethyl)amino)benzoic Acid (**8a**) ^1^H-NMR (400 MHz, DMSO-*d*_6_) δ 12.10 (s, 1H), 8.00 (d, *J* = 8.4 Hz, 2H), 7.67 (d, *J* = 8.9 Hz, 2H), 7.41 (d, *J* = 8.4 Hz, 2H), 6.70 (d, *J* = 8.9 Hz, 2H), 4.75 (s, 2H), 2.64–2.58 (m, 1H), 1.81–1.70 (m, 5H), 1.49–1.20 (m, 5H). ^13^C-NMR (101 MHz, DMSO-*d*_6_) δ 195.96, 168.03, 154.25, 152.61, 133.39, 131.51, 128.70, 127.67, 117.97, 112.01, 49.78, 44.41, 34.07, 26.73, 26.01. HRMS(ESI) (*m/z*): Calad. for C_21_H_23_NO_3_ 337.4122; found 338.1749 [M+H]^+^.

4-((2-Oxo-2-(4-propylphenyl)ethyl)amino)benzoic Acid (**8b**) ^1^H-NMR (400 MHz, DMSO-*d*_6_) δ 12.07 (s, 1H), 8.00 (d, *J* = 8.2 Hz, 2H), 7.68 (d, *J* = 8.8 Hz, 2H), 7.39 (d, *J* = 8.2 Hz, 2H), 6.71–6.67 (m, 3H), 4.75 (s, 2H), 2.65 (t, *J* = 7.6 Hz, 2H), 1.67–1.58 (m, 2H), 0.90 (t, *J* = 7.3 Hz, 3H). ^13^C-NMR (101 MHz, DMSO-*d*_6_) δ 195.97, 168.05, 152.61, 149.11, 133.25, 131.52, 129.30, 128.59, 118.00, 112.02, 49.79, 37.73, 24.35, 14.14. HRMS(ESI) (*m/z*): Calad. for C_18_H_19_NO_3_ 297.3484; found 298.1438 [M + H]^+^.

4-((2-(4-Hexylphenyl)-2-oxoethyl)amino)benzoic Acid (**8c**) ^1^H-NMR (400 MHz, DMSO-*d*_6_) δ 12.07 (s, 1H), 8.00 (d, *J* = 8.2 Hz, 2H), 7.67 (d, *J* = 8.6 Hz, 2H), 7.38 (d, *J* = 8.2 Hz, 2H), 6.70 (d, *J* = 8.8 Hz, 2H), 6.67 (t, *J* = 5.2 Hz, 1H), 4.74 (d, *J* = 5.2 Hz, 2H), 2.66 (t, *J* = 7.6 Hz, 2H), 1.63–1.55 (m, 2H), 1.28 (brs, 6H), 0.86 (t, *J* = 6.8 Hz, 3H). ^13^C-NMR (101 MHz, DMSO-*d*_6_) δ 195.98, 168.03, 152.62, 149.35, 133.23, 131.51, 129.24, 128.61, 118.00, 112.02, 49.79, 35.68, 31.61, 31.14, 28.84, 22.60, 14.50. HRMS(ESI) (*m/z*): Calad. for C_21_H_25_NO_3_ 339.4281; found 340.1907 [M + H]^+^.

4-((2-(4-Octylphenyl)-2-oxoethyl)amino)benzoic Acid (**8d**) ^1^H-NMR (400 MHz, DMSO-*d*_6_) δ 12.06 (s, 1H), 7.99 (d, *J* = 8.3 Hz, 2H), 7.67 (d, *J* = 8.9 Hz, 2H), 7.38 (d, *J* = 8.2 Hz, 2H), 6.70 (d, *J* = 8.8 Hz, 2H), 6.66 (t, *J* = 5.2 Hz, 1H), 4.74 (d, *J* = 5.4 Hz, 2H), 2.66 (t, *J* = 7.2 Hz, 2H), 1.63–1.55 (m, 2H), 1.29–1.24 (m, 10H), 0.85 (t, *J* = 7.2 Hz, 3H). ^13^C-NMR (101 MHz, DMSO-*d*_6_) δ 195.97, 168.02, 152.61, 149.35, 133.22, 131.50, 129.24, 128.60, 117.99, 112.01, 49.79, 35.68, 31.82, 31.18, 29.35, 29.21, 29.18, 22.63, 14.51. HRMS(ESI) (*m/z*): Calad. for C_23_H_29_NO_3_ 367.4813; found 368.2221 [M + H]^+^.

4-(2-(4-Cyclohexylphenyl)-2-oxoeacetamido)benzoic Acid (**9a**) ^1^H-NMR (400 MHz, DMSO-*d*_6_) δ 12.88 (s, 1H), 11.23 (s, 1H), 7.97 (d, *J* = 8.7 Hz, 4H), 7.86 (d, *J* = 8.7 Hz, 2H), 7.48 (d, *J* = 8.2 Hz, 2H), 2.66–2.60 (m, 1H), 1.82–1.70 (m, 5H), 1.49–1.23 (m, 5H). ^13^C-NMR (101 MHz, DMSO-*d*_6_) δ 189.22, 167.35, 164.40, 155.85, 142.25, 131.03, 130.83, 130.81, 128.08, 126.96, 120.01, 44.51, 33.95, 26.67, 25.98. HRMS(ESI) (*m/z*): Calad. for C_21_H_21_NO_4_ 351.3957; found 352.1542 [M + H]^+^.

4-(2-Oxo-2-(4-propylphenyl)acetamido)benzoic Acid (**9b**) ^1^H-NMR (400 MHz, DMSO-*d*_6_) δ 12.89 (s, 1H), 11.25 (s, 1H), 7.99–7.97 (m, 4H), 7.86 (d, *J* = 8.9 Hz, 2H), 7.44 (d, *J* = 8.3 Hz, 2H), 2.67 (t, *J* = 7.2 Hz, 2H), 1.68–1.58 (m, 2H), 0.90 (t, *J* = 7.3 Hz, 3H). ^13^C-NMR (101 MHz, DMSO-*d*_6_) δ 189.24, 167.35, 164.41, 150.80, 142.25, 131.03, 130.70, 129.70, 126.96, 120.01, 37.83, 24.24, 14.10. HRMS(ESI) (*m/z*): Calad. for C_18_H_17_NO_4_ 311.3319; found 312.1230 [M + H]^+^.

4-(2-(4-Hexylphenyl)-2-oxoacetamido)benzoic Acid (**9c**) ^1^H-NMR (400 MHz, DMSO-*d*_6_) δδ 12.89 (s, 1H), 11.24 (s, 1H), 7.98–7.96 (m, 4H), 7.86 (d, *J* = 8.9 Hz, 2H), 7.44 (d, *J* = 8.3 Hz, 2H), 2.69 (t, *J* = 7.2 Hz, 2H), 1.63–1.56 (m, 2H), 1.28 (brs, 6H), 0.85 (t, *J* = 6.8 Hz, 3H). ^13^C-NMR (101 MHz, DMSO-*d*_6_) δ 189.23, 167.36, 164.42, 151.06, 142.24, 131.02, 130.71, 130.65, 129.64, 126.98, 120.00, 35.81, 31.58, 31.02, 28.81, 22.58, 14.49. HRMS(ESI) (*m/z*): Calad. for C_21_H_23_NO_4_ 353.4116; found 354.1700 [M + H]^+^.

4-(2-(4-Octylphenyl)-2-oxoacetamido)benzoic Acid (**9d**) ^1^H-NMR (400 MHz, DMSO-*d*_6_) δ 12.89 (s, 1H), 11.24 (s, 1H), 7.98–7.96 (m, 4H), 7.86 (d, *J* = 8.8 Hz, 2H), 7.44 (d, *J* = 8.2 Hz, 2H), 2.68 (t, *J* = 8.0 Hz, 2H), 1.61–1.58 (m, 2H), 1.27–1.24 (m, 10H), 0.85 (t, *J* = 6.8 Hz, 3H). ^13^C-NMR (101 MHz, DMSO-*d*_6_) δ 189.23, 167.36, 164.41, 151.06, 142.24, 131.02, 130.72, 130.65, 129.64, 126.99, 120.01, 35.81, 31.80, 31.06, 29.33, 29.18, 29.16, 22.62, 14.50. HRMS(ESI) (*m/z*): Calad. for C_23_H_27_NO_4_ 381.4648; found 382.2011 [M + H]^+^.

### 3.3. Biological Assay

#### 3.3.1. Determination of the MIC of Colistin

The MIC of colistin was determined using the broth microdilution method. *E. coli* BL21(DE3) and pET-28a(+) were obtained from commercial sources (YouBio). The full length of the colistin-resistance gene *mcr-1* was synthesized by the Sangon Biotech (Shanghai) Co., Ltd. Antibiotics, culture media, and other reagents used were purchased commercially. Bacterial colonies were selected from a LB agar and transferred to a cation-adjusted Mueller-Hinton broth (CAMHB) tube, and the broth was shaken at 37 °C. After adjusting the turbidity to OD600 0.08-0.1 (1 × 10^8^ cfu·mL^−1^), the suspensions were further diluted (1:100) to obtain an inoculum of 1 × 10^6^ cfu·mL^−1^. The working concentrations of colistin (ranging from 0.0625 μg·mL^−1^ to 64 μg·mL^−1^ in 2-fold dilutions) were prepared in CAMHB. 50 μL of each intermediate concentration of colistin solution was dispensed into the wells of the microwell plates, and 50 μL of the bacterial suspension was added to the 96-well microplates. The final bacterial concentration is 5 × 10^5^ cfu·mL^−1^. The plates were incubated in an ambient air incubator at 37 °C for 16 h. The MIC was determined as the lowest concentration that completely inhibits bacterial growth in the wells, and the data was obtained in at least two independent experiments.

#### 3.3.2. The Susceptibility Testing of Colistin Combined with Compounds

The broth microdilution method was applied to identify potential inhibitors of MCR-1 in the presence of 2 μg·mL^−1^ colistin in CAMHB. Dimethyl sulfoxide (DMSO) was added with a final concentration of 0.1%. As described above, bacterial colonies were selected from a LB agar and transferred to a CAMHB tube, and the broth was shaken at 37 °C. After adjusting the turbidity to OD600 0.08–0.1 (1 × 10^8^ cfu·mL^−1^), the suspensions were further diluted (1:100) to obtain an inoculum of 1 × 10^6^ cfu·mL^−1^. The working concentrations of compounds (ranging from 50 μΜ to 400 μΜ in 2-fold dilutions) were prepared in CAMHB. 50 μL of the bacterial suspension was added to the 96-well microplates, and 25 μL of each intermediate concentration of the compound solution was dispensed into the wells of the microwell plates. Then, 25 μL of the prepared colistin solution was added into the microwell plates. The final concentrations of compounds were 12.5 μΜ, 25 μΜ, 50 μΜ, and 100 μΜ. The final bacterial concentration is 5 × 10^5^ cfu·mL^−1^. The plates were incubated in an ambient air incubator at 37 °C for 16 h. The activity was evaluated by recording the lowest concentrations of compounds in combination with 2 μg·mL^−1^ colistin that completely inhibited the visible growth of the bacteria, and the data was obtained in at least two independent experiments.

#### 3.3.3. The Assay for MCR-1 Enzymatic Activity

In this biochemical assay, the fluorescently-labeled substrate (NBD-PEA) is 1-acyl-2-{12-[(7-nitro-2-1,3-benzoxadiazol-4-yl) amino] dodecanoyl}-sn-glycero-3-phosphoethanol amine (Avanti Lipids, USA). The reaction system (10 μL in total) was kept for 18 h at room temperature and contained 50 mM HEPES (pH 7.50), 100 mM NaCl, 0.03% of DDM, 0.2 mM NBD-PEA, 4 μM MCR-1 and compounds (compound 3 is 2 mM, 5 mM, 10 mM; compound 6p is 0.1 mM, 0.4 mM, 1.6 mM; compound 6q is 0.2 mM, 0.4mM, 0.8 mM; compound 6m is 1 mM, 5 mM, 10 mM). The reaction products were subjected to TLC in a mobile phase consisting of ethyl acetate: methanol: water (*v*:*v*:*v* = 7:2:1). The fluorescent signal on the TLC plate was detected under Epi blue light (455–485 nm) with a gel imaging system (Bio-Rad), and the data was obtained in at least two independent experiments.

### 3.4. Molecular Docking

The full-length structure of MCR-1 was modelled by the software Swiss-Model using the structure of *Neisseria meningitidis* EptA (PDB: 5FGN) as a template, and the molecular docking was described as Section 3.1. After the docking process was finished, the best pose of each molecule was used to analyze the interactions and study the relationship between the compounds and the MCR-1 protein.

## 4. Conclusions

This study shows that a combination of colistin and MCR-1 inhibitors could be an alternative option for reverting colistin resistance, and this strategy may not place selective pressure on bacterium. The present research on MCR-1 inhibitors (such as ETA and pterostilbene) exhibited low activity, and the mechanism remains to be further clarified. To solve the issue of polymyxins resistance caused by *mcr-1*, it is important to develop new agents for high-efficiency against MCR-1. In this work, we identified racemic compound **3** as a potential MCR-1 inhibitor by structure-based virtual screening for the first time, and 26 novel compounds were designed and synthesized based on the structure of 1-phenyl-2-(phenylamino) ethanone. In the evaluation of cell-based assay, seven compounds (**6g**, **6h**, **6i**, **6n**, **6p**, **6q**, and **6r**) displayed more potent activity than compound **3**. Among them, 25 μΜ of compounds **6p** or **6q** in combination with 2 μg·mL^−1^ colistin could completely inhibit the growth of BL21(DE3) expressing *mcr-1*, which exhibited the most potent activity. Moreover, in the enzymatic assay, we elucidate that compound **6p** and **6q** could target the MCR-1 and inhibit the activity of the protein. Additionally, molecular docking studies on several compounds were carried out to elucidate the SARs, and the docking results exhibited that the active compounds could interact with Glu246 and Thr285 via hydrogen bonds, and they occupy well the cavity of MCR-1 protein. Therefore, we conclude that the essential hydrogen bonding and appropriate substitution size could promote the inhibitory activity against MCR-1 protein. We are doing research on further structural optimization, and we will look for valuable information to develop potential MCR-1 inhibitors.

## Figures and Tables

**Table 1:** The colistin MIC of *Escherichia* *coli* BL21(DE3) strains that carry the *mcr-1* gene.

*E. coli* BL21(DE3) Strains	Colistin MIC (μg·mL^−1^)
BL21(DE3)	2
pET28a	2
pET28a-*mcr-1*	8

**Table 2:**
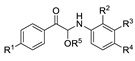
The activities of **6a–6r** in combination with 2 μg·mL^−1^ colistin against the growth of BL21(DE3) expressing *mcr-1*.

Comp.	R^1^	R^2^	R^3^	R^4^	R^5^	Conc. (μM)
**3**	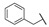	H	H	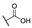	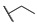	100
**6a**	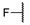	H	H	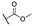	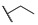	N.I.A.
**6b**	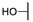	H	H	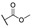	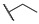	N.I.A.
**6c**	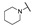	H	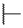	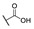	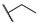	N.I.A.
**6d**	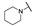	H	H	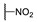	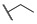	N.I.A.
**6e**	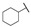	H	H	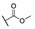	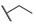	N.I.A.
**6f**	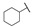	H	H	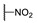	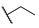	N.I.A.
**6g**	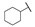	H	H	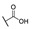	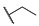	50
**6h**	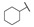	H	H	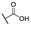	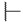	50
**6i**	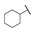	H	H	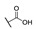	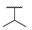	50
**6j**	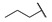	H	H	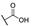	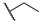	N.I.A.
**6k**	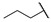	H	H	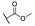	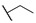	N.I.A.
**6l**	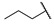	H	H	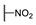	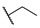	N.I.A.
**6m**	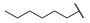	H	H	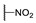	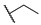	N.I.A.
**6n**	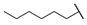	H	H	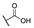	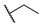	50
**6o**	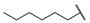	H	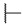	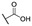	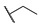	100
**6p**	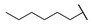	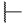	H	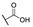	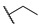	25
**6q**	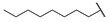	H	H	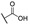	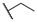	25
**6r**	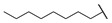	H	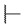	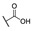	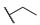	50

N.I.A. = No Inhibitory activity.

**Table 3:**
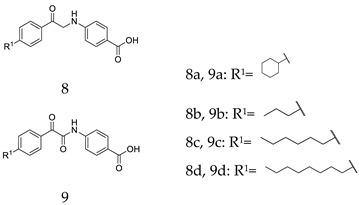
The activities of **8a–8d** and **9a–9d** in combination with 2 μg·mL^−1^ colistin against the growth of BL21(DE3) expressing *mcr-1*.

Comp.	Conc. (μM)	Comp.	Conc. (μM)
**8a**	N.I.A.	**9a**	N.I.A.
**8b**	N.I.A.	**9b**	N.I.A.
**8c**	N.I.A.	**9c**	N.I.A.
**8d**	N.I.A.	**9d**	N.I.A.

N.I.A.= No Inhibitory activity.

## Supplementary Materials

The following are available online.
