# Supplementary material for: Design, Synthesis and Biological Evaluation of 1-Phenyl-2-(phenylamino) Ethanone Derivatives as Novel MCR-1 Inhibitors
Source: Molecules. 2019 Jul 26;24(15):2719. doi: 10.3390/molecules24152719 (PMC6696459; doi:10.3390/molecules24152719)
Supplement: Supplementary file 1 [file molecules-24-02719-s001.pdf]

Supplementary data

Design, synthesis and biological evaluation of 1-phenyl-2-(phenylamino) ethanone derivatives as novel MCR-1 inhibitors

Xiu-juan Lan [1,2,3], Hai-tao Yan [3], Feng Lin [4], Shi Hou [1], Chen-chen Li [1], Guang-shu Wang [1,*], Wei Sun [1,*], Jun-hai Xiao [2,3,*], Song Li [2,3]

1 School of Pharmaceutical Sciences, Jilin University, Changchun 130021, China; xiujiuanlan@139.com (X.-j.L.); houshi28@126.com (S.H.); lichenchen18@126.com (C.-c.L.); wgs@jlu.edu.cn (G.-s.W); wsun@jlu.edu.cn (W.S.)

2 National Engineering Research Center for Strategic Drugs, Beijing Institute of Pharmacology and Toxicology, Beijing 100850, China

3 State Key Laboratory of Toxicology and Medical Countermeasures, Beijing Institute of Pharmacology and Toxicology, Beijing 100850, China; yanht7809@aliyun.com (H.-t.Y.); xiaojunhai@139.com (J.-h.X.); lis@nic.bmi.ac.cn (S.L.)

4 School of Life Sciences, Jilin University, Changchun 130021, China; linfeng@jlu.edu.cn (F.L.)

* Correspondence: wgs@jlu.edu.cn (G.-s.W); wsun@jlu.edu.cn (W.S.); xiaojunhai@139.com (J.-h.X.)
Tel.: +86-10-6693-1642 (J.-h.X.)

Table of Contents:

The synthesis of the compounds 6a-6r, 8a-8d and 9a-9d.

Fig S1-S26: The compounds characterization of the ^{1}H NMR and ^{13}C NMR.

Fig S27-S52: The compounds characterization of the HRMS.

Fig.S53 Docking patterns of the interaction between compounds A: 6q; B: 6n; C: 6p; D: 6o and the full-length MCR-1 protein.

*methyl 4-((1-ethoxy-2-(4-fluorophenyl)-2-oxoethyl)amino)benzoate (**6a**)*

To a solution of SeO_2 (3.21 g, 0.03 mol), 1,4-dioxane (20 mL) and H_2O (1 mL) (v:v=20:1), 1-(4-fluorophenyl)ethanone (2.00 g, 0.014 mol) was added, and the mixture was refluxed under nitrogen for 9 h. The solid residue was removed by filtration. The solvent was removed under reduced pressure. Water (20 mL) was added and the resulting mixture was heated to reflux for 5 h. Upon cooling to room temperature, the solution was extracted with ethyl acetate and dried over Na_2SO_4. The organic layer was evaporated to dryness. The crude residue was isolated using silica gel chromatography and was eluted with PE/CH_3COCH_3 (v:v=10:1) to give 2-(4-fluorophenyl)-2-oxoacetaldehyde in a yield of 78%. Then, methyl 4-aminobenzoate (0.50 g, 3.31 mmol) was added to a solution of 2-(4-fluorophenyl)-2-oxoacetaldehyde (0.50 g, 3.31 mmol) in EtOH (20 mL), and the solution was stirred at room temperature overnight. After removing partial solvent, the precipitated solid was filtered to give 6a as white solid in a yield of 30%.

*methyl 4-((1-ethoxy-2-(4-hydroxyphenyl)-2-oxoethyl)amino)benzoate (**6b**)*

To a solution of SeO_2 (3.26 g, 0.03 mol), 1,4-dioxane (20 mL) and H_2O (1 mL) (v:v=20:1), 1-(4-hydroxyphenyl)ethanone (2.00 g, 0.015 mol) was added, and the mixture was refluxed under nitrogen for 9 h. The solid residue was removed by filtration. The solvent was removed under reduced pressure. Water (20 mL) was added and the resulting mixture was heated to reflux for 5 h. Upon cooling to room temperature, the solution was extracted with ethyl acetate and dried over Na_2SO_4. The organic layer was evaporated to dryness. The crude residue was isolated using silica gel chromatography and was eluted with DCM/CH_3OH (v:v=60:1) to give 2-(4-hydroxyphenyl)-2-oxoacetaldehyde in a yield of 76%. Then, methyl 4-aminobenzoate (0.50 g, 3.31 mmol) was added to a solution of 2-(4-hydroxyphenyl)-2-oxoacetaldehyde (0.50 g, 3.31 mmol) in EtOH (20 mL), and the solution was stirred at room temperature overnight. The precipitated solid was obtained by filtration and purified by recrystallization from EtOH to give 6b as white solid in a yield of 11%.

*4-((1-ethoxy-2-oxo-2-(4-(piperidin-1-yl)phenyl)ethyl)amino)-2-methylbenzoic acid (**6c**)*

To a solution of SeO_2 (2.18 g, 0.02 mol), 1,4-dioxane (20 mL) and H_2O (1 mL) (v:v=20:1), 1-(4-(piperidin-1-yl)phenyl)ethanone (2.00 g, 0.01 mol) was added, and the mixture was refluxed under nitrogen for 9 h. The solid residue was removed by filtration. The solvent was removed under reduced pressure. Water (20 mL) was added and the resulting mixture was heated to reflux for 5 h. Upon cooling to room temperature, the solution was extracted with ethyl acetate and dried over Na_2SO_4. The organic layer was evaporated to dryness. The crude residue was isolated using silica gel chromatography and was eluted with PE/CH_3COCH_3 (v:v=8:1) to give 2-oxo-2-(4-(piperidin-1-yl)phenyl)acetaldehyde in a yield of 75%. Then, 4-amino-2-methylbenzoic acid (0.50 g, 3.31 mmol) was added to a solution of 2-oxo-2-(4-(piperidin-1-yl)phenyl) acetaldehyde (0.72 g, 3.31 mmol) in EtOH (20 mL), and the solution was stirred at room temperature overnight. The precipitated solid was obtained by filtration and purified by recrystallization from EtOH to give 6c as brown solid in a yield of 15%.

*2-ethoxy-2-((4-nitrophenyl)amino-1-(4-(piperidin-1-yl)phenyl)ethanone (**6d**)*

4-nitroaniline (0.50 g, 3.62 mmol) was added to a solution of 2-oxo-2-(4-(piperidin-1-yl)phenyl)acetaldehyde (0.79 g, 3.62 mmol) in EtOH (20 mL), and the solution was stirred at room temperature overnight. The precipitated solid was obtained by filtration and purified using silica gel chromatography, eluting with PE/CH_3COCH_3 (v:v=10:1) to give 6d as yellow solid in a yield of 24%.

*methyl 4-((2-(4-cyclohexylphenyl)-1-ethoxy-2-oxoethyl)amino)benzoate (**6e**)*

To a solution of SeO_2 (2.20 g, 0.02 mol), 1,4-dioxane (20 mL) and H_2O (1 mL) (v:v=20:1), 1-(4-cyclohexylphenyl)ethanone (2.00 g, 0.01 mol) was added, and the mixture was refluxed under nitrogen for 9 h. The solid residue was removed by filtration. The solvent was removed under reduced pressure. Water (20 mL) was added and the resulting mixture was heated to reflux for 5 h. Upon cooling to room temperature, the precipitated solid was obtained by filtration, the crude residue was isolated using silica gel chromatography and was eluted with PE/CH_3COCH_3 (v:v=15:1) to give 2-(4-cyclohexylphenyl)-2-oxoacetaldehyde in a yield of 79%. Then, methyl 4-amino benzoate (0.50 g, 3.31 mmol) was added to a solution of 2-(4-cyclohexylphenyl)-2-oxoacetaldehyde (0.70 g, 3.31 mmol) in EtOH (20 mL), and the solution was stirred at room temperature overnight. The solvent was evaporated under reduced pressure, the crude residue was purified using silica gel chromatography and was eluted with PE/CH_3COCH_3 (v:v=40:1) to give 6e as white solid in a yield of 25%.

*1-(4-cyclohexylphenyl)-2-ethoxy-2-((4-nitrophenyl)amino)ethanone (**6f**)*

4-nitroaniline (0.50 g, 3.62 mmol) was added to a solution of 2-(4-cyclohexylphenyl)-2-oxoacetaldehyde (0.78 g, 3.62 mmol) in EtOH (20 mL), and the solution was stirred at room temperature overnight. The solvent was evaporated under reduced pressure, the crude residue was purified using silica gel chromatography and was eluted with PE/EtOAc (v:v=30:1) to give 6f as yellow solid in a yield of 28%.

*4-((2-(4-cyclohexylphenyl)-1-ethoxy-2-oxoethyl)amino)benzoic acid (**6g**)*

4-aminobenzoic acid (0.50 g, 3.65 mmol) was added to a solution of 2-(4-cyclohexylphenyl)-2-oxoacetaldehyde (0.79 g, 3.65 mmol) in EtOH (20 mL), and the solution was stirred at room temperature overnight. The precipitated solid was obtained by filtration and purified by recrystallization from EtOH to give 6g as white solid in a yield of 10.8%.

*4-((2-(4-cyclohexylphenyl)-1-methoxy-2-oxoethyl)amino)benzoic acid (**6h**)*

4-aminobenzoic acid (0.50 g, 3.65 mmol) was added to a solution of 2-(4-cyclohexylphenyl)-2-oxoacetaldehyde (0.79 g, 3.65 mmol) in CH_3OH (20 mL), and the solution was stirred at room temperature overnight. The precipitated solid was obtained by filtration and purified by recrystallization from CH_3OH to give 6h as grey solid in a yield of 12.6%.

*4-((2-(4-cyclohexylphenyl)-1-isopropoxy-2-oxoethyl)amino)benzoic acid (**6i**)*

4-aminobenzoic acid (0.50 g, 3.65 mmol) was added to a solution of

2-(4-cyclohexylphenyl)-2-oxoacetaldehyde (0.79 g, 3.65 mmol) in $(CH_3)_2CHOH$ (20 mL), and the solution was stirred at room temperature overnight. The precipitated solid was obtained by filtration and purified by recrystallization from $(CH_3)_2CHOH$ to give 6i as white solid in a yield of 11.8%.

*4-((1-ethoxy-2-oxo-2-(4-propylphenyl)ethyl)amino)benzoic acid (**6j**)*
To a solution of SeO_2 (2.70 g, 0.02 mol), 1,4-dioxane (20 mL) and H_2O (1 mL) (v:v=20:1), 1-(4-propylphenyl)ethanone (2.00 g, 0.01 mol) was added, and the mixture was refluxed under nitrogen for 9 h. The solid residue was removed by filtration. The solvent was removed under reduced pressure. Water (20 mL) was added and the resulting mixture was heated to reflux for 5 h. Upon cooling to room temperature, the solution was extracted with ethyl acetate and dried over Na_2SO_4 The organic layer was evaporated to dryness. The crude residue was isolated using silica gel chromatography and was eluted with PE/EtOAc (v:v=5:1) to give 2-oxo-2-(4-propylphenyl)acetaldehyde in a yield of 80%. Then 4-aminobenzoic acid (0.50 g, 3.65 mmol) was added to a solution of 2-oxo-2-(4-propylphenyl)acetaldehyde (0.64 g, 3.65 mmol) in EtOH (20 mL), and the solution was stirred at room temperature overnight. The precipitated solid was obtained by filtration and purified by recrystallization from EtOH to give 6j as white solid in a yield of 13.6%.

*methyl 4-((1-ethoxy-2-oxo-2-(4-propylphenyl)ethyl)amino)benzoate (**6k**)*
methyl 4-aminobenzoate (0.50 g, 3.31 mmol) was added to a solution of 2-oxo-2-(4-propylphenyl)acetaldehyde (0.58 g, 3.31 mmol) in EtOH (20 mL), and the solution was stirred at room temperature overnight. The solvent was evaporated under reduced pressure, the crude residue was purified using silica gel chromatography and was eluted with PE/CH_3COCH_3 (v:v=8:1) to give 6k as white solid in a yield of 22%.

*2-ethoxy-2-((4-nitrophenyl)amino)-1-(4-propylphenyl)ethanone (**6l**)*
4-nitroaniline (0.50 g, 3.62 mmol) was added to a solution of 2-oxo-2-(4-propylphenyl)acetaldehyde (0.64 g, 3.62 mmol) in EtOH (20 mL), and the solution was stirred at room temperature overnight. The solvent was evaporated under reduced pressure, the crude residue was purified using silica gel chromatography and was eluted with PE/EtOAc (v:v=50:1) to give 6l as yellow solid in a yield of 27.3%.

*2-ethoxy-1-(4-hexylphenyl)-2-((4-nitrophenyl)amino)ethanone (**6m**)*
To a solution of SeO_2 (2.17 g, 0.02 mol), 1,4-dioxane (20 mL) and H_2O (1 mL) (v:v = 20:1), 1-(4-hexylphenyl)ethanone (2.00 g, 0.01 mol) was added, and the mixture was refluxed under nitrogen for 9 h. The solid residue was removed by filtration. The solvent was removed under reduced pressure. Water (20 mL) was added and the resulting mixture was heated to reflux for 5 h. Upon cooling to room temperature, the precipitated solid was obtained by filtration, the crude residue was isolated using silica gel chromatography and was eluted with PE/CH_3COCH_3 (v:v=5:1) to give 2-(4-hexylphenyl)-2-oxoacetaldehyde in a yield of 77%. Then 4-nitroaniline (0.50 g, 3.62 mmol) was added to a solution of 2-(4-hexylphenyl)-2-oxoacetaldehyde (0.80 g, 3.62 mmol) in EtOH (20 mL), and the solution was stirred at room temperature overnight. The solvent was evaporated under reduced

pressure, the crude residue was purified using silica gel chromatography and was eluted with PE/EtOAc (v:v = 15:1) to give 6m as yellow solid in a yield of 25.1%.

*4-((1-ethoxy-2-(4-hexylphenyl)-2-oxoethyl)amino)benzoic acid (**6n**)*

4-aminobenzoic acid (0.50 g, 3.65 mmol) was added to a solution of 2-(4-hexylphenyl)-2-oxoacetaldehyde (0.80 g, 3.65 mmol) in EtOH (20 mL), and the solution was stirred at room temperature overnight. The solvent was evaporated partially under reduced pressure, and then, the precipitated solid was obtained by filtration and purified by recrystallization from EtOH to give 6n as white solid in a yield of 10.8%.

*4-((1-ethoxy-2-(4-hexylphenyl)-2-oxoethyl)amino)-2-methylbenzoic acid (**6o**)*

4-amino-2-methylbenzoic acid (0.50 g, 3.31 mmol) was added to a solution of 2-(4-hexylphenyl)-2-oxoacetaldehyde (0.72 g, 3.31 mmol) in EtOH (20 mL), and the solution was stirred at room temperature overnight. The precipitated solid was obtained by filtration and purified by recrystallization from EtOH to give 6o as white solid in a yield of 12.6%.

*4-((1-ethoxy-2-(4-hexylphenyl)-2-oxoethyl)amino)-3-methylbenzoic acid (**6p**)*

4-amino-3-methylbenzoic acid (0.50 g, 3.31 mmol) was added to a solution of 2-(4-hexylphenyl)-2-oxoacetaldehyde (0.72 g, 3.31 mmol) in EtOH (20 mL), and the solution was stirred at room temperature overnight. After removing partial solvent, the precipitated solid was obtained by filtration and purified by recrystallization from EtOH to give 6p as white solid in a yield of 10%.

*4-((1-ethoxy-2-(4-octylphenyl)-2-oxoethyl)amino)benzoic acid (**6q**)*

To a solution of SeO_2 (1.91 g, 0.017 mol), 1,4-dioxane (20 mL) and H_2O (1 mL) (v:v = 20:1), 1-(4-octylphenyl)ethanone (2.00 g, 0.008 mol) was added and the mixture was refluxed under nitrogen for 9 h. The solid residue was removed by filtration. The solvent was removed under reduced pressure. Water (20 mL) was added, and the resulting mixture was heated to reflux for 5 h. Upon cooling to room temperature, the precipitated solid was obtained by filtration, the crude residue was isolated using silica gel chromatography and was eluted with PE/CH_3COCH_3 (v:v=5:1) to give 2-(4-octylphenyl)-2-oxoacetaldehyde in a Yield of 78%. Then 4-aminobenzoic acid (0.50 g, 3.65 mmol) was added to a solution of 2-(4-octylphenyl)-2-oxoacetaldehyde (0.90 g, 3.65 mmol) in EtOH (20 mL), and the solution was stirred at room temperature overnight. The solvent was evaporated partially under reduced pressure, and then, the precipitated solid was obtained by filtration and purified by recrystallization from EtOH to give 6q as white solid in a yield of 23%.

*4-((1-ethoxy-2-(4-octylphenyl)-2-oxoethyl)amino)-2-methylbenzoic acid (**6r**)*

4-amino-2-methylbenzoic acid (0.50 g, 3.31 mmol) was added to a solution of 2-(4-hexylphenyl)-2-oxoacetaldehyde (0.80 g, 3.31 mmol) in EtOH (20 mL), and the solution was stirred at room temperature overnight. The precipitated solid was obtained by filtration and purified by recrystallization from EtOH to give 6r as white solid in a yield of 26%.

*4-((2-(4-cyclohexylphenyl)-2-oxoethyl)amino)benzoic acid (**8a**)*

To a solution of $CuBr_2$ (5.52 g, 0.025 mol) in EtOAc (20 mL), 1-(4-cyclohexylphenyl)ethanone (2.00 g, 0.01 mol) was added, and the mixture was refluxed for 8 h. The solid residue was removed by filtration. The solvent was washed with 5% H_3PO_4, water and brine, while the organic layer was dried over Na_2SO_4. The crude residue was isolated using silica gel chromatography and was eluted with PE/ EtOAc (v:v=200:1) to give 2-bromo-1-(4-cyclohexylphenyl)ethanone in a yield of 43%. Then, 4-aminobenzoic acid (0.50 g, 3.65 mmol) was added to a solution of 2-bromo-1-(4-cyclohexylphenyl) ethanone (1.05 g, 3.65 mmol) in EtOH (20 mL), and the solution was stirred at room temperature overnight. The precipitated solid was obtained by filtration to give 8a as yellow solid in a yield of 22%.

*4-((2-oxo-2-(4-propylphenyl)ethyl)amino)benzoic acid (**8b**)*

To a solution of $CuBr_2$ (6.88 g, 0.031 mol) in EtOAc(20 mL), 1-(4-propylphenyl) ethanone (2.00 g, 0.012 mol) was added, and the mixture was refluxed for 8 h. The solid residue was removed by filtration. The solvent was washed with 5% H_3PO_4, water and brine, while the organic layer was dried over Na_2SO_4. The crude residue was isolated using silica gel chromatography and was eluted with PE/ CH_3COCH_3 (v:v=200:1) to give 2-bromo-1-(4-propylphenyl)ethanone in a yield of 44%. Then 4-aminobenzoic acid (0.50 g, 3.65 mmol) was added to a solution of 2-bromo-1-(4-propylphenyl)ethanone (0.88 g, 3.65 mmol) in EtOH (20 mL), and the solution was stirred at room temperature overnight. The precipitated solid was obtained by filtration to give 8b as yellow solid in a yield of 20%.

*4-((2-(4-hexylphenyl)-2-oxoethyl)amino)benzoic acid (**8c**)*

To a solution of $CuBr_2$ (5.47 g, 0.025 mol) in EtOAc (20 mL), 1-(4-hexylphenyl)ethanone (2.00 g, 0.01 mol) was added, and the mixture was refluxed for 8 h. The solid residue was removed by filtration. The solvent was washed with 5% H_3PO_4, water and brine, while the organic layer was dried over Na_2SO_4. The crude residue was isolated using silica gel chromatography and was eluted with PE/EtOAc (v:v=200:1) to give 2-bromo-1-(4-hexylphenyl)ethanone in a yield of 44%. Then 4-aminobenzoic acid (0.50 g, 3.65 mmol) was added to a solution of 2-bromo-1-(4-hexylphenyl)ethanone (1.03 g, 3.65 mmol) in EtOH (20 mL), and the solution was stirred at room temperature overnight. The precipitated solid was obtained by filtration to give 8c as yellow solid in a yield of 19%.

*4-((2-(4-octylphenyl)-2-oxoethyl)amino)benzoic acid (**8d**)*

To a solution of $CuBr_2$ (4.80 g, 0.022 mol) in EtOAc(20 mL), 1-(4-octylphenyl)ethanone (2.00 g, 0.009 mol) was added, and the mixture was refluxed for 8 h. The solid residue was removed by filtration. The solvent was washed with 5% H_3PO_4, water and brine, while the organic layer was dried over Na_2SO_4. The crude residue was isolated using silica gel chromatography and was eluted with PE/ EtOAc (v:v=250:1) to give 2-bromo-1-(4-octylphenyl)ethanone in a yield of 43%. Then 4-aminobenzoic acid (0.50 g, 3.65 mmol) was added to a solution of 2-bromo-1-(4-octylphenyl)ethanone (1.10 g, 3.65 mmol) in EtOH (20 mL), and the solution was stirred at room temperature overnight. The precipitated solid was obtained by filtration to give 8d as yellow solid in a yield of 20.3%.

*4-(2-(4-cyclohexylphenyl)-2-oxoeacetamido)benzoic acid (**9a**)*

A mixture of 4-aminobenzoic acid (0.50 g, 3.65 mmol), 2-(4-cyclohexylphenyl)-2-oxoacetaldehyde (0.79 g, 3.65 mmol), SeO_2 (0.41 g, 3.65 mol), and pyridine (0.60 g, 7.3 mol) in acetonitrile (20 mL) was refluxed for 4 h. The solid residue was removed by filtration. The solvent was evaporated under reduced pressure, the crude residue was purified using silica gel chromatography and was eluted with PE/ CH_3COCH_3 / CH_3COOH (v:v:v=6:1:0.04) to give 9a as brown solid in a yield of 18.6%.

*4-(2-oxo-2-(4-propylphenyl)acetamido)benzoic acid (**9b**)*

A mixture of 4-aminobenzoic acid (0.50 g, 3.65 mmol), 2-oxo-2-(4-propylphenyl)acetaldehyde (0.64 g, 3.65 mmol), SeO_2 (0.41 g, 3.65 mol), and pyridine (0.60 g, 7.3 mol) in acetonitrile (20 mL) was refluxed for 4 h. The solid residue was removed by filtration. The solvent was evaporated under reduced pressure, the crude residue was purified using silica gel chromatography and was eluted with PE/ CH_3COCH_3 / CH_3COOH (v:v:v=5:1:0.04) to give 9b as yellow solid in a yield of 17%.

*4-(2-(4-hexylphenyl)-2-oxoacetamido)benzoic acid (**9c**)*

A mixture of 4-aminobenzoic acid (0.50 g, 3.65 mmol), 2-(4-hexylphenyl)-2-oxoacetaldehyde (0.80 g, 3.65 mmol), SeO_2 (0.41 g, 3.65 mol), and pyridine (0.60 g, 7.3 mol) in acetonitrile (20 mL) was refluxed for 4 h. The solid residue was removed by filtration. The solvent was evaporated under reduced pressure, the crude residue was purified using silica gel chromatography and was eluted with PE/ CH_3COCH_3/ CH_3COOH (v:v:v=6:1:0.05) to give 9c as yellow solid in a yield of 15%.

*4-(2-(4-octylphenyl)-2-oxoacetamido)benzoic acid (**9d**)*

A mixture of 4-aminobenzoic acid (0.50 g, 3.65 mmol), 2-(4-octylphenyl)-2-oxoacetaldehyde (0.90 g, 3.65 mmol), SeO_2 (0.41 g, 3.65 mol), and pyridine (0.60 g, 7.3 mol) in acetonitrile (20 mL) was refluxed for 4 h. The solid residue was removed by filtration. The solvent was evaporated under reduced pressure, the crude residue was purified using silica gel chromatography and was eluted with PE/ CH_3COCH_3 / CH_3COOH (v:v:v=6:1:0.05) to give 9d as yellow solid in a yield of 14.6%.

Fig S1. ^{1}H NMR and ^{13}C NMR spectra of **6a**.

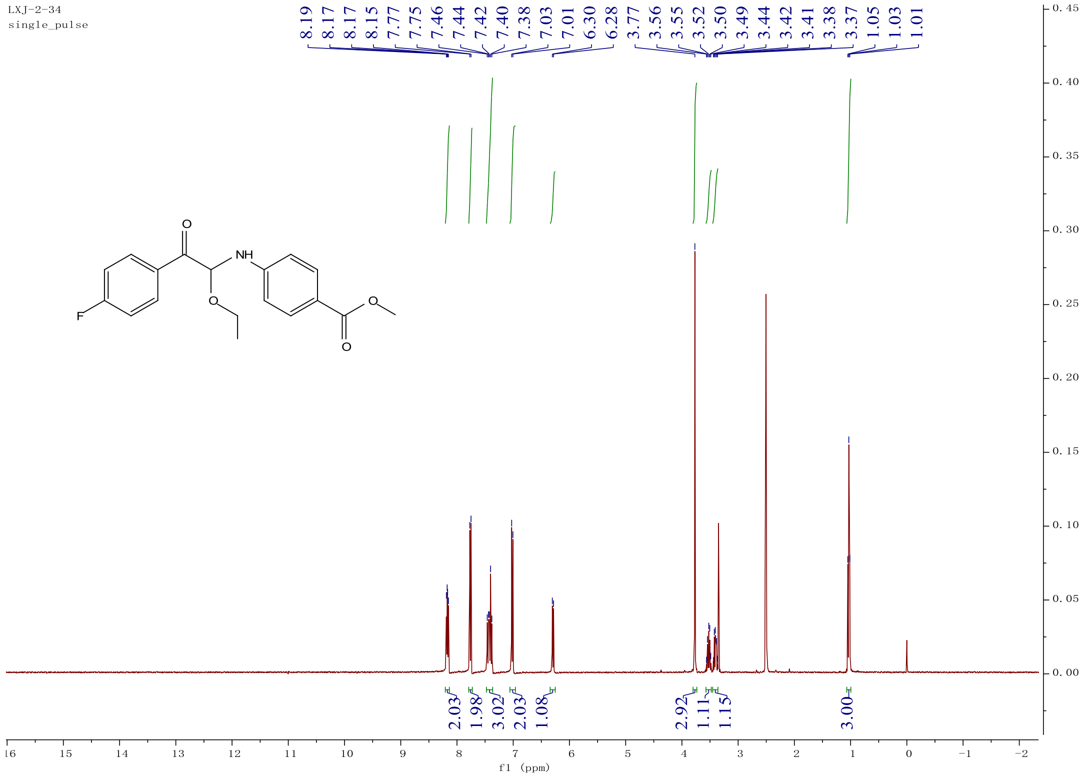

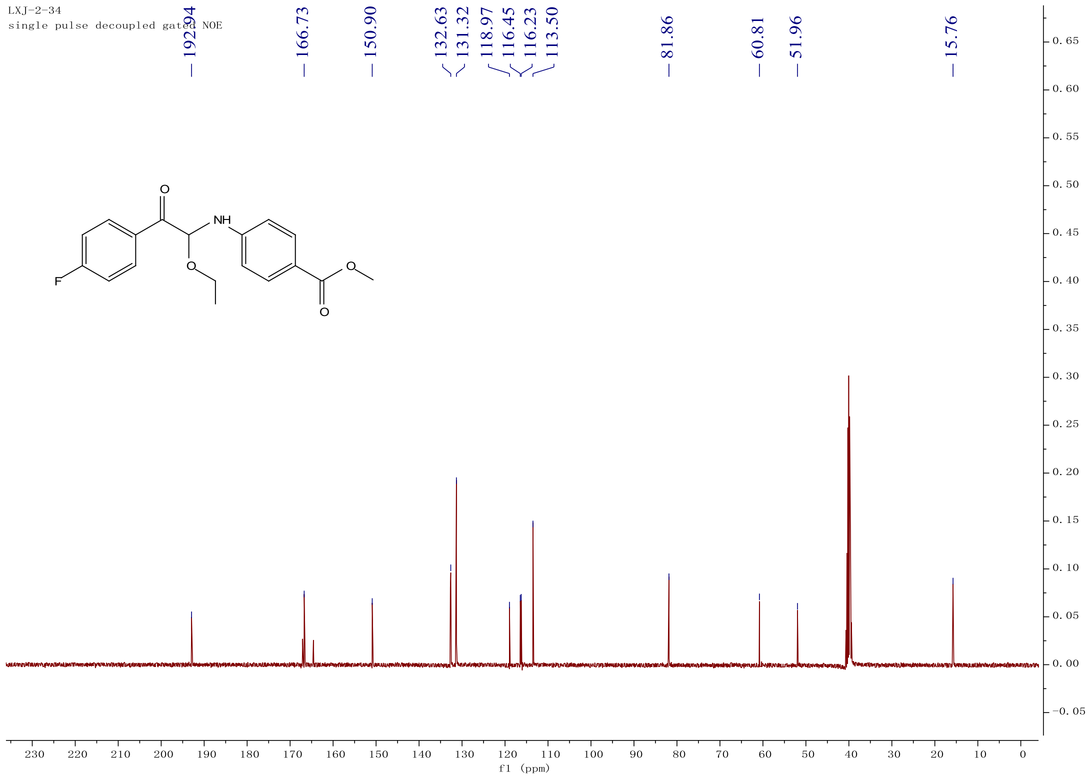

Fig S2. ^{1}H NMR and ^{13}C NMR spectra of **6b**.

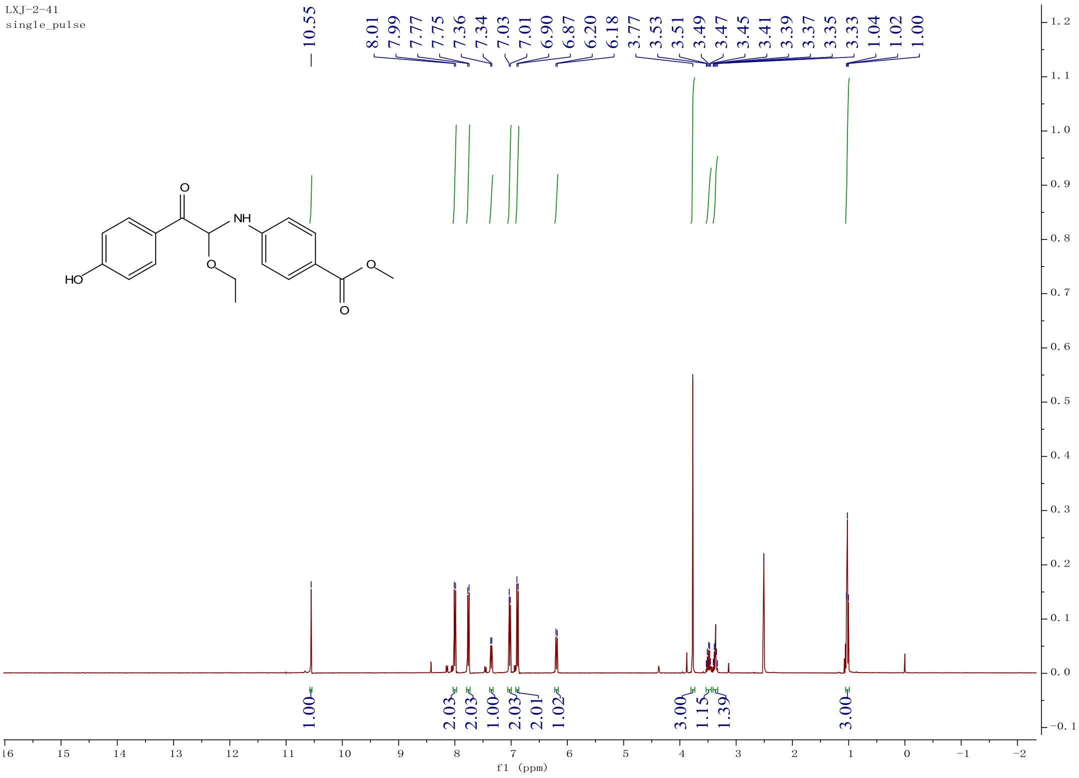

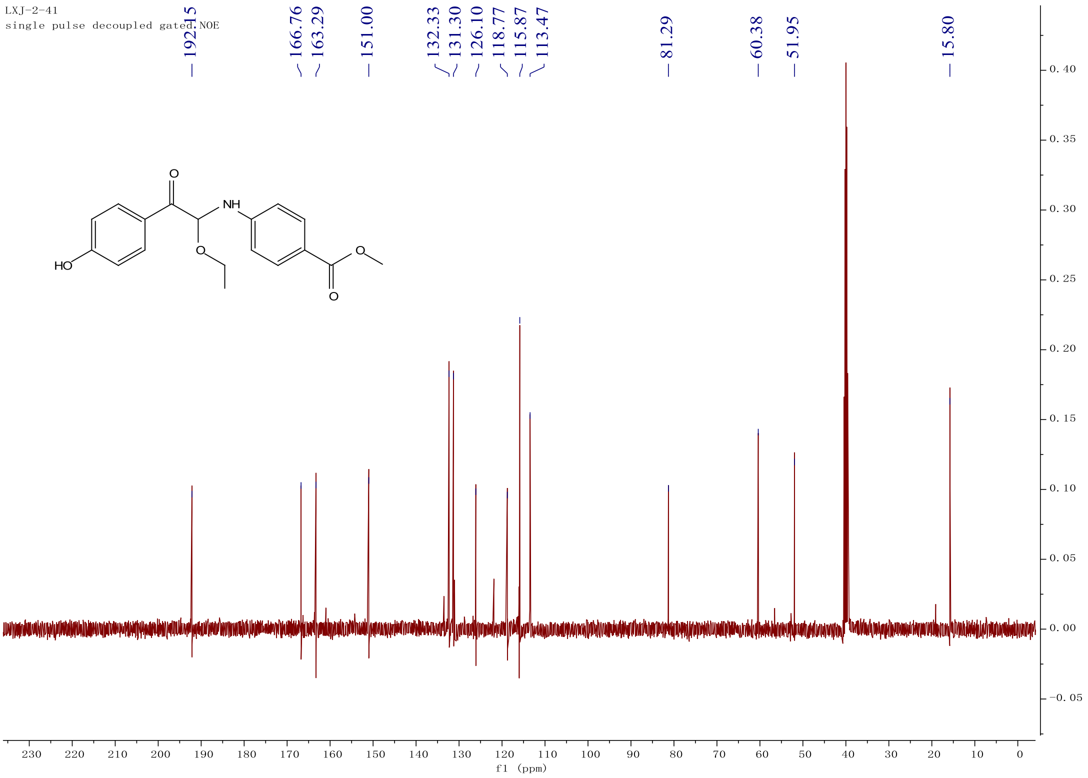

Fig S3. ^{1}H NMR and ^{13}C NMR spectra of **6c**.

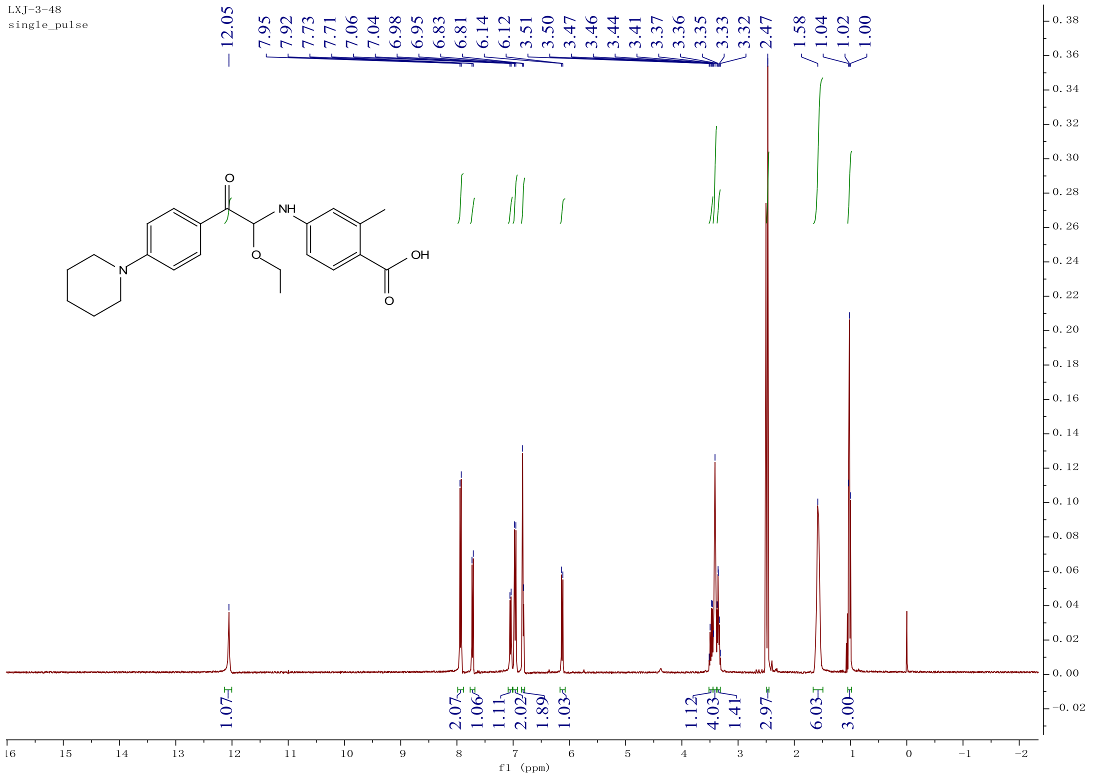

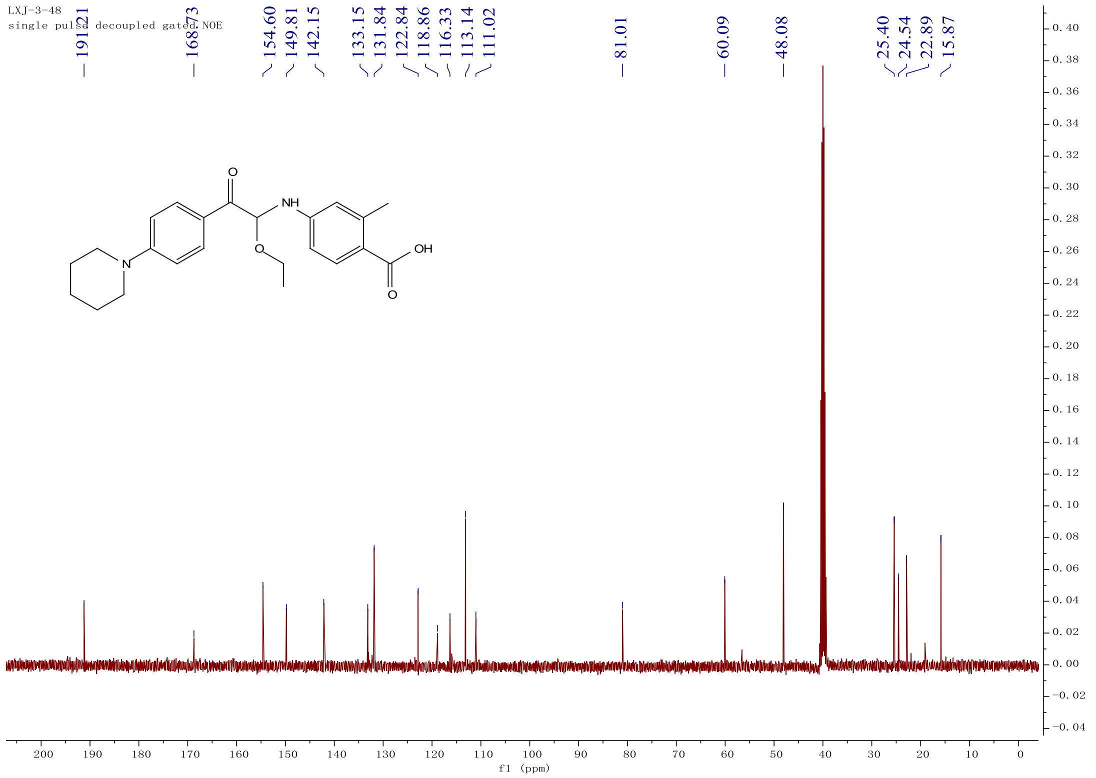

Fig S4. ^{1}H NMR and ^{13}C NMR spectra of **6d**.

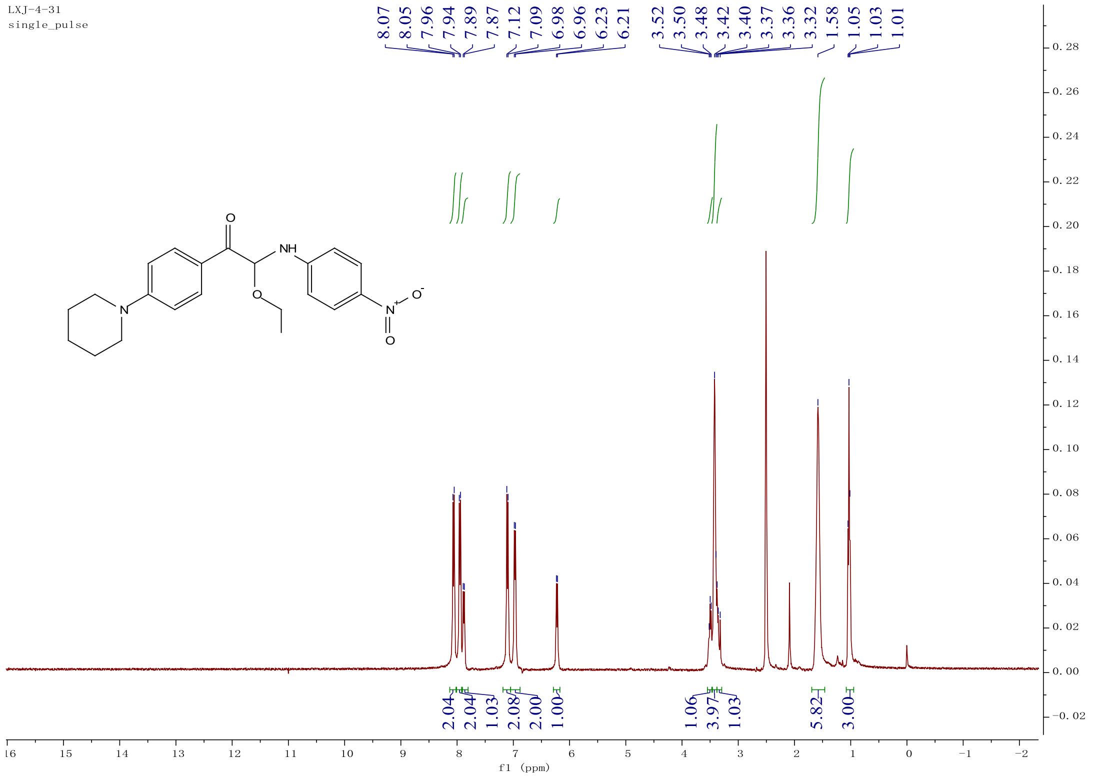

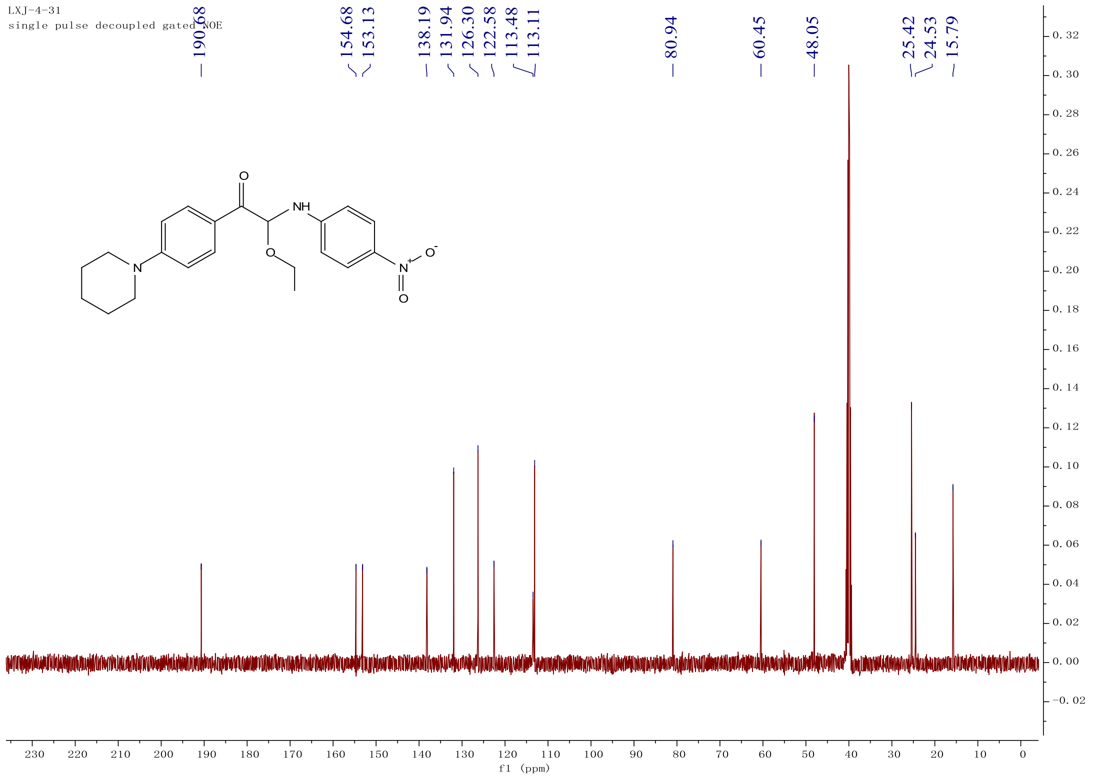

Fig S5. ^{1}H NMR and ^{13}C NMR spectra of **6e**.

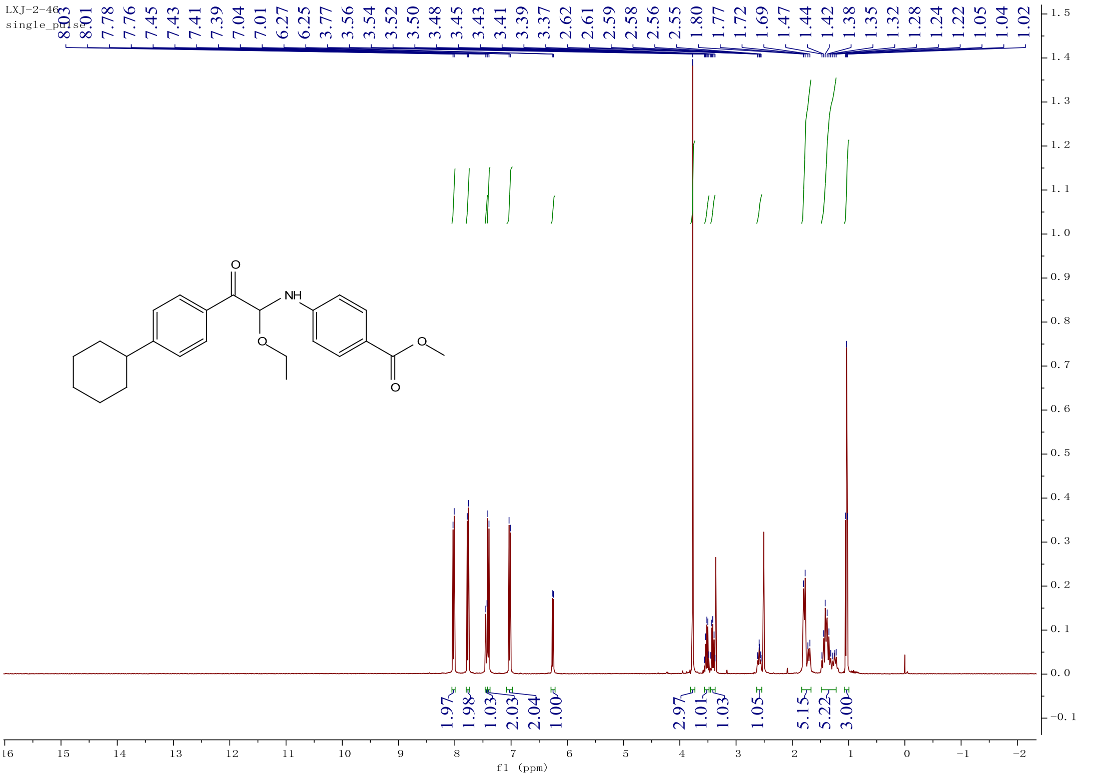

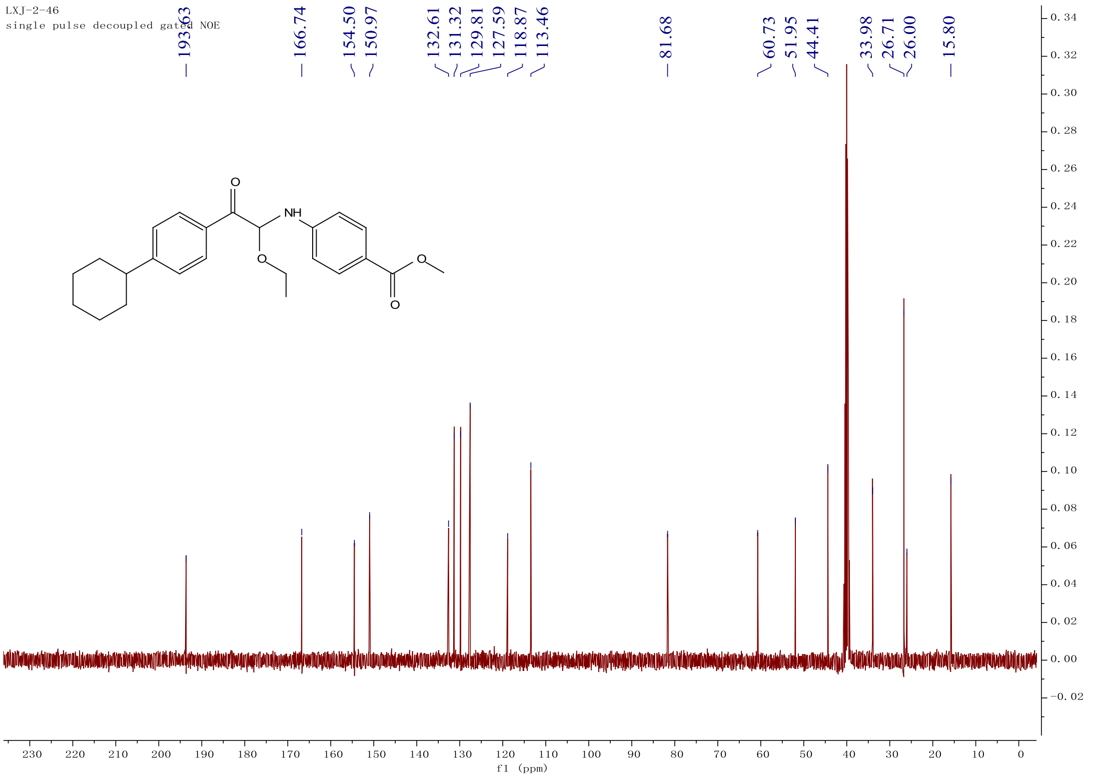

Fig S6. ^{1}H NMR and ^{13}C NMR spectra of **6f**.

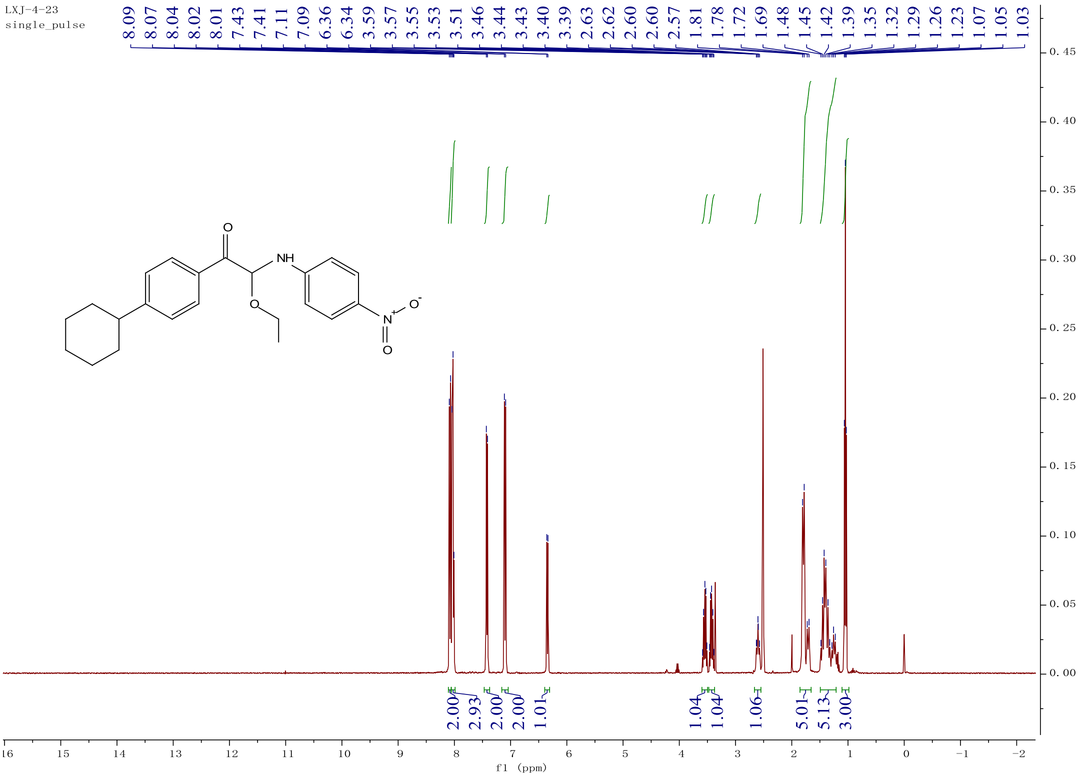

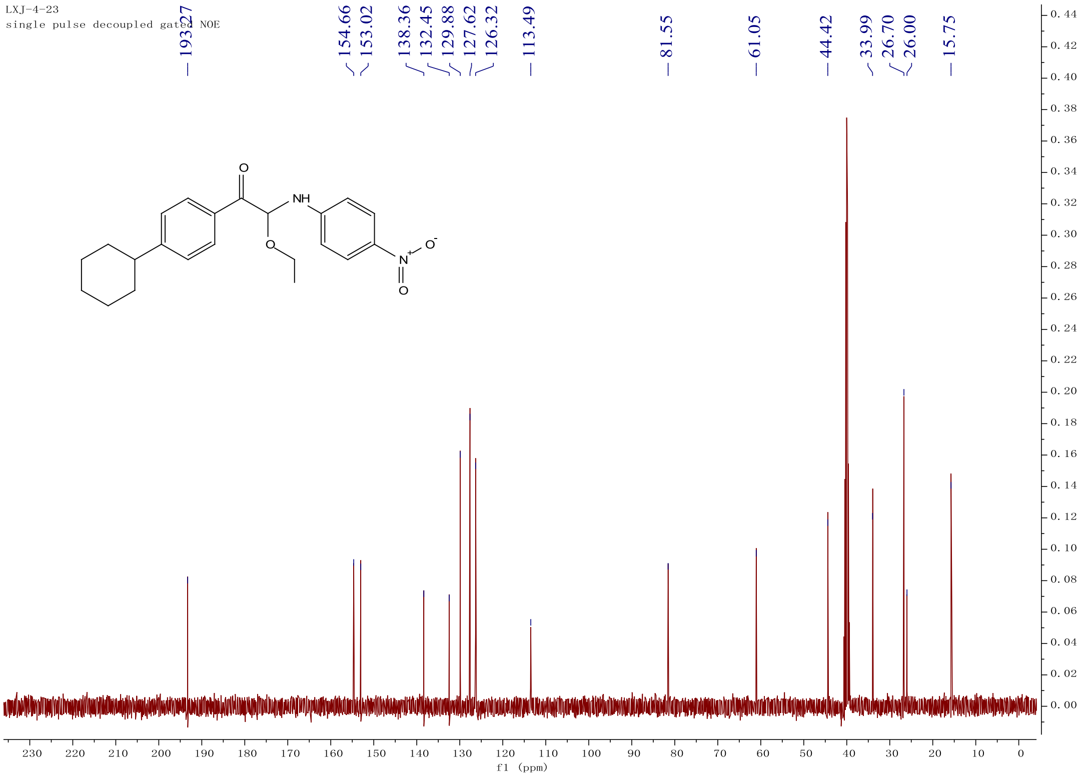

Fig S7. ^{1}H NMR and ^{13}C NMR spectra of **6g**.

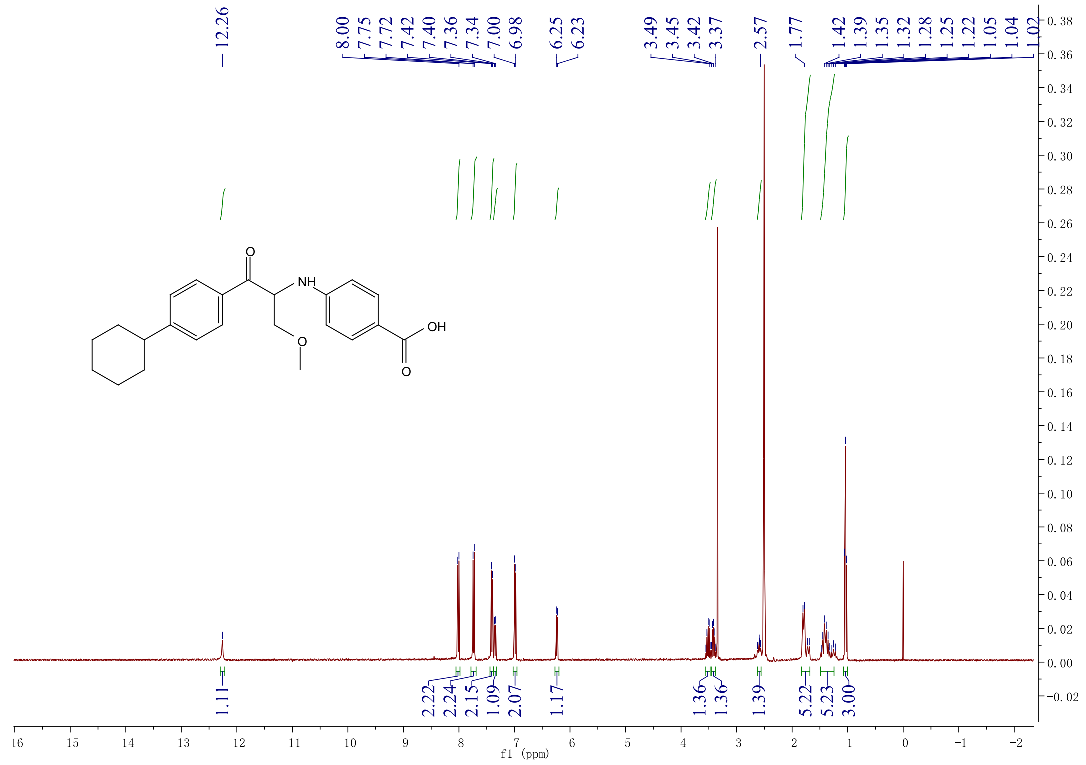

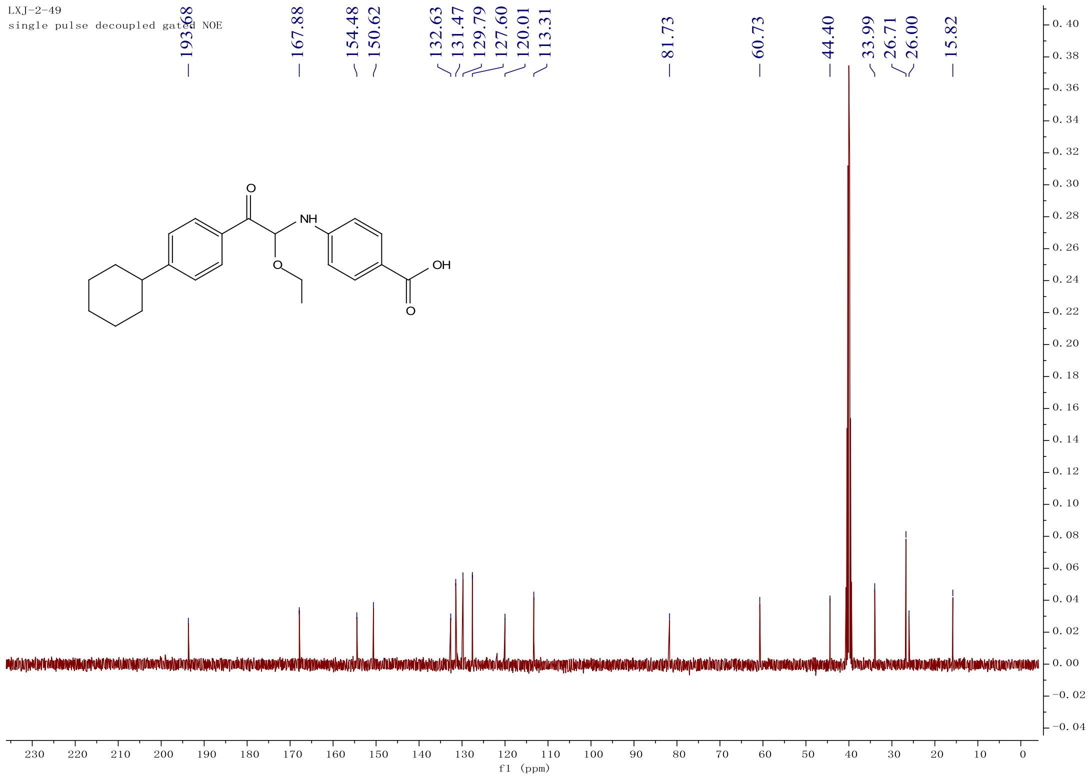

Fig S8. ^{1}H NMR and ^{13}C NMR spectra of **6h**.

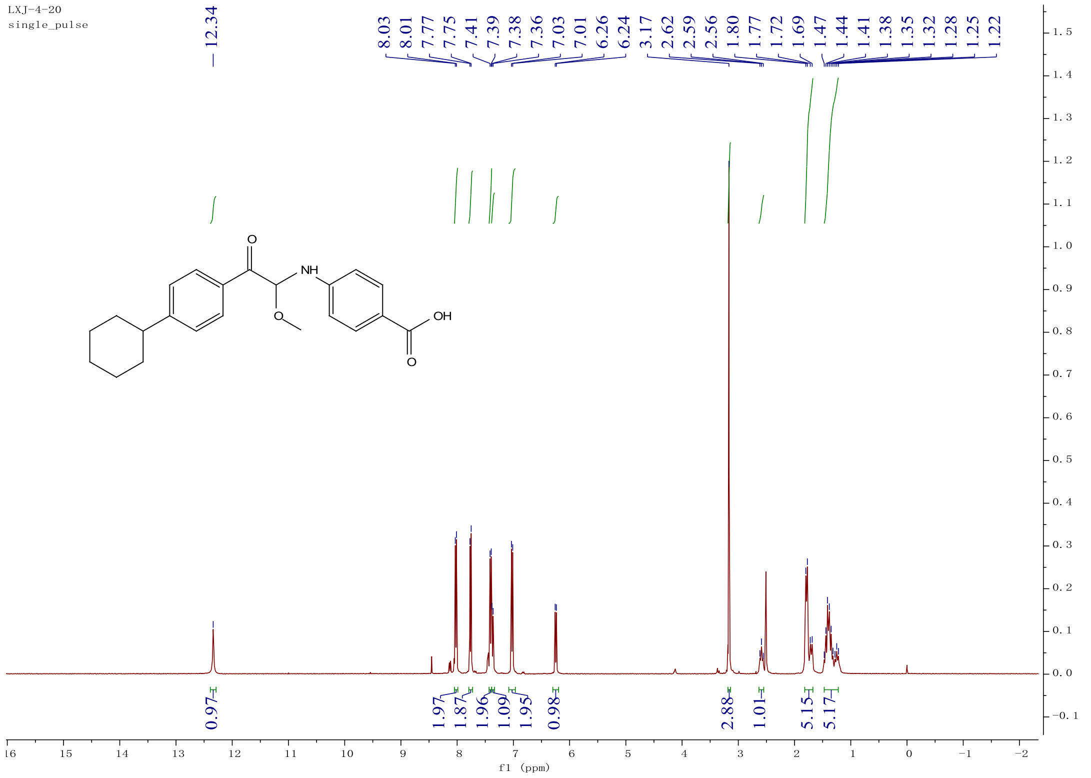

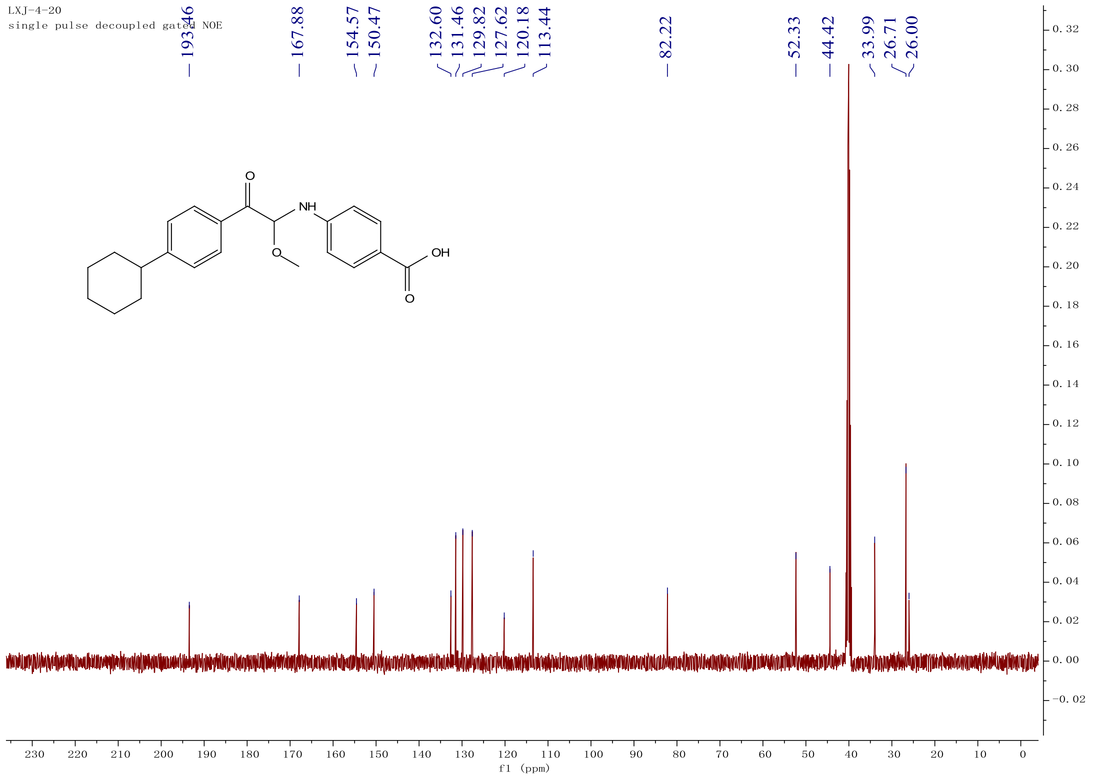

Fig S9. ^{1}H NMR and ^{13}C NMR spectra of **6i**.

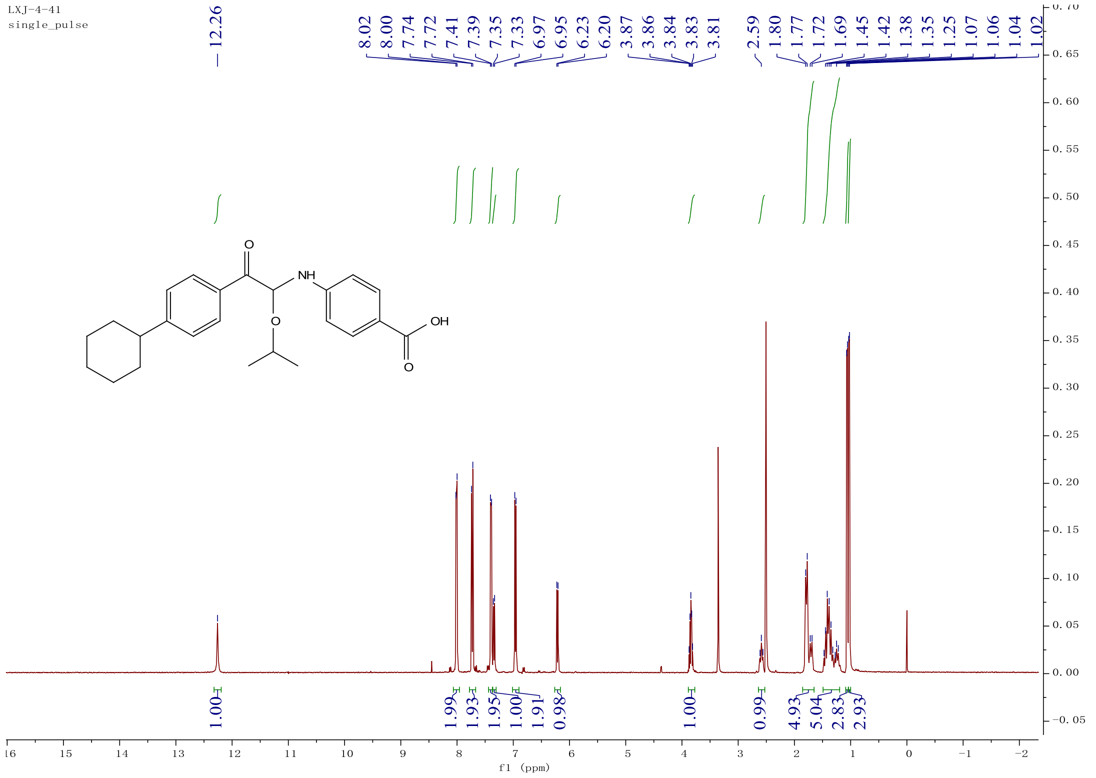

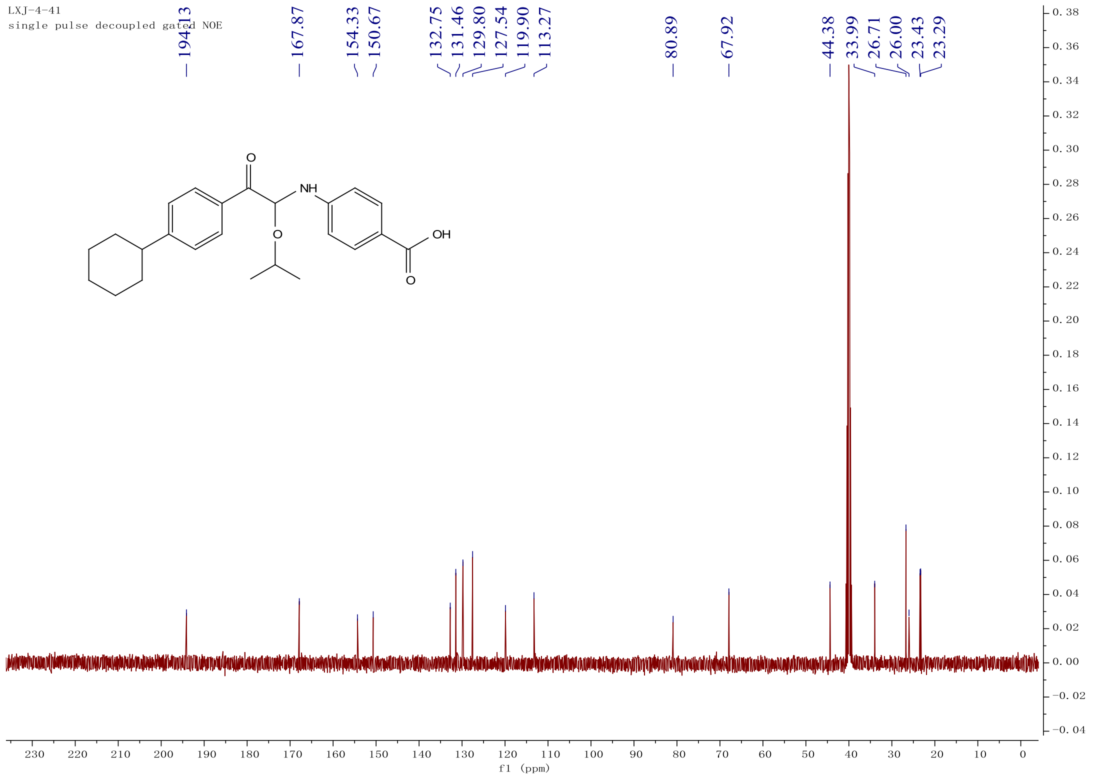

Fig S10. ^{1}H NMR and ^{13}C NMR spectra of **6j**.

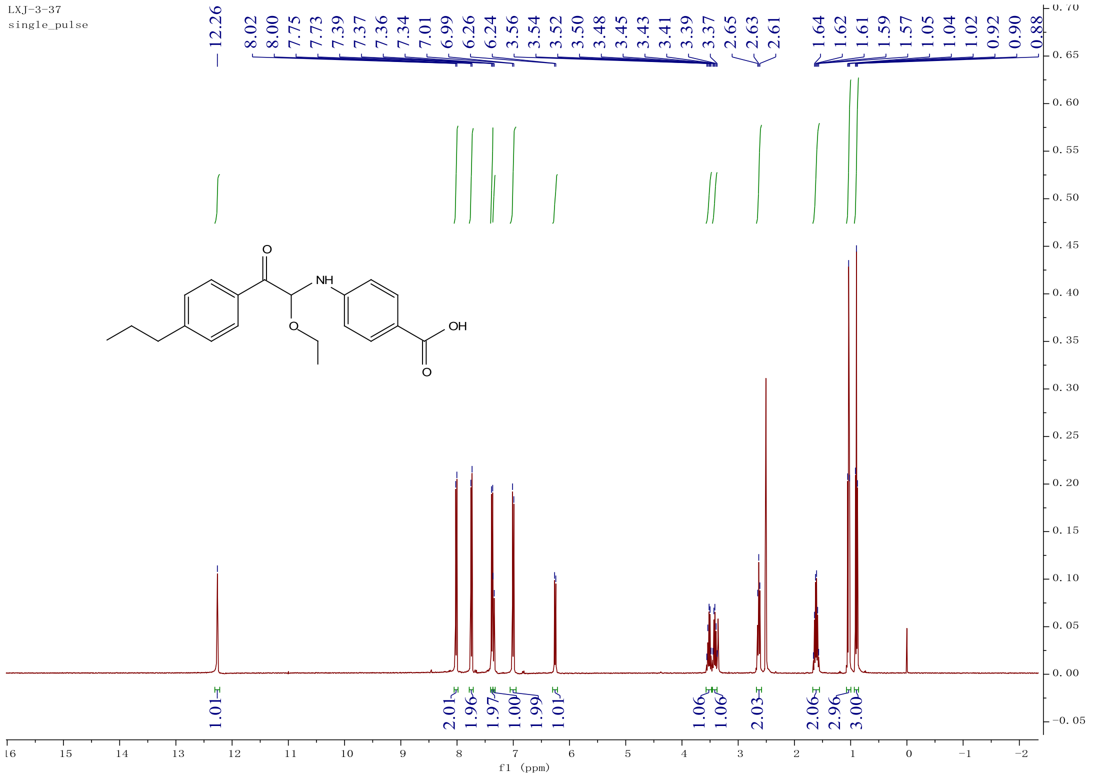

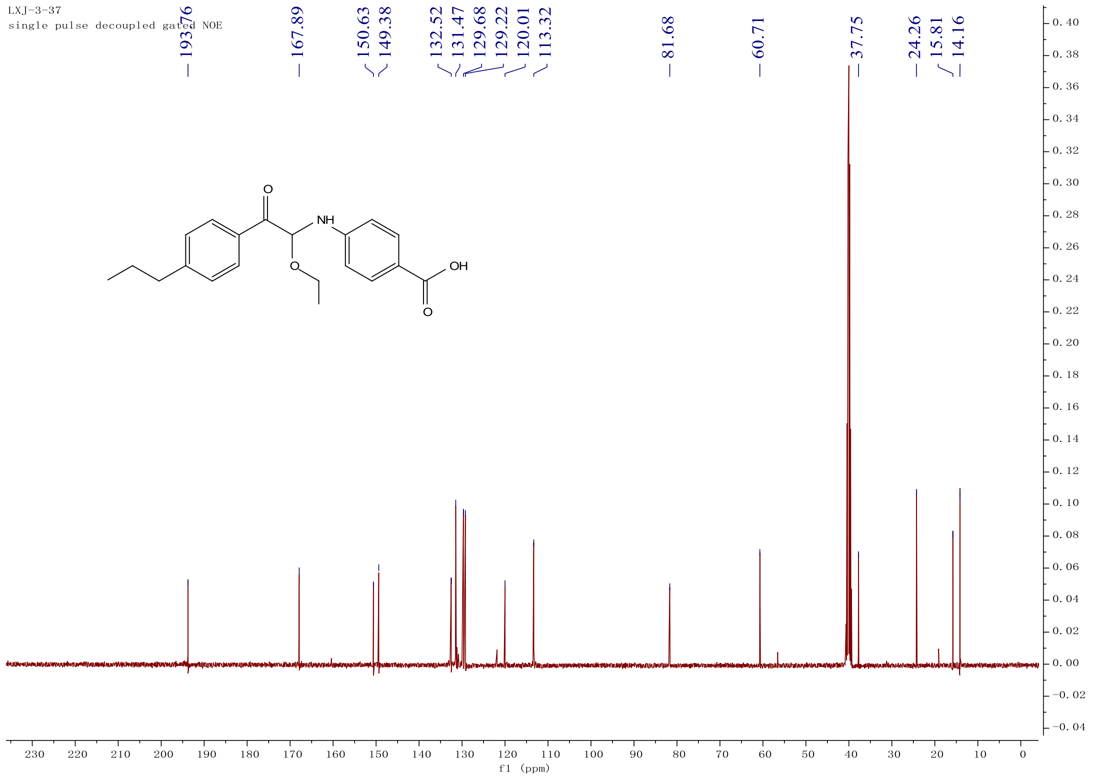

Fig S11. ^{1}H NMR and ^{13}C NMR spectra of **6k**.

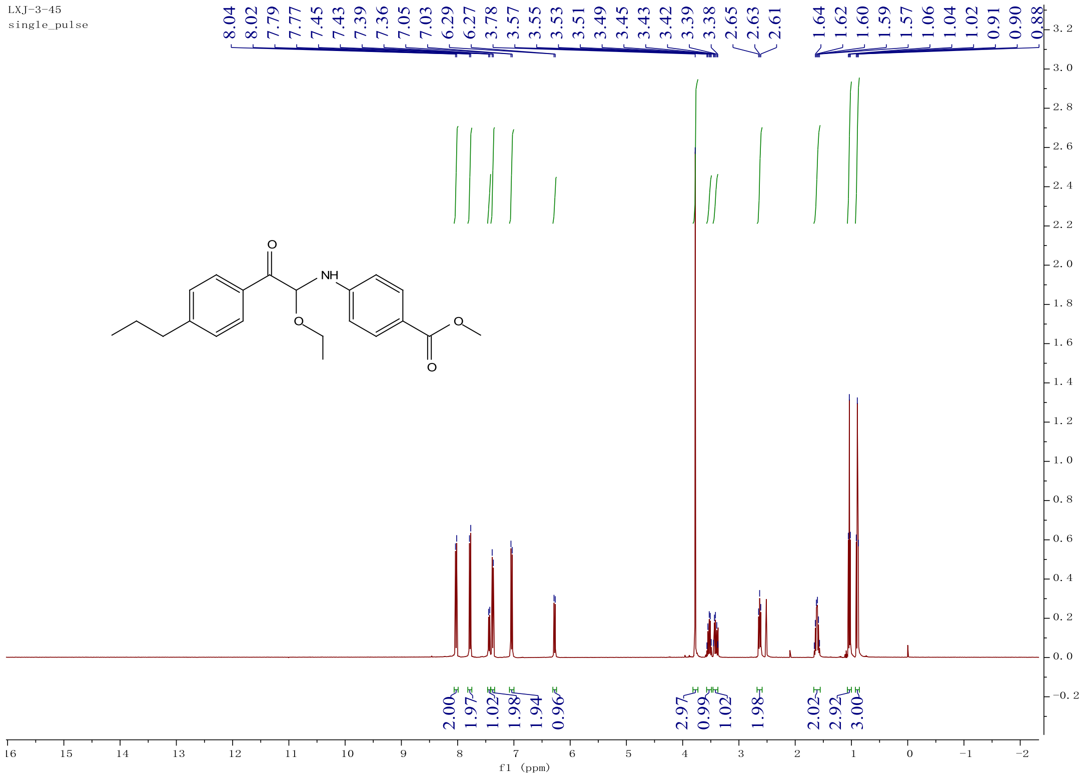

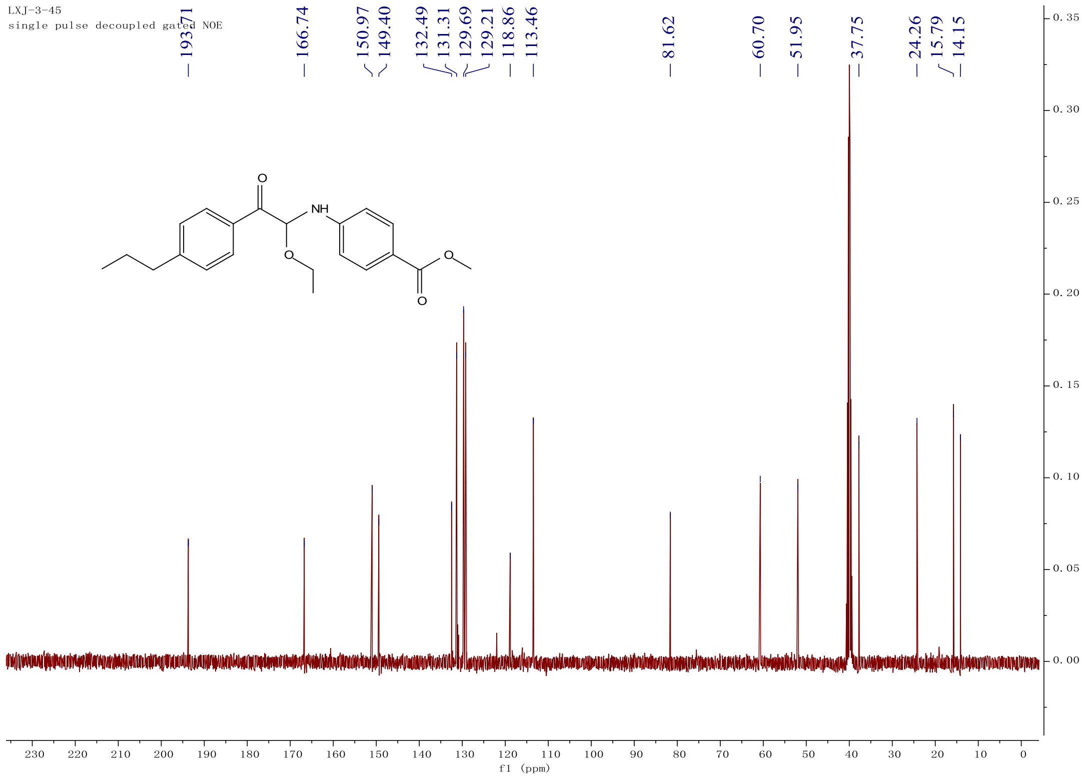

Fig S12. ^{1}H NMR and ^{13}C NMR spectra of **6l**.

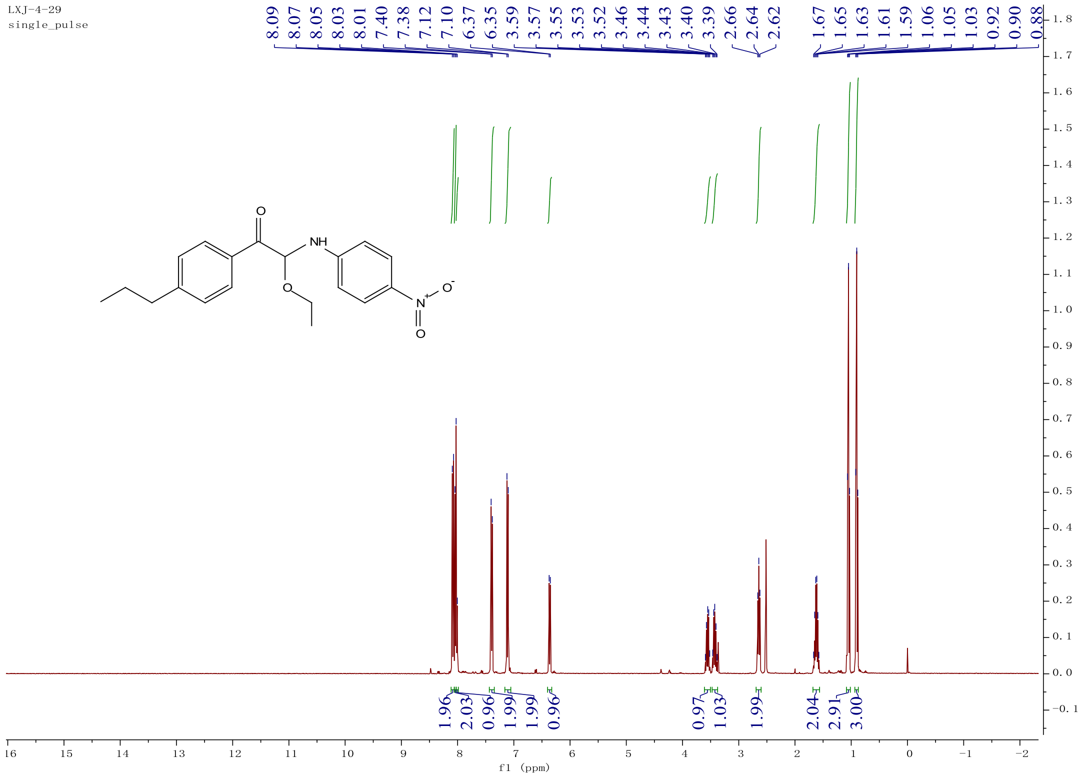

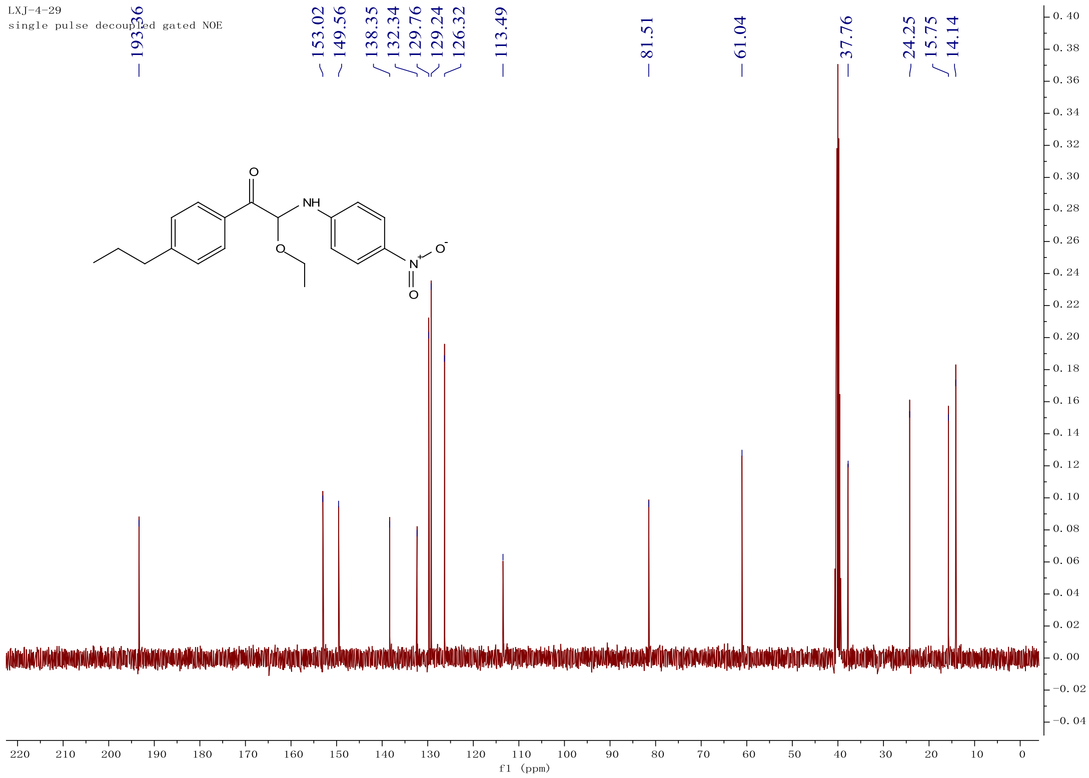

Fig S13. ^{1}H NMR and ^{13}C NMR spectra of **6m**.

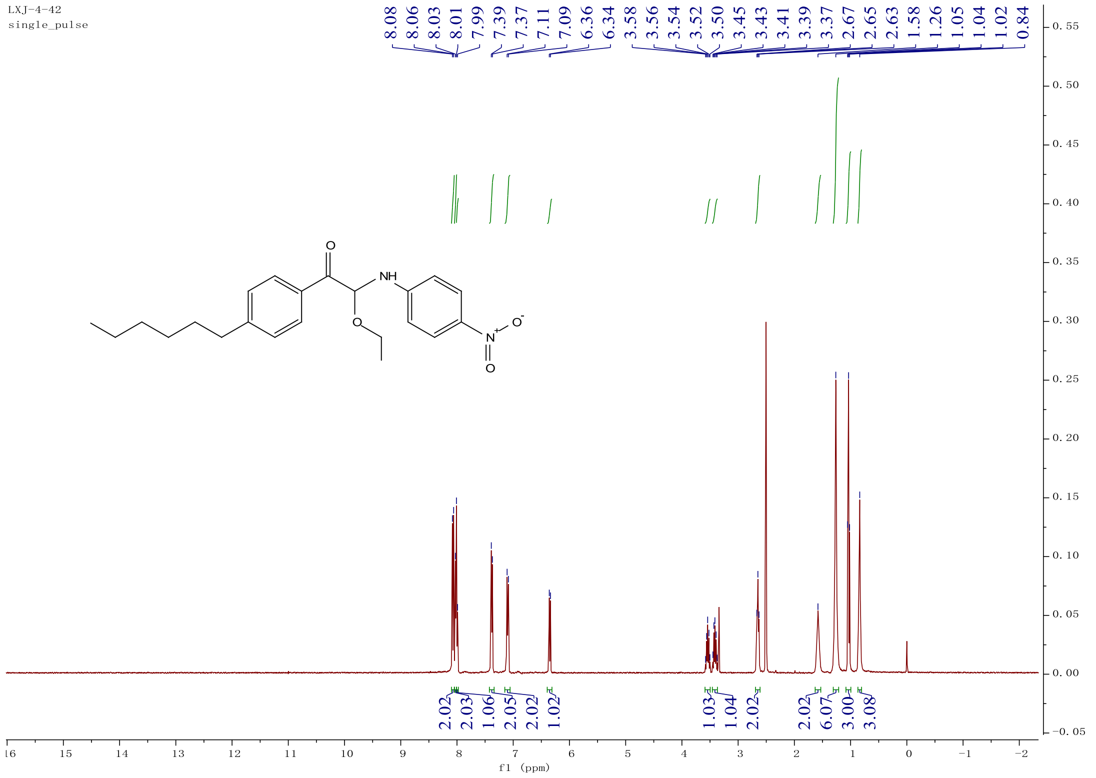

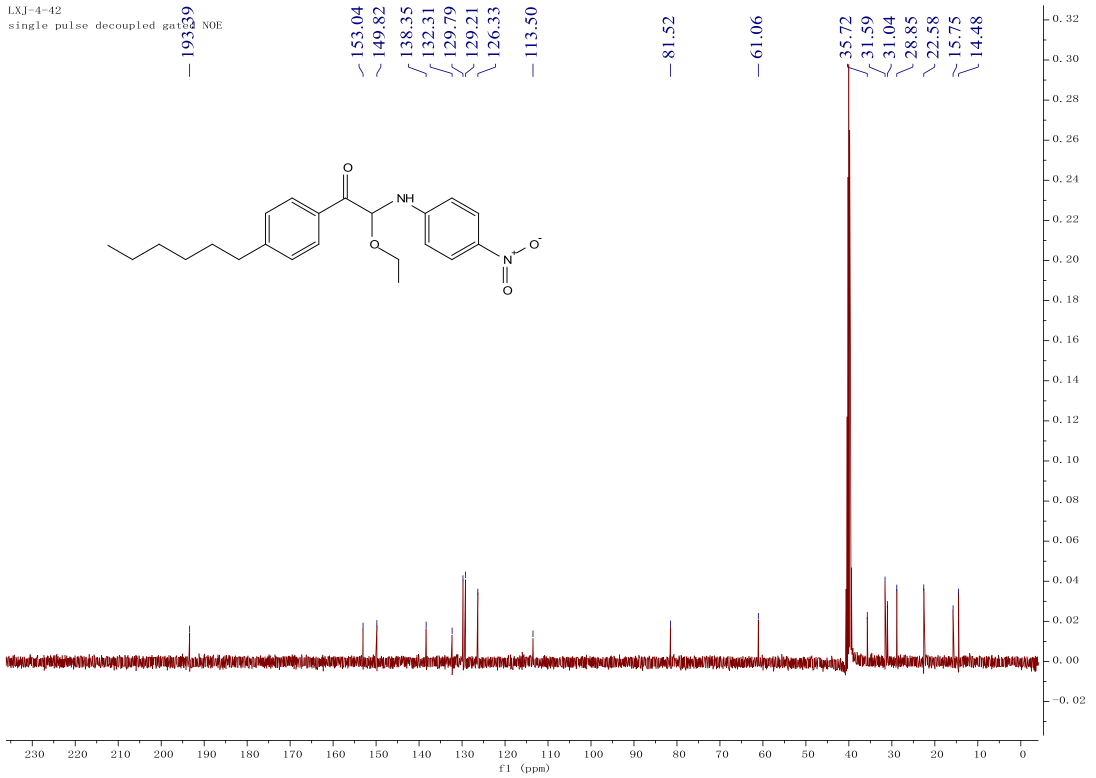

Fig S14. ^{1}H NMR and ^{13}C NMR spectra of **6n**.

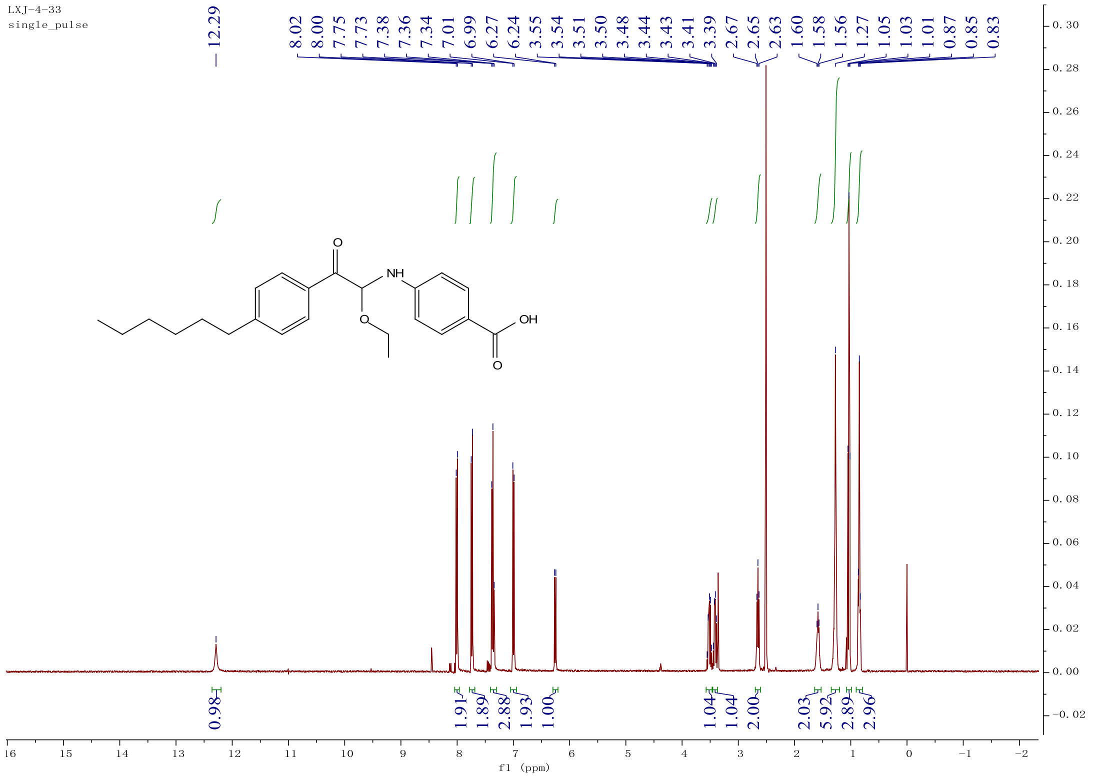

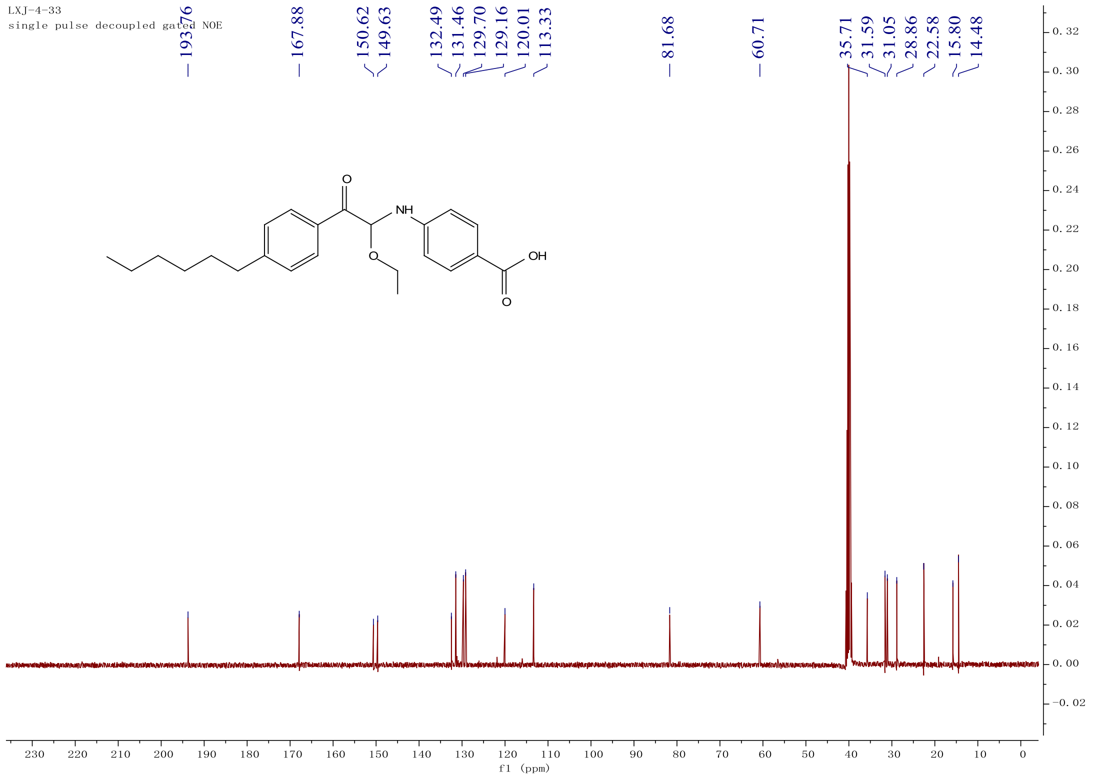

Fig S15. ^{1}H NMR and ^{13}C NMR spectra of **6o**.

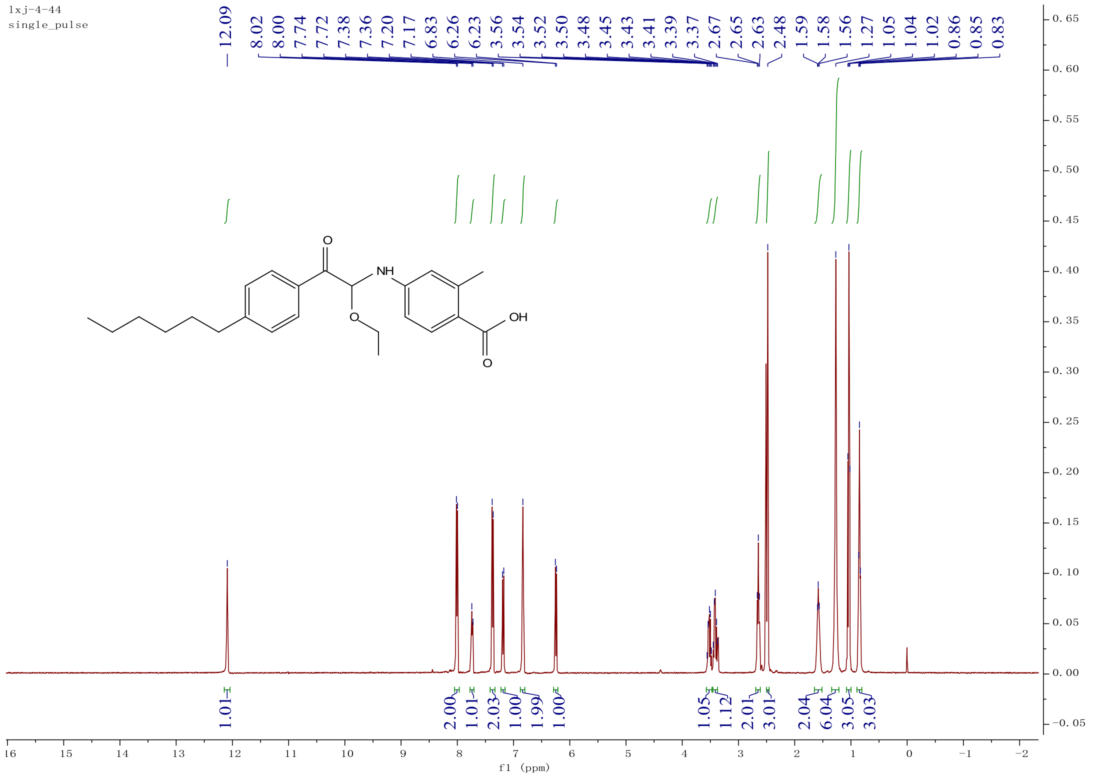

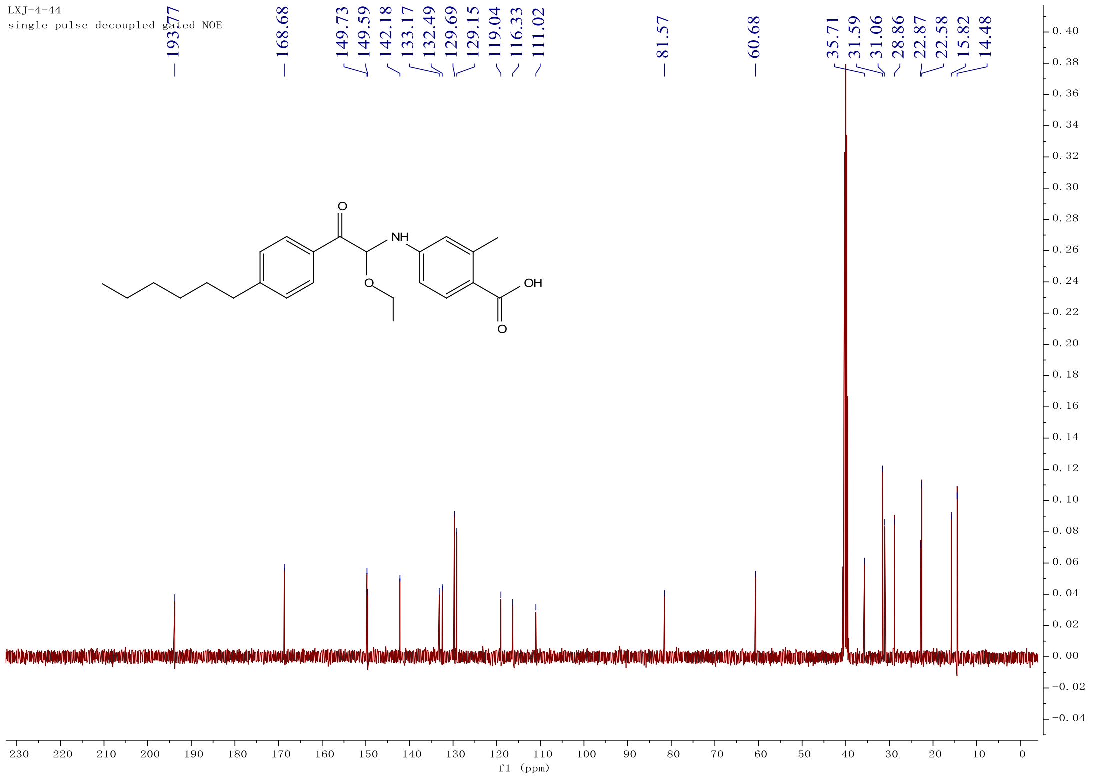

Fig S16. ^{1}H NMR and ^{13}C NMR spectra of **6p**.

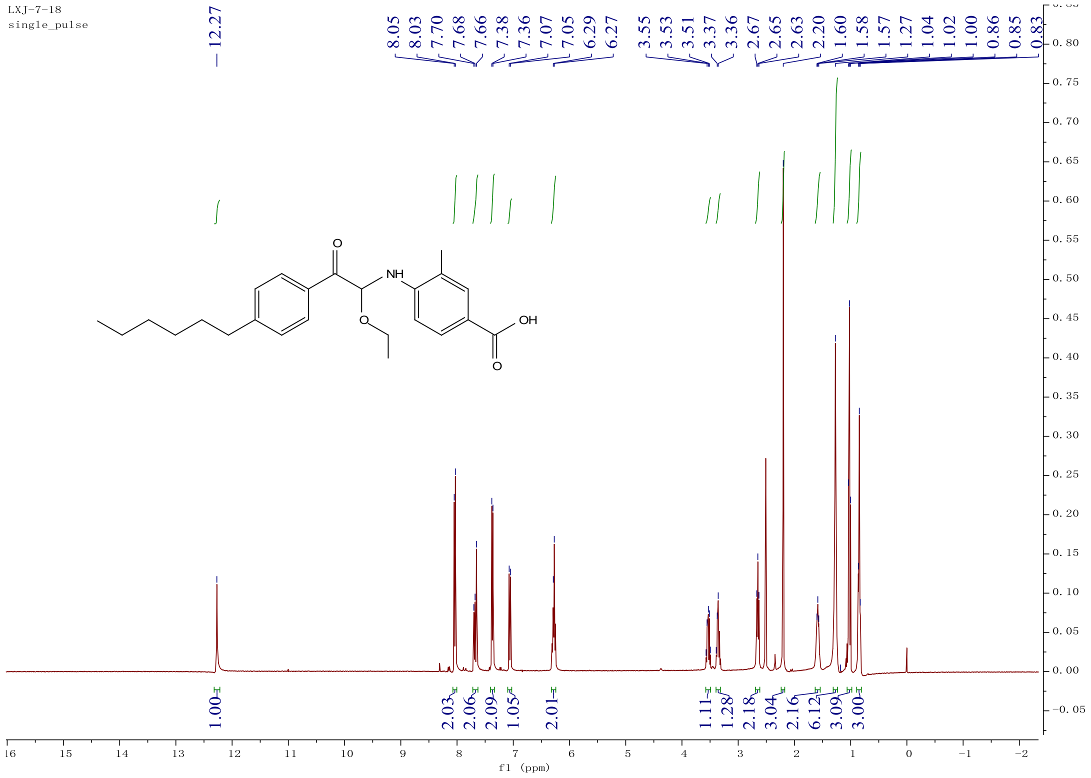

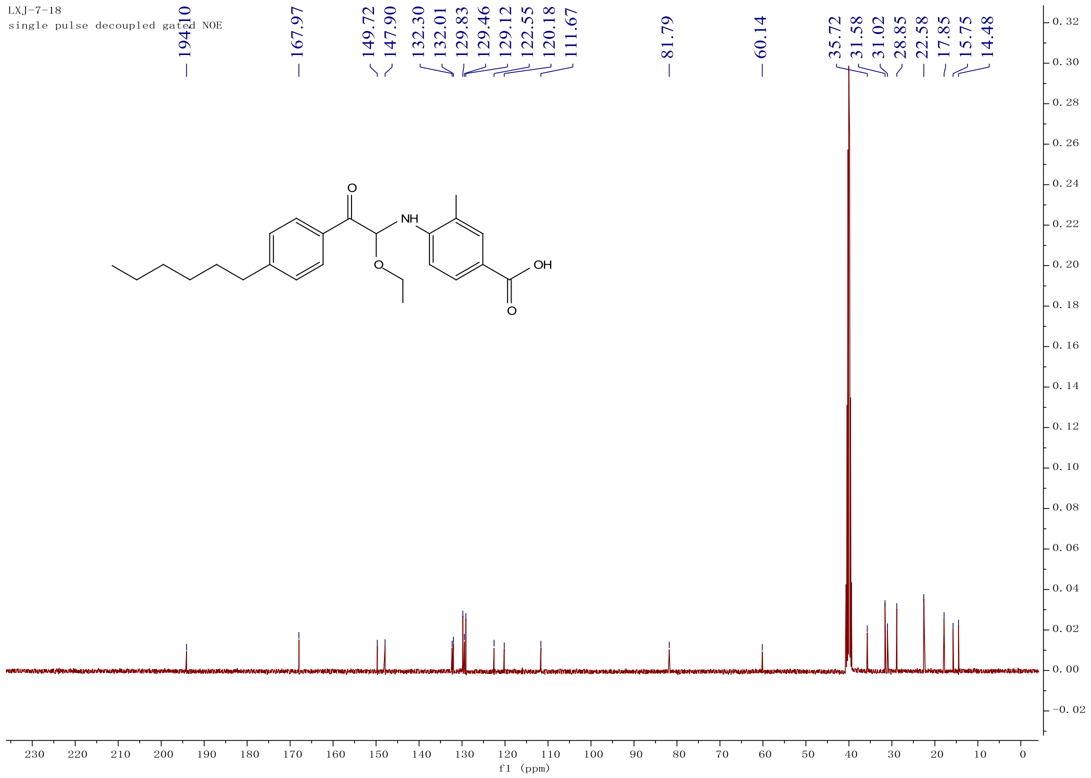

Fig S17. ^{1}H NMR and ^{13}C NMR spectra of **6q**.

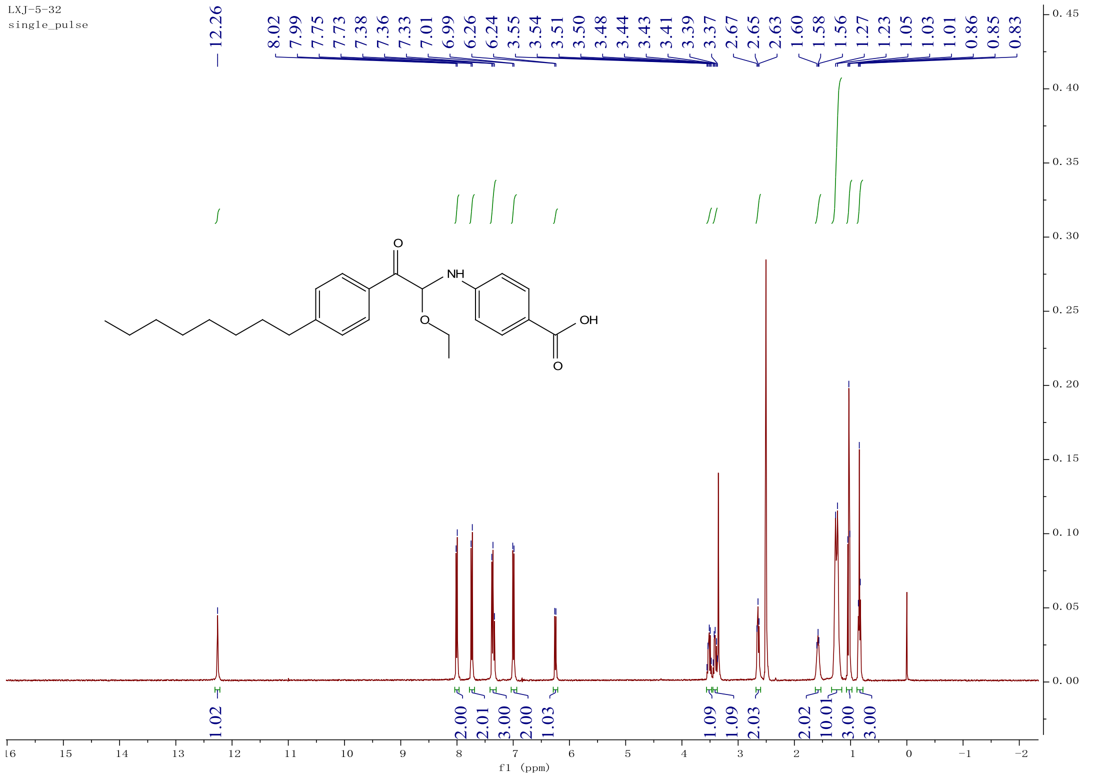

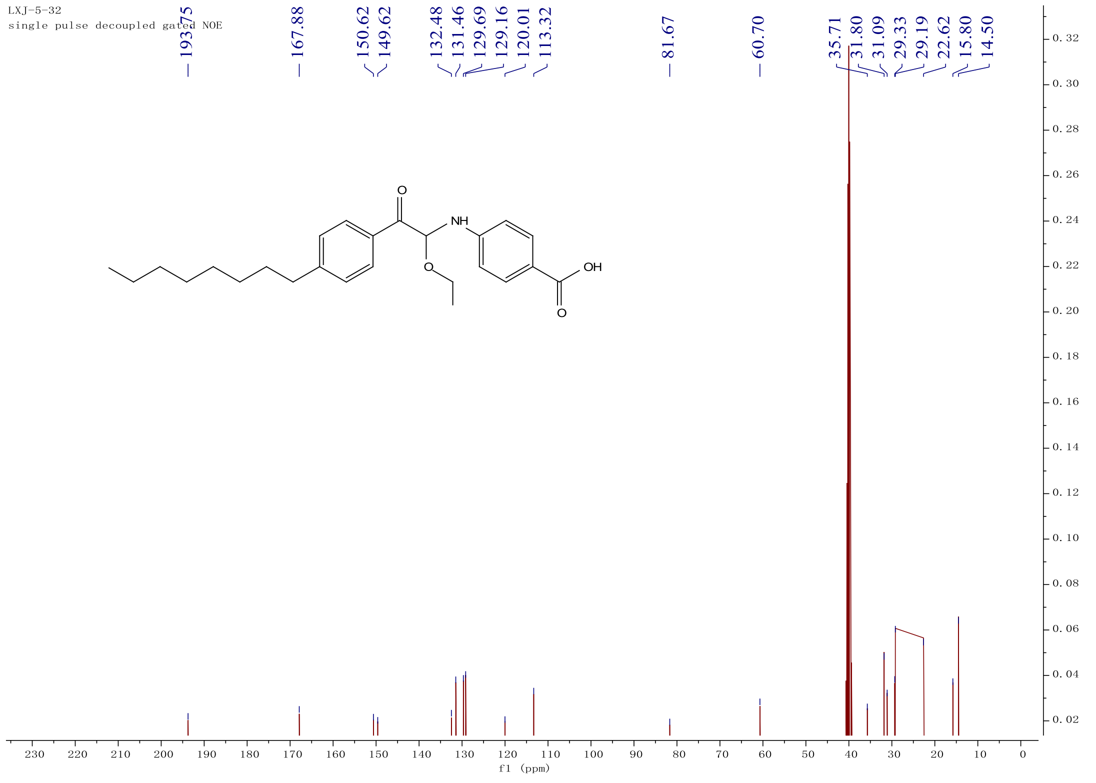

Fig S18. ^{1}H NMR and ^{13}C NMR spectra of **6r**.

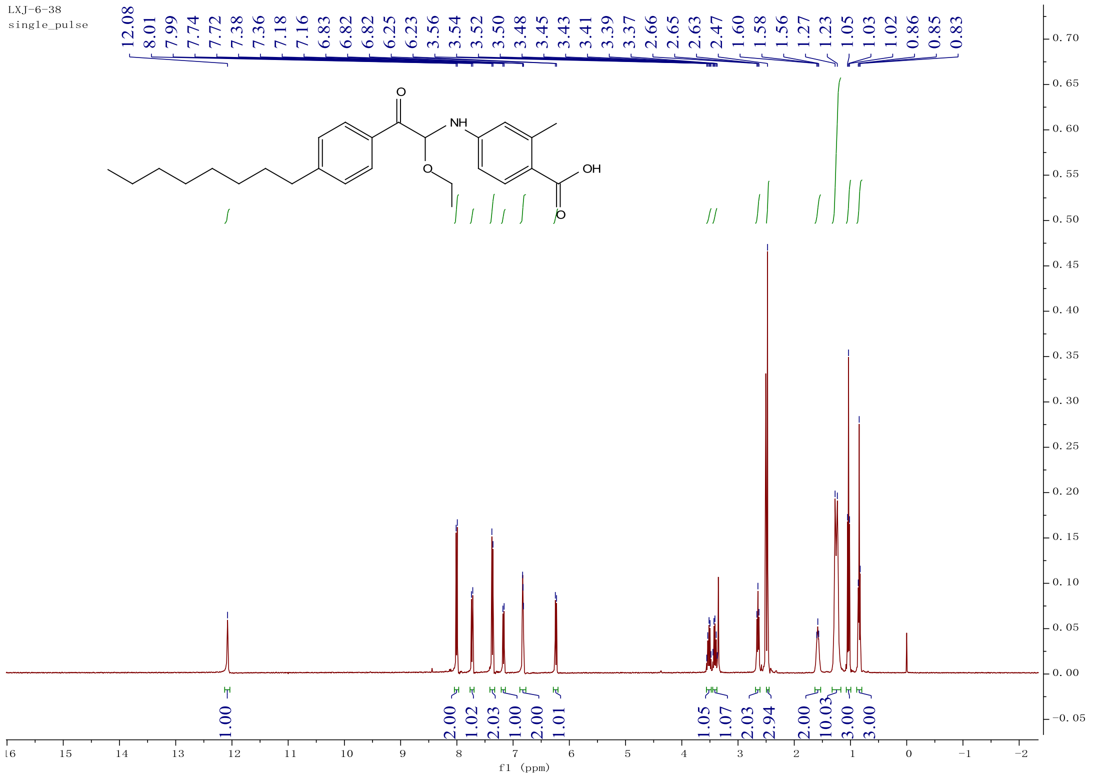

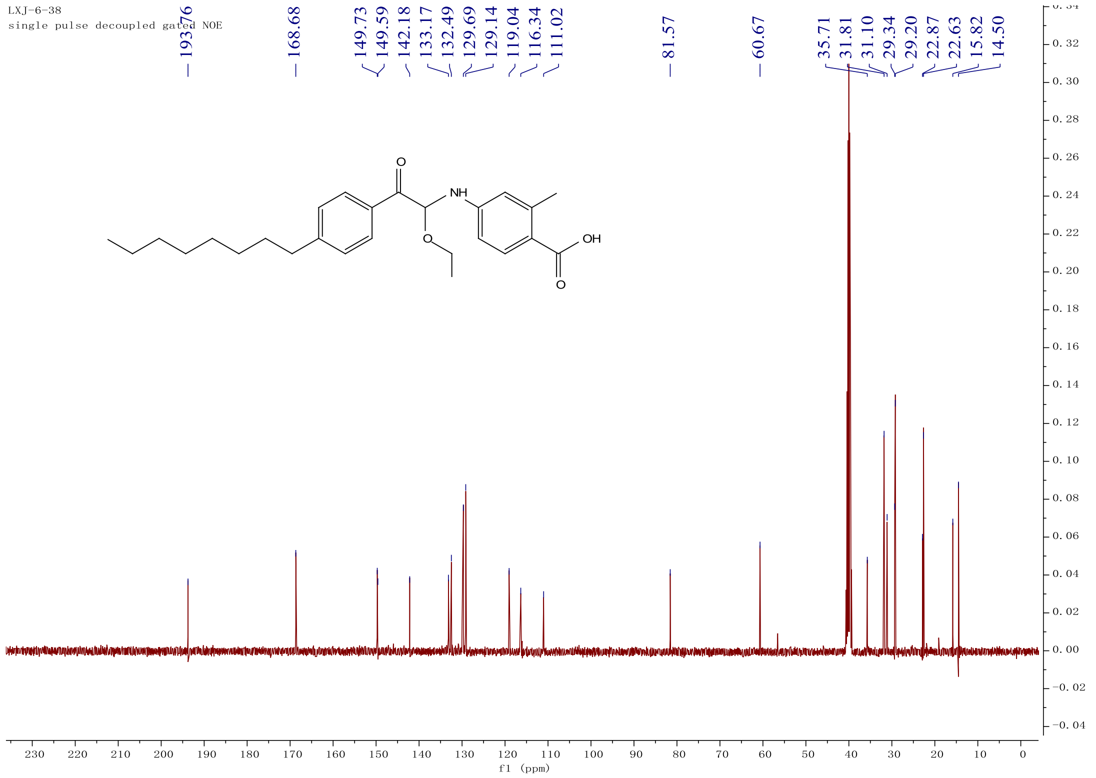

Fig S19. ^{1}H NMR and ^{13}C NMR spectra of **8a**.

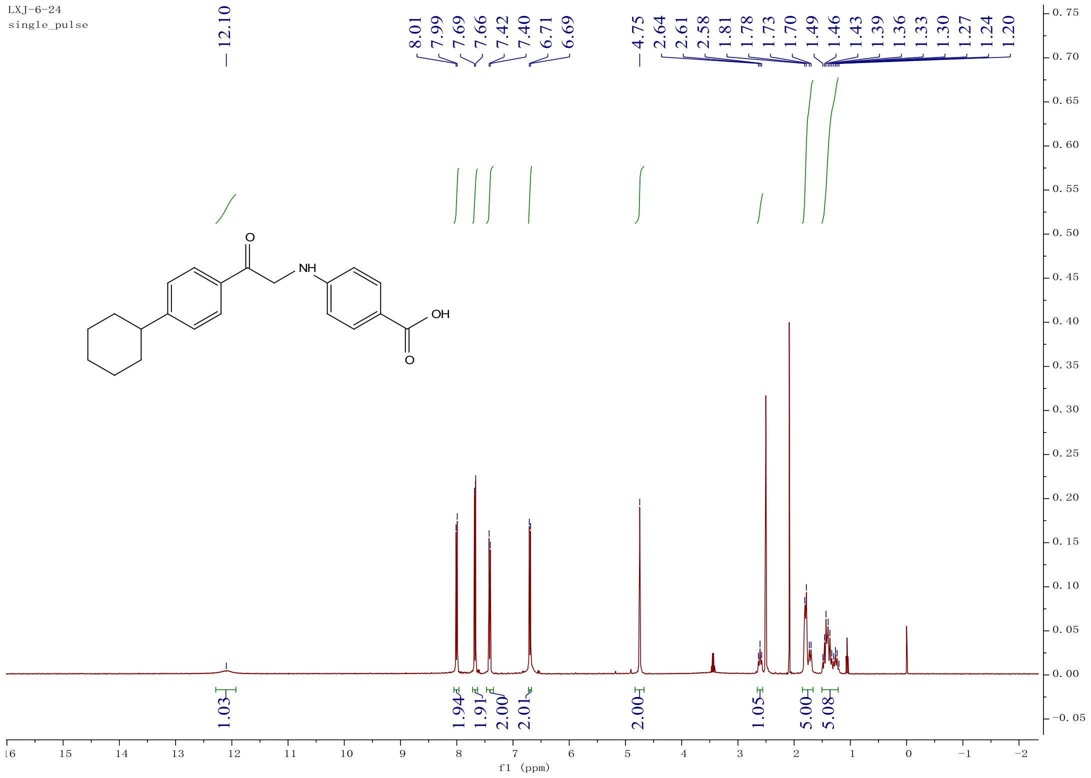

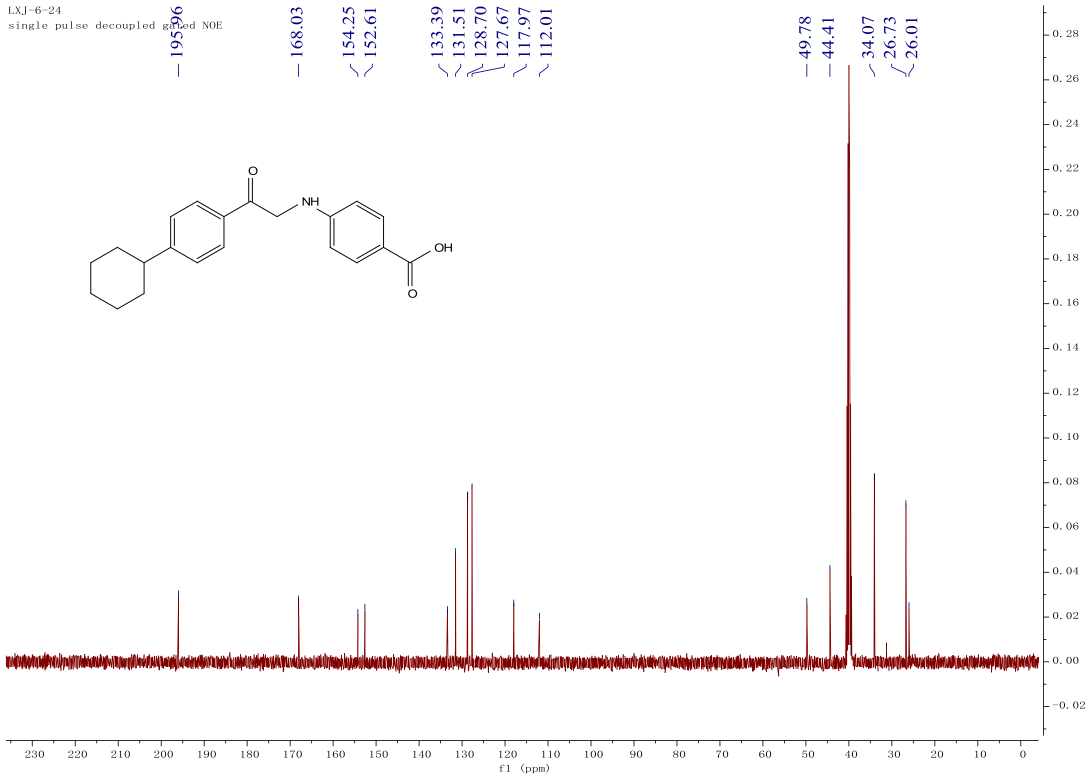

Fig S20. ^{1}H NMR and ^{13}C NMR spectra of **8b**.

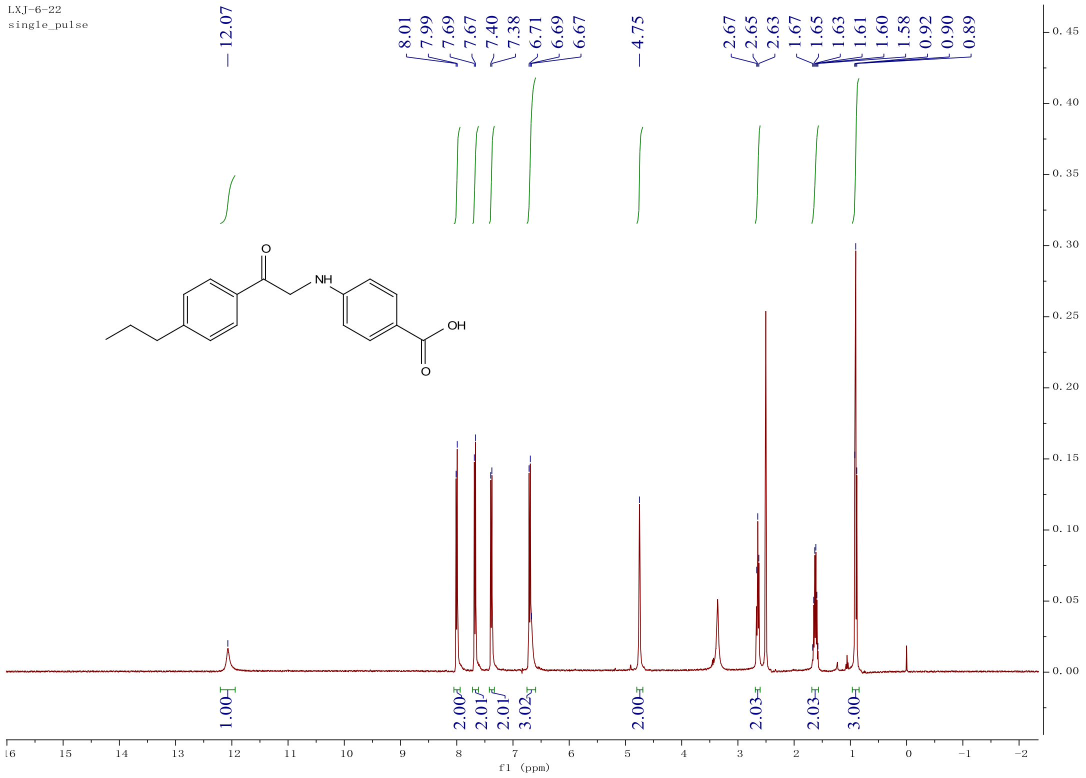

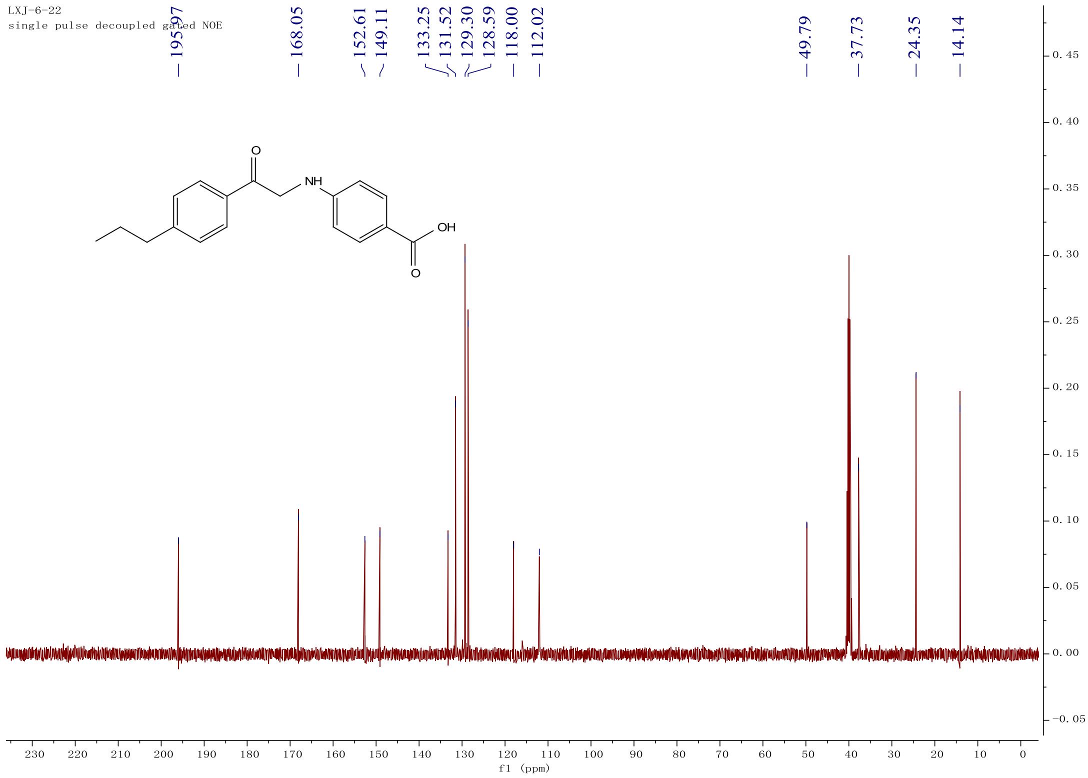

Fig S21. ^{1}H NMR and ^{13}C NMR spectra of **8c**.

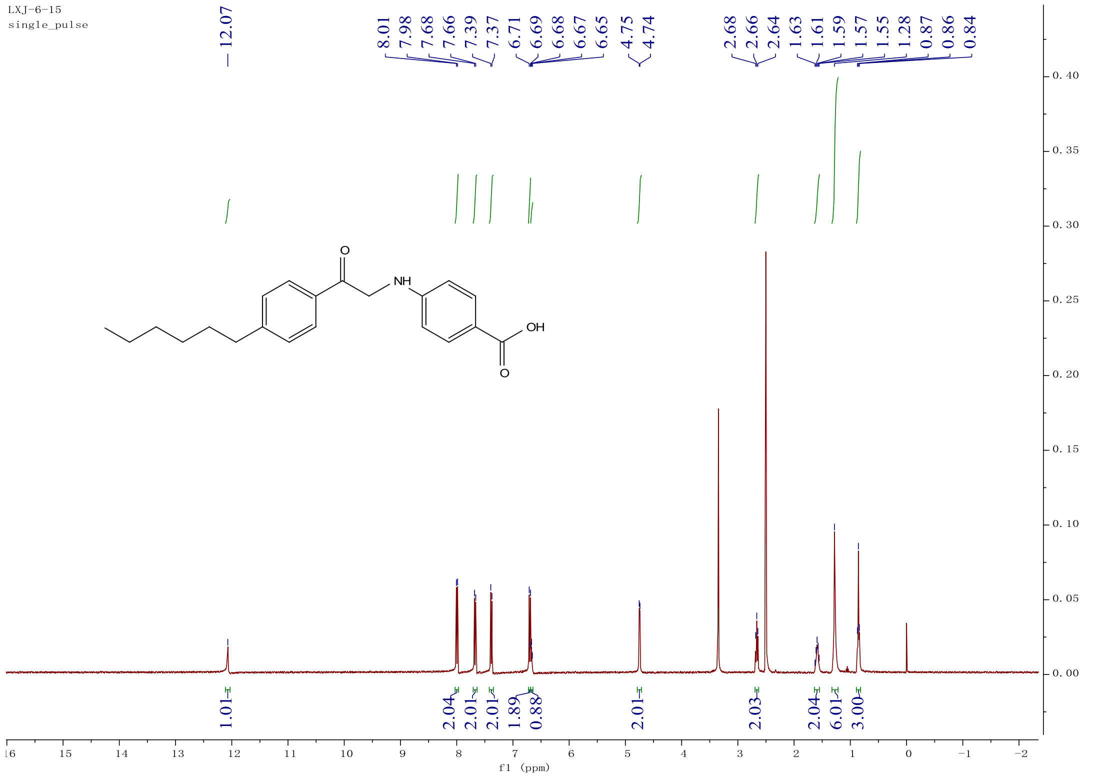

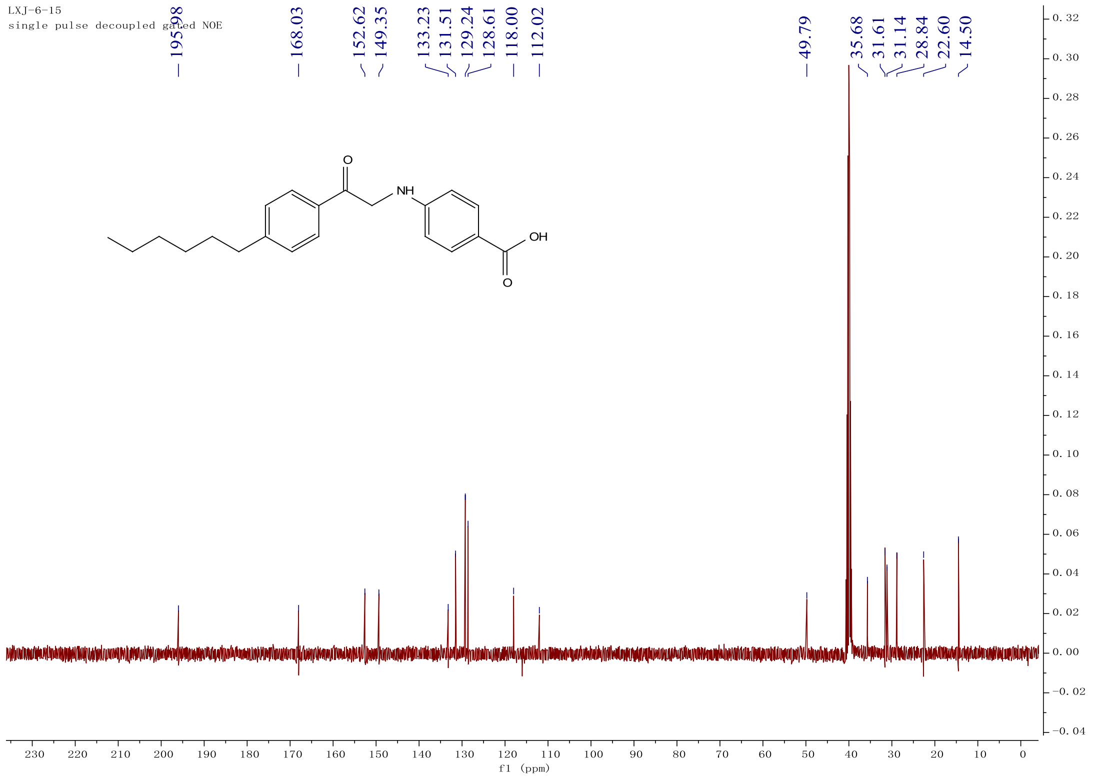

Fig S22. ^{1}H NMR and ^{13}C NMR spectra of **8d**.

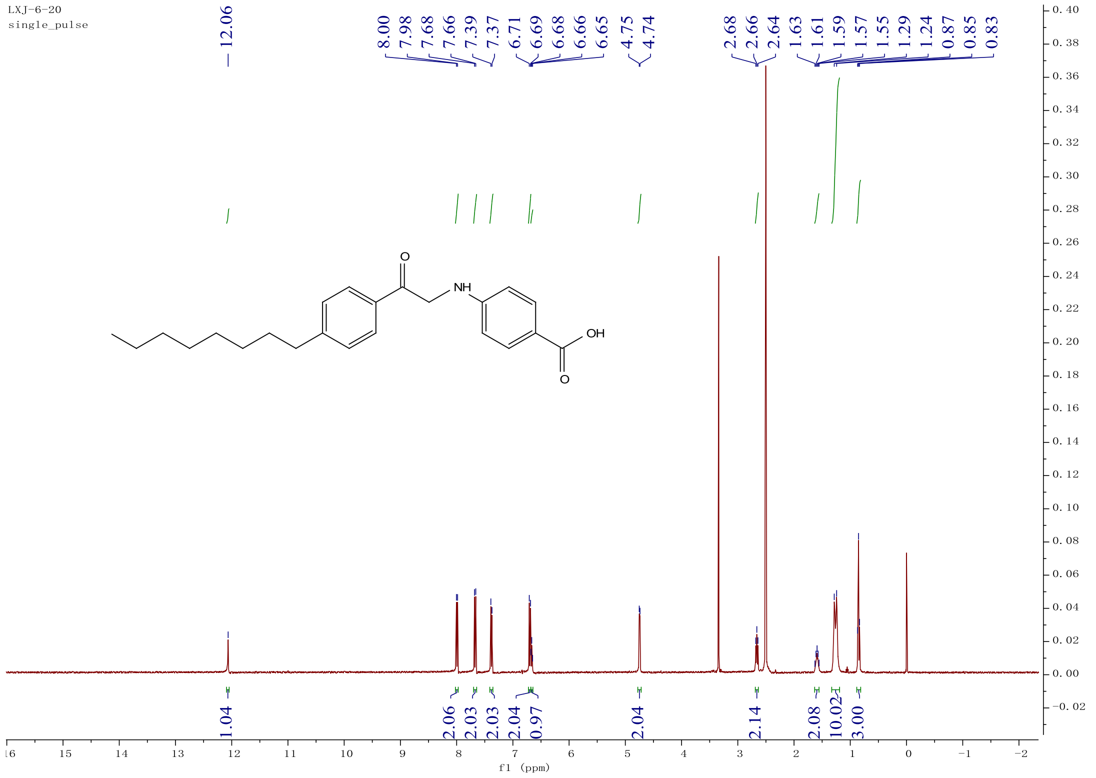

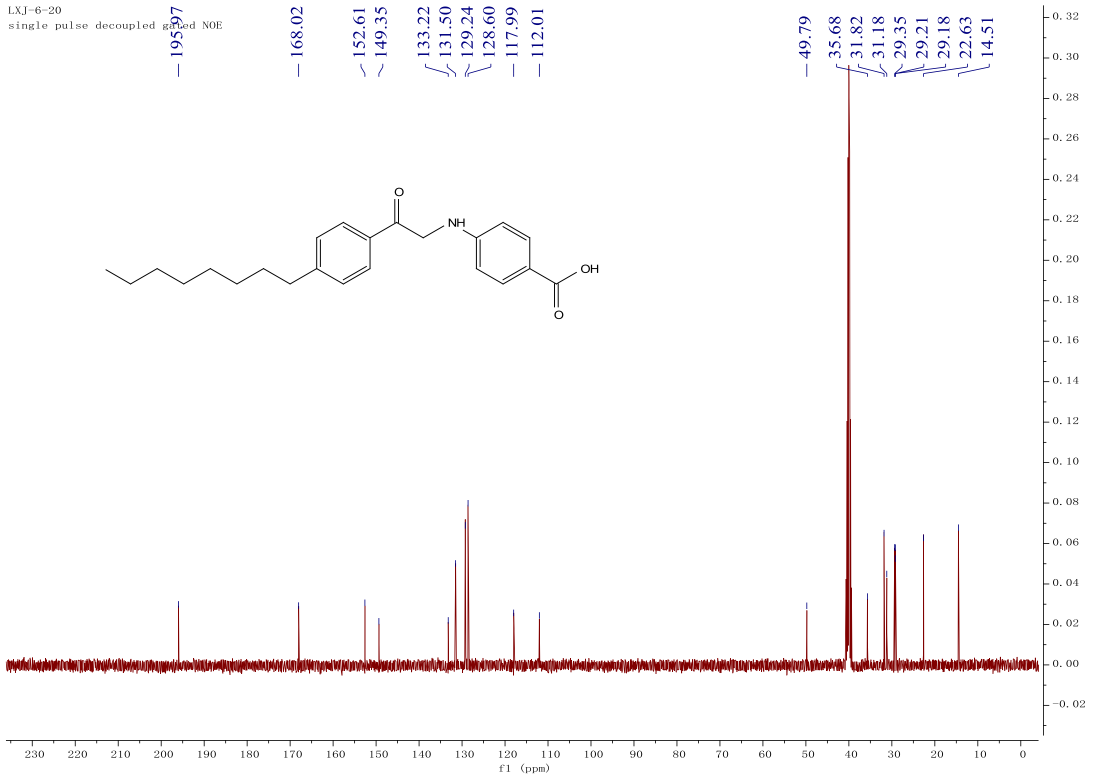

Fig S23. ^{1}H NMR and ^{13}C NMR spectra of **9a**.

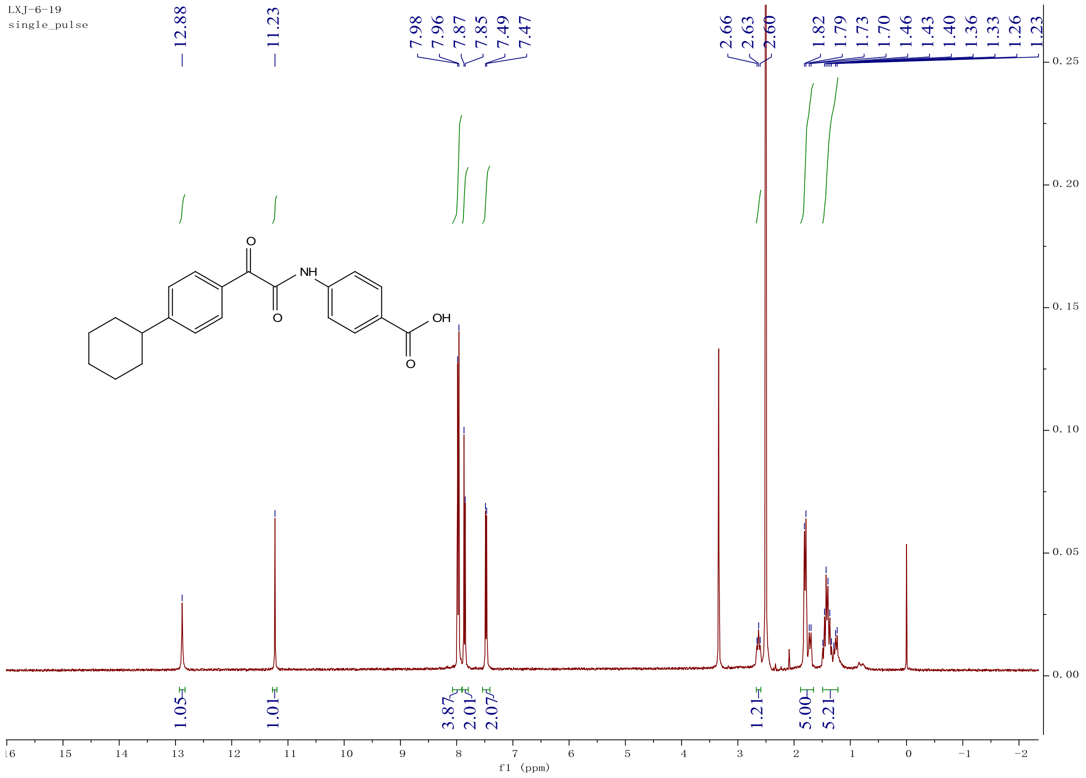

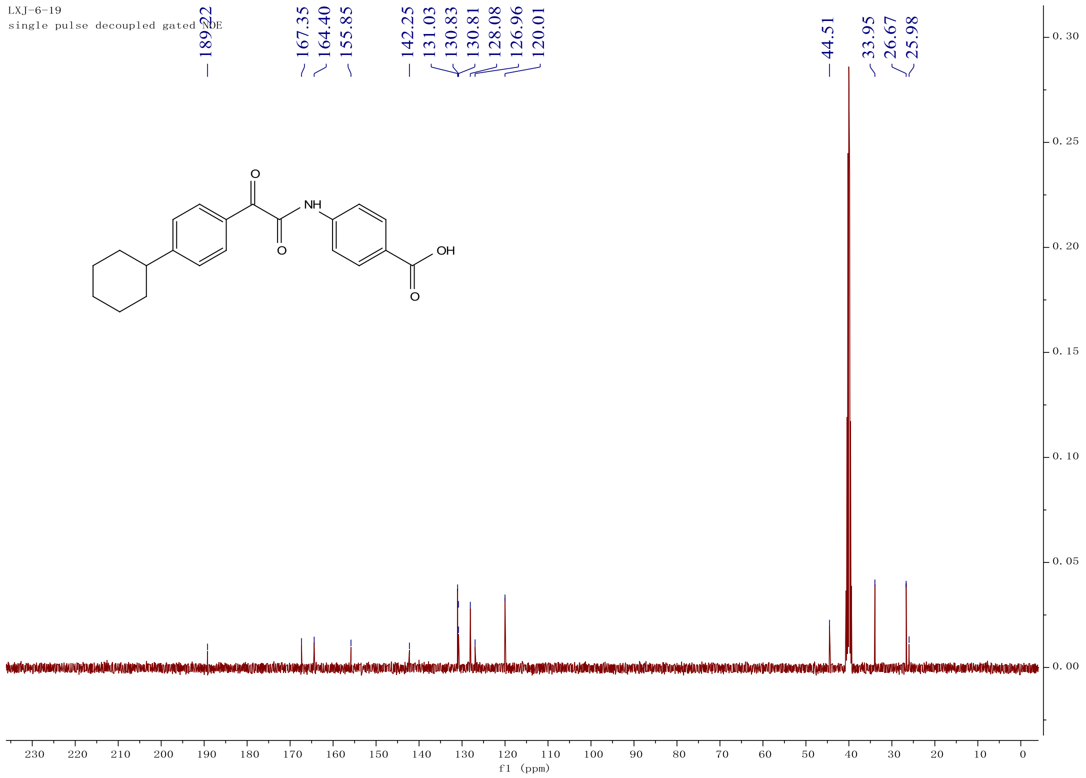

Fig S24. ^{1}H NMR and ^{13}C NMR spectra of **9b**.

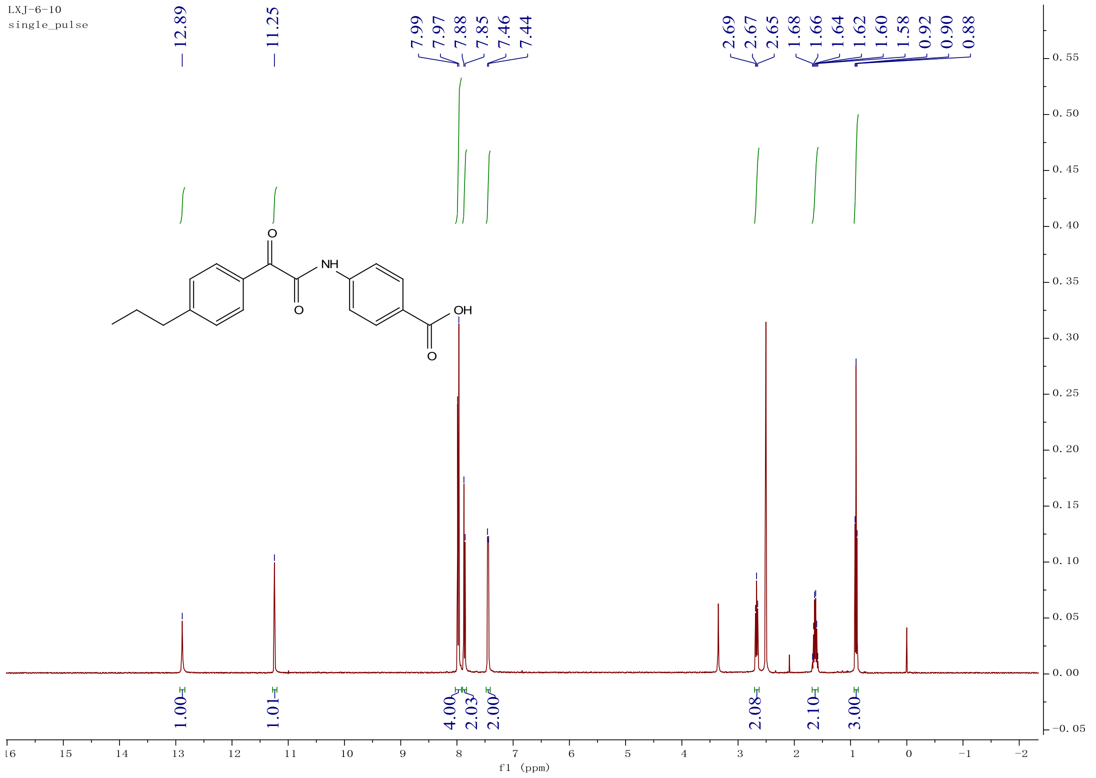

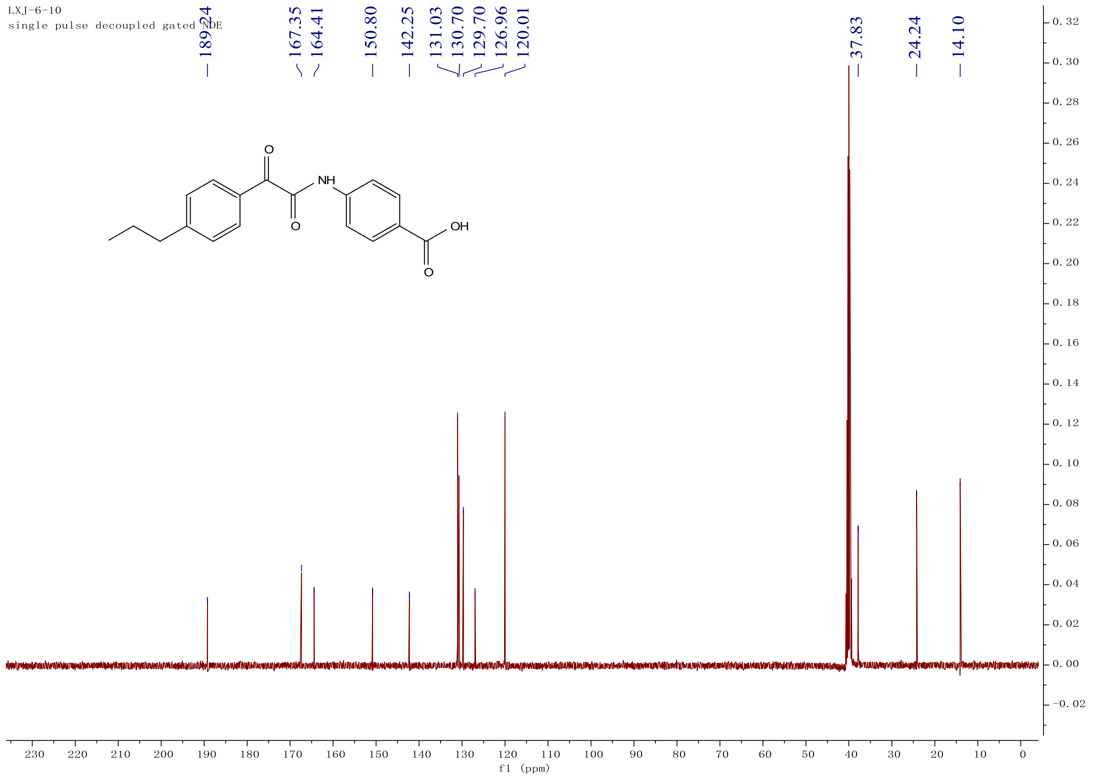

Fig S25. ^{1}H NMR and ^{13}C NMR spectra of **9c**.

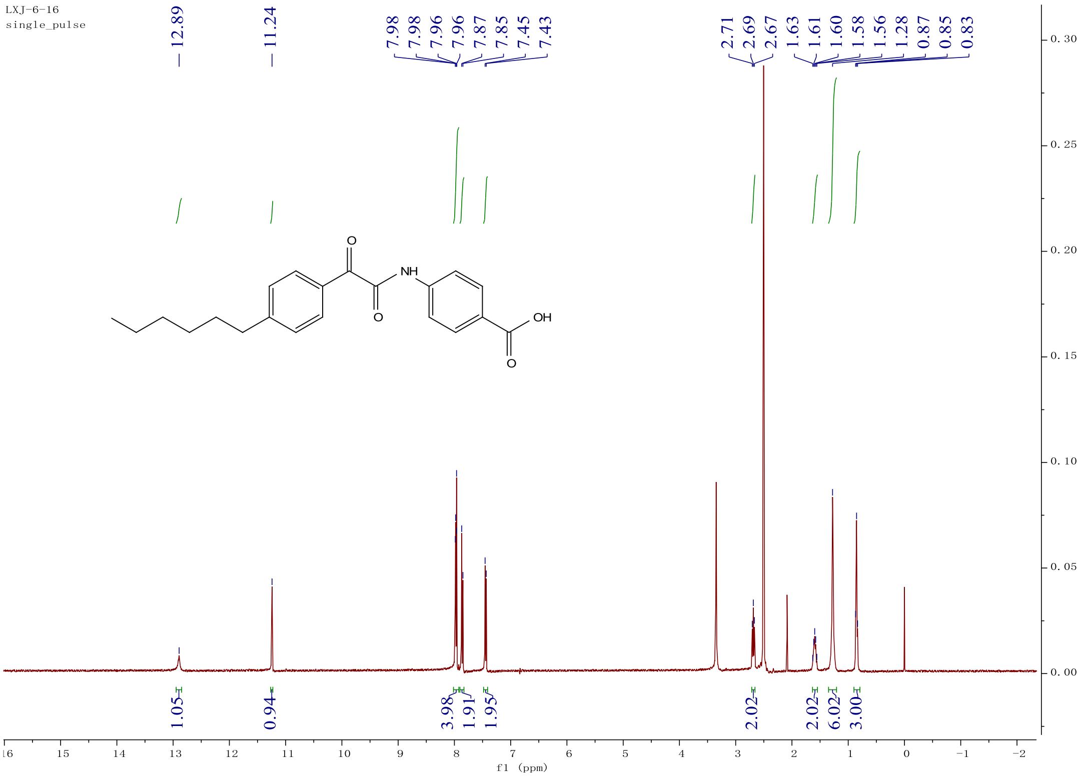

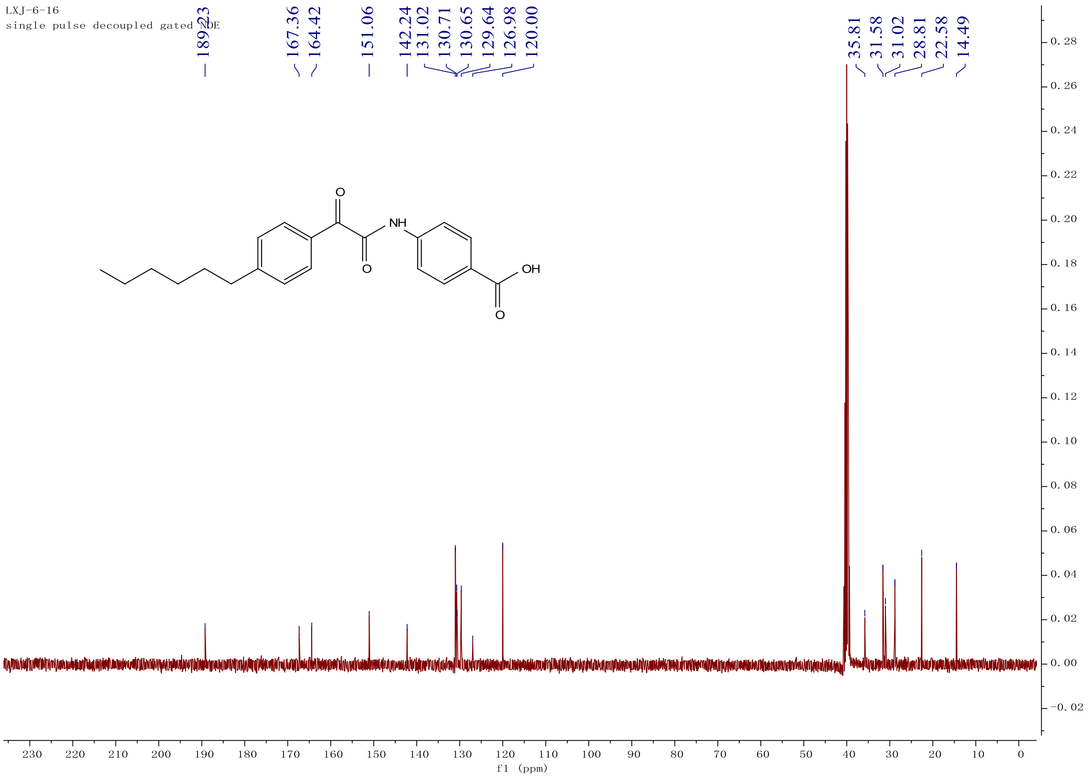

Fig S26. ^{1}H NMR and ^{13}C NMR spectra of **9d**.

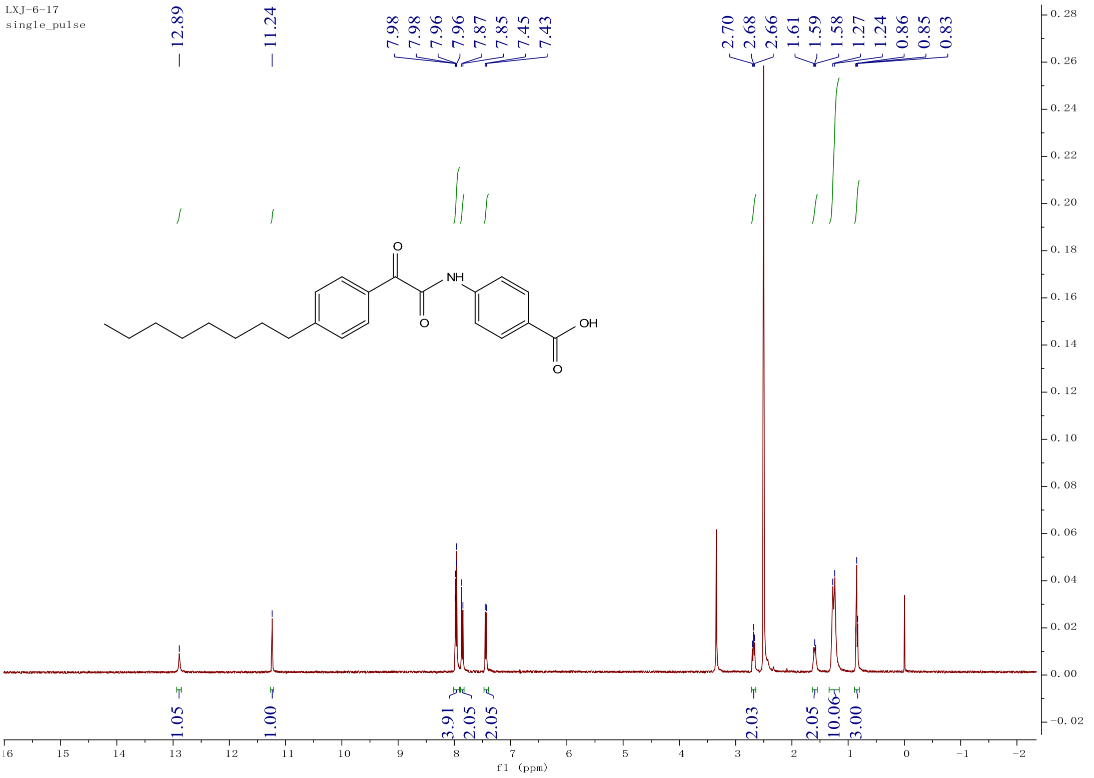

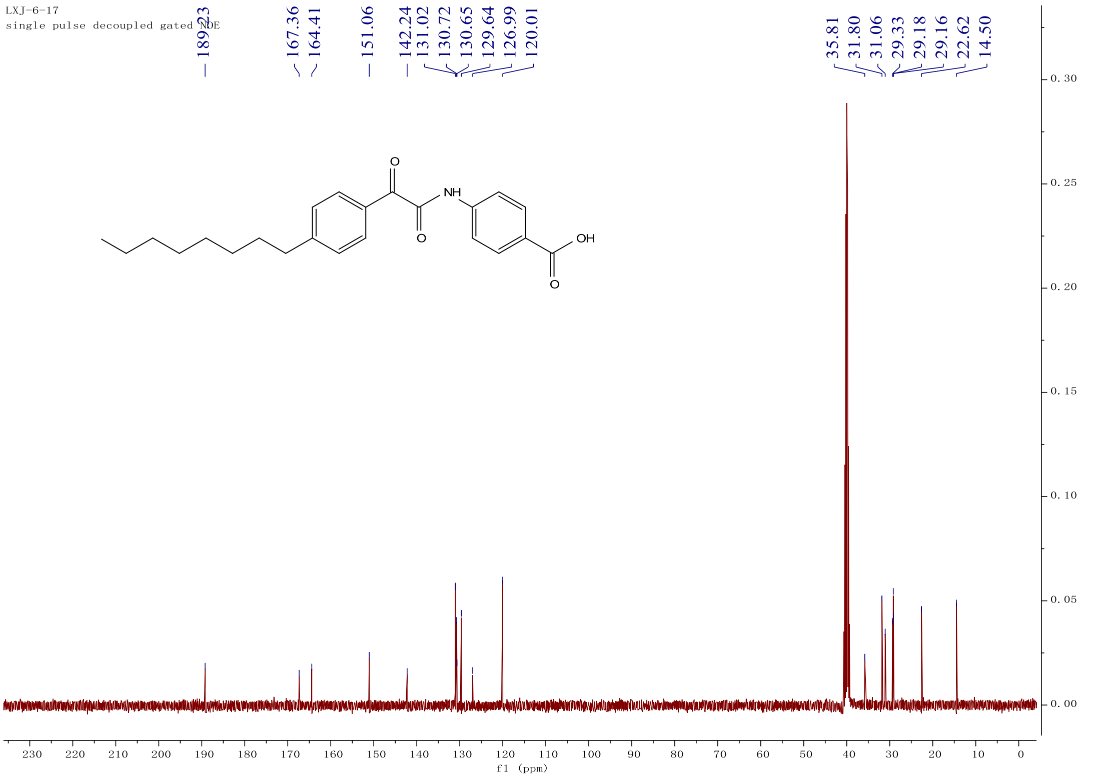

Fig S27. HRMS spectra of **6a.**

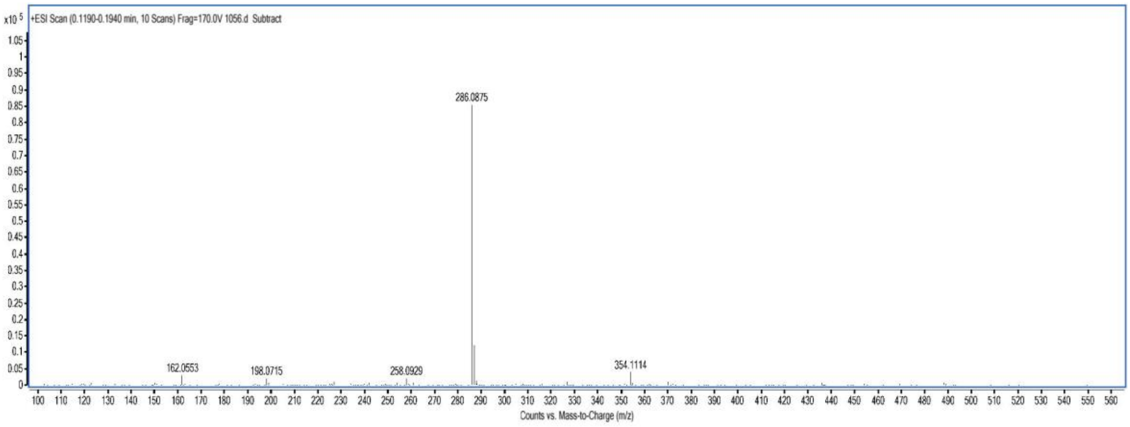

Fig S28. HRMS spectra of **6b.**

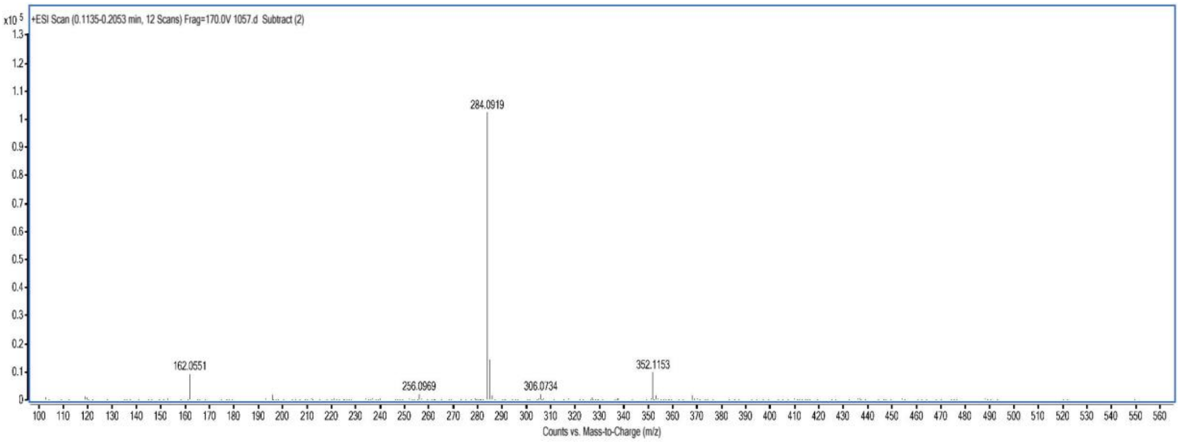

Fig S29. HRMS spectra of **6c.**

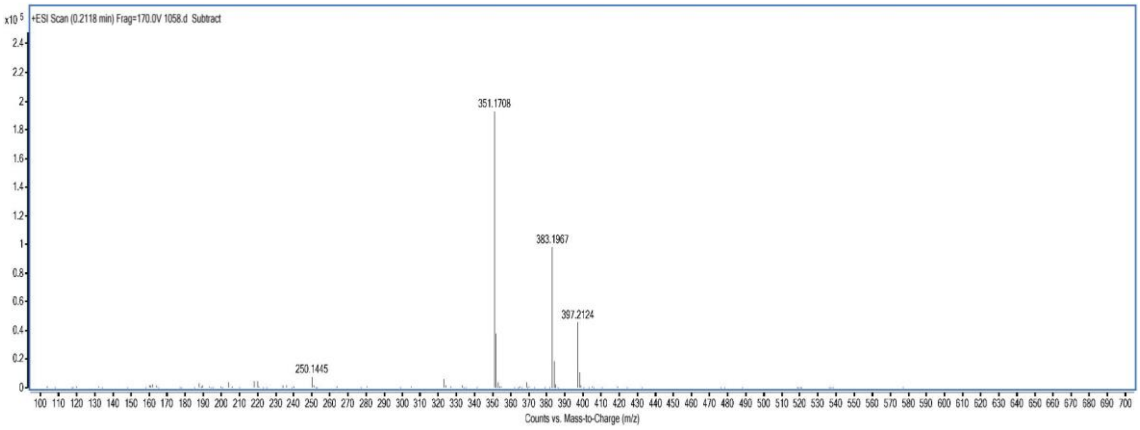

Fig S30. HRMS spectra of **6d.**

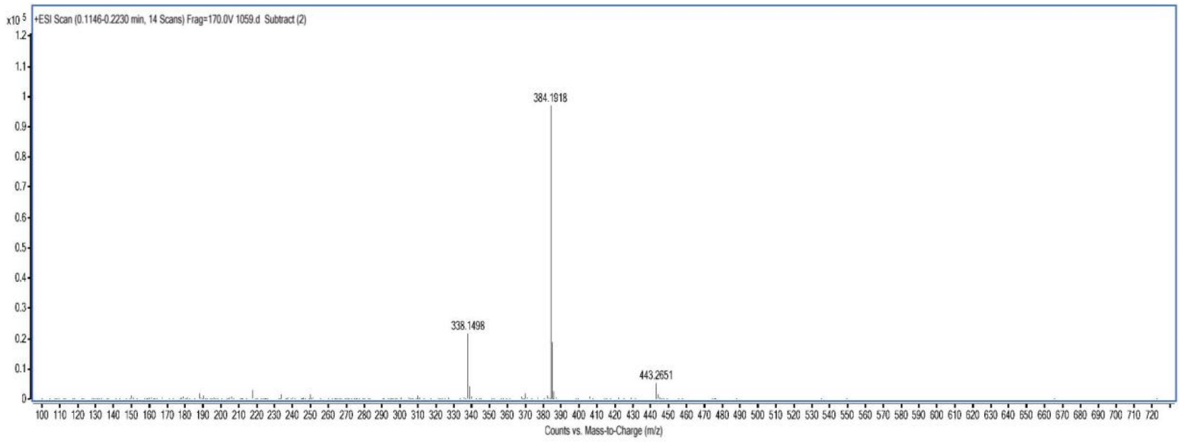

Fig S31. HRMS spectra of **6e.**

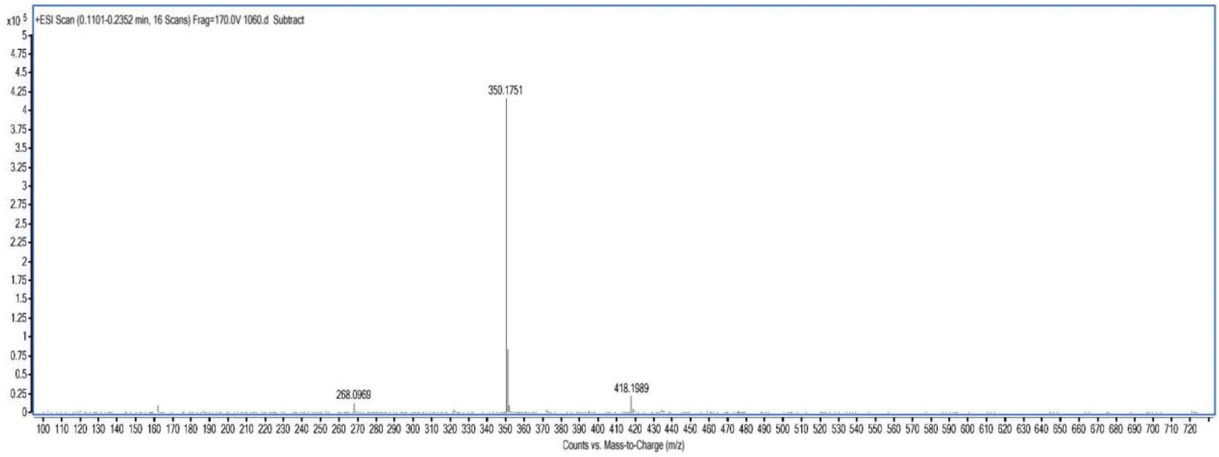

Fig S32. HRMS spectra of **6f.**

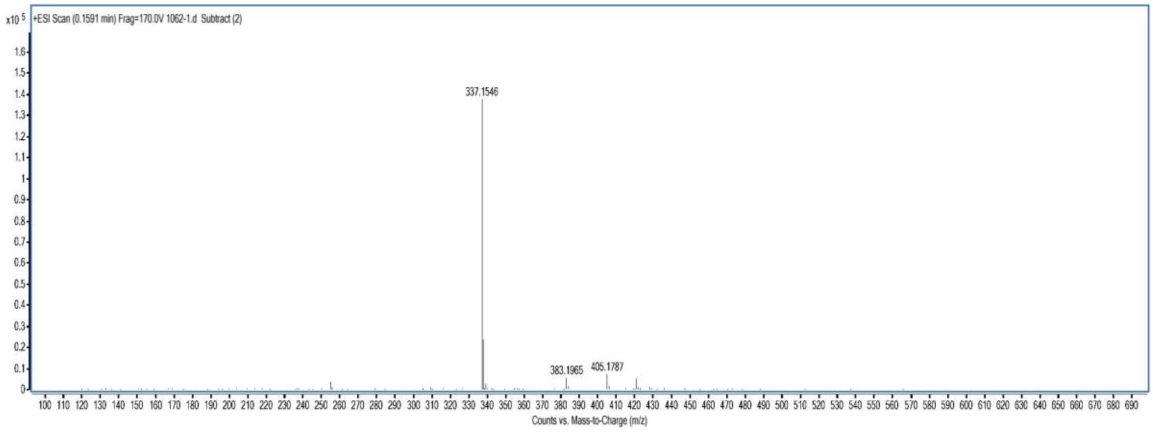

Fig S33. HRMS spectra of **6g.**

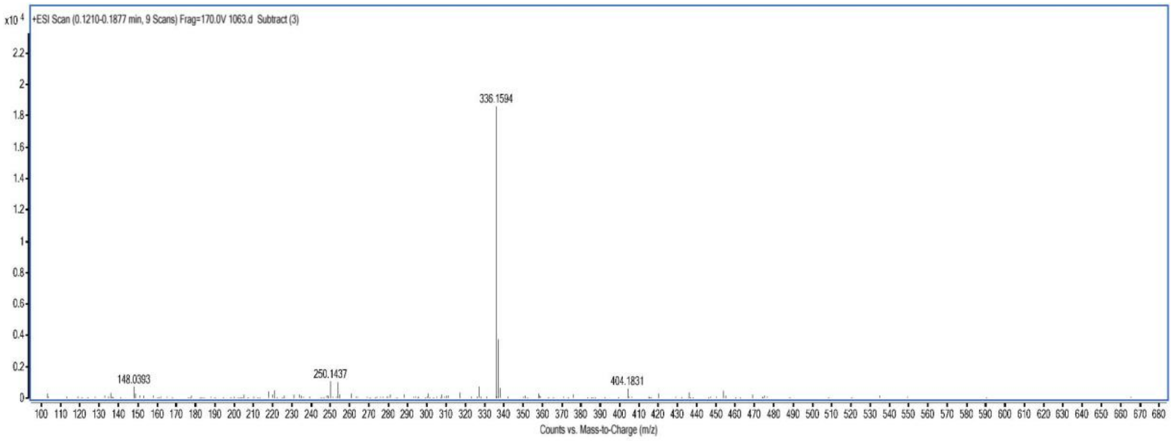

Fig S34. HRMS spectra of **6h.**

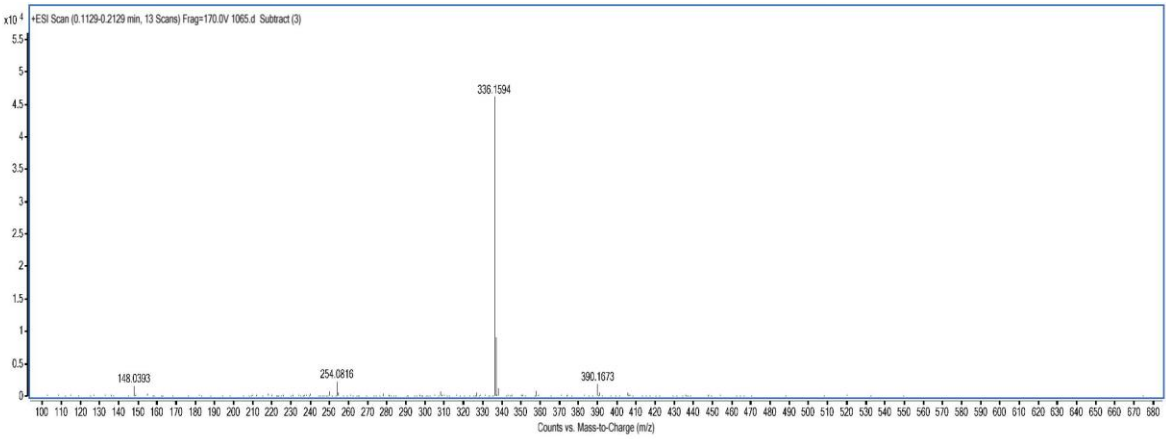

Fig S35. HRMS spectra of **6i.**

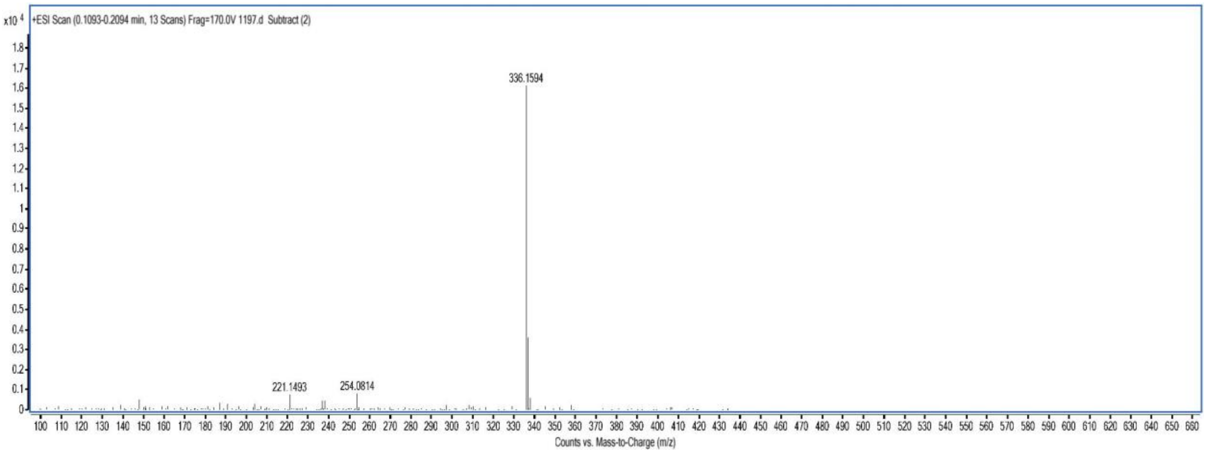

Fig S36. HRMS spectra of **6j.**

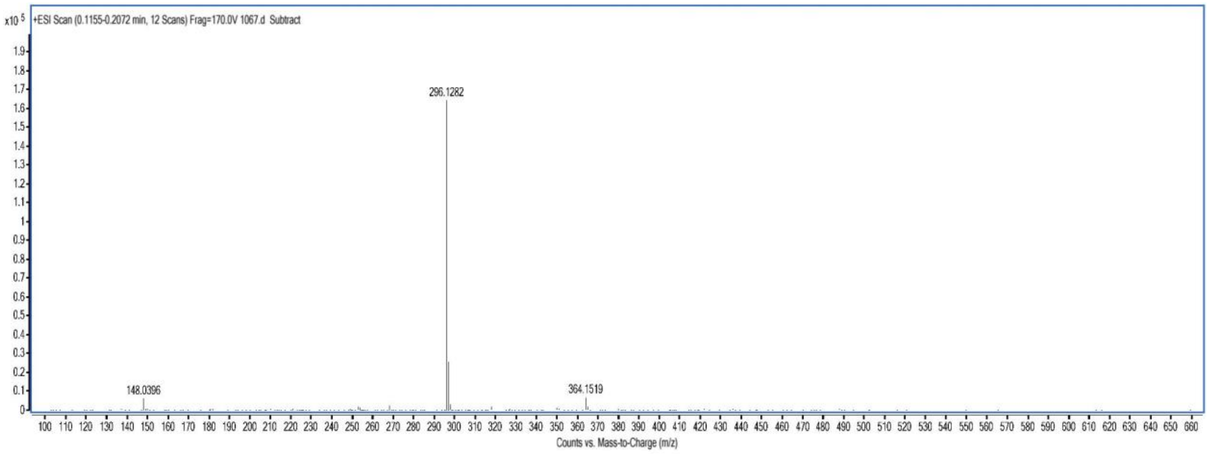

Fig S37. HRMS spectra of **6k.**

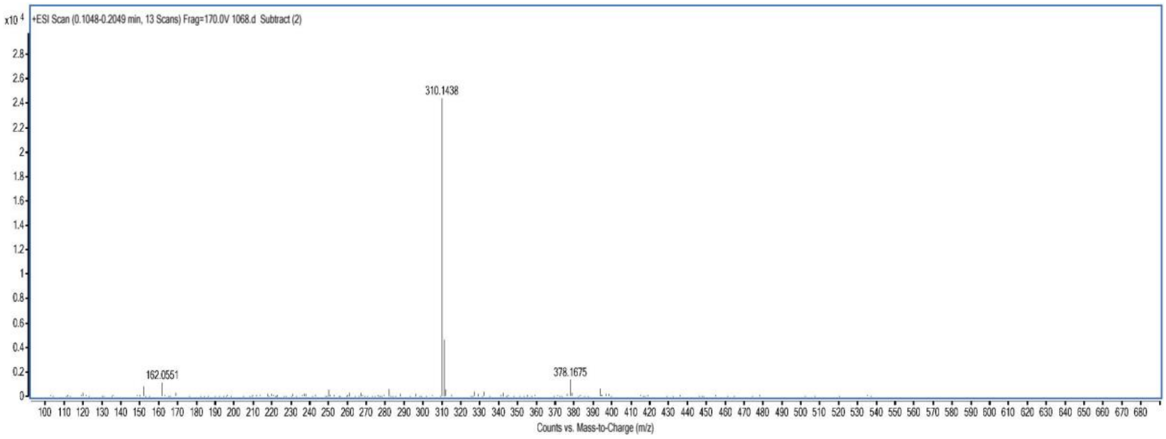

Fig S38. HRMS spectra of **6l.**

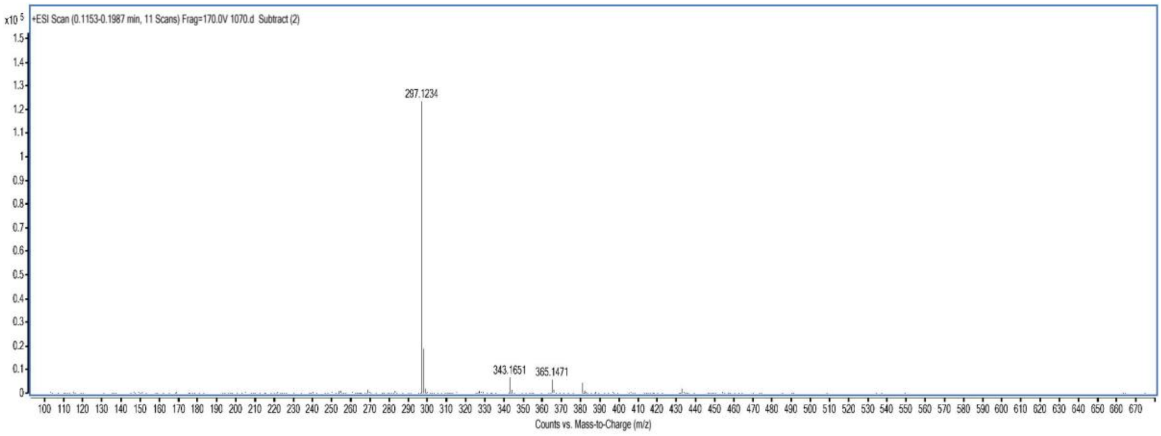

Fig S39. HRMS spectra of **6m.**

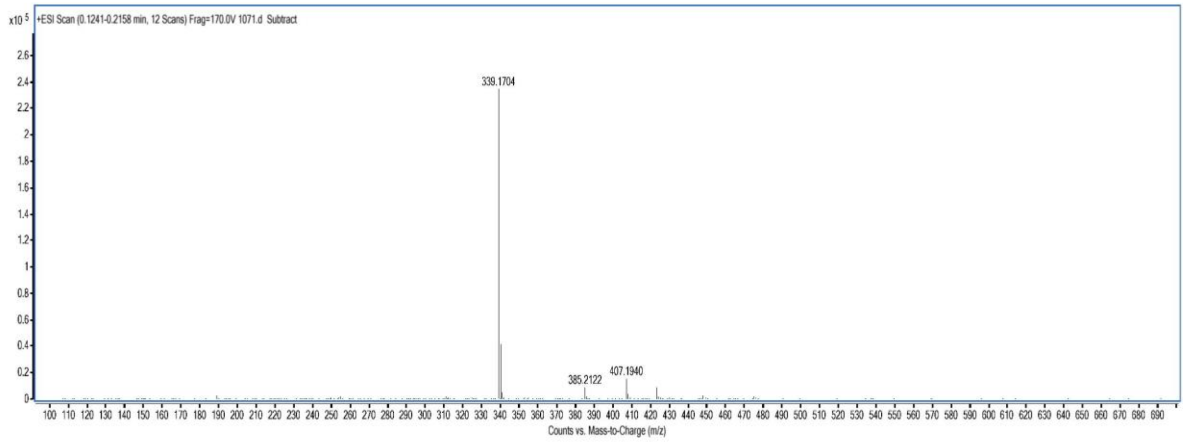

Fig S40. HRMS spectra of **6n.**

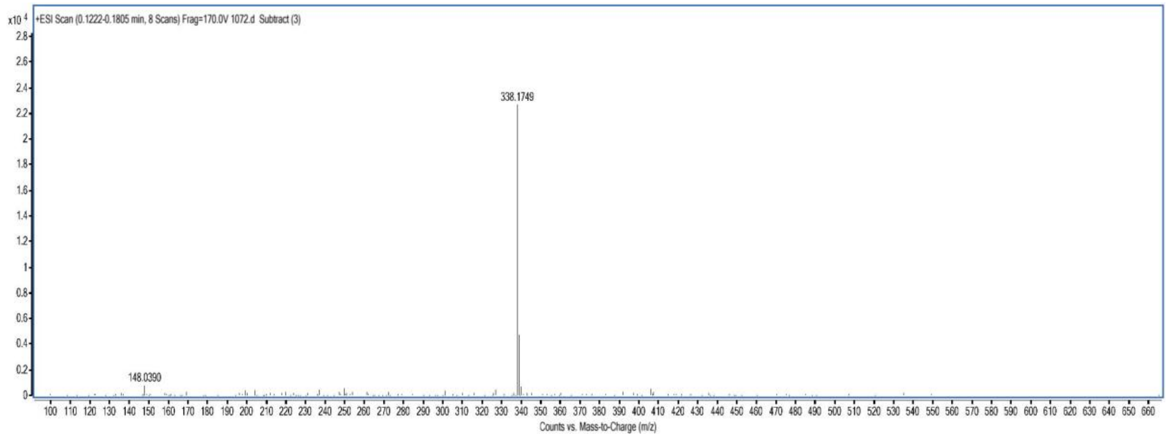

Fig S41. HRMS spectra of **6o.**

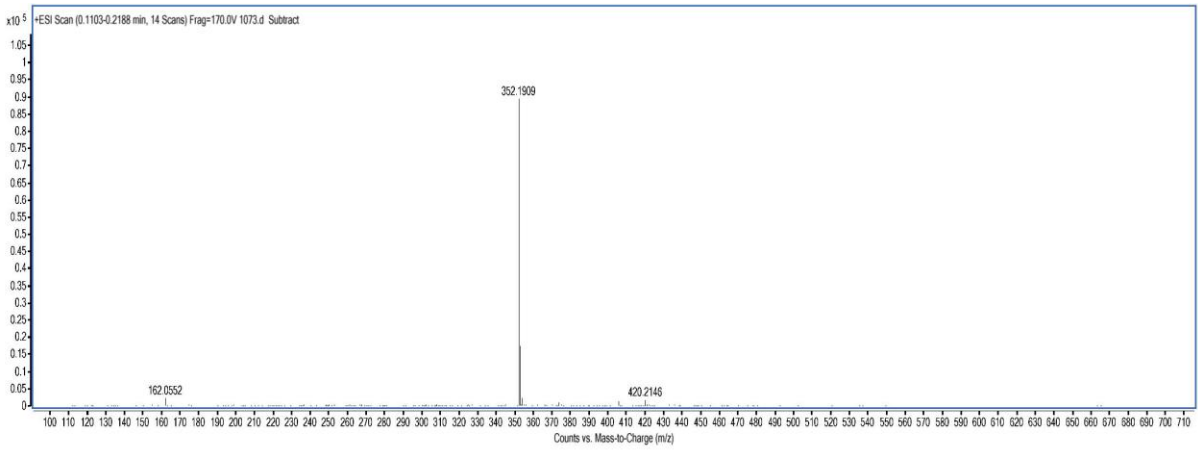

Fig S42. HRMS spectra of **6p.**

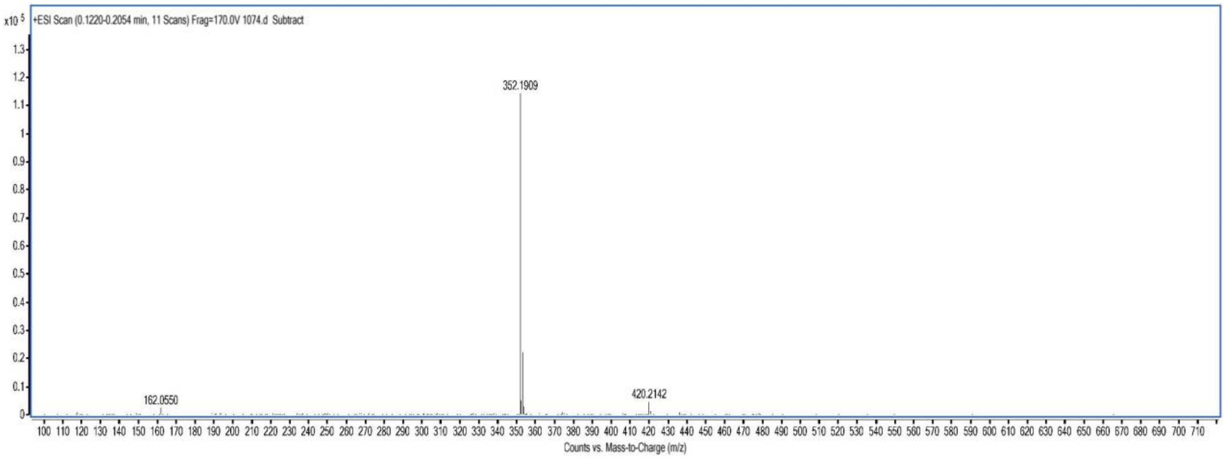

Fig S43. HRMS spectra of **6q.**

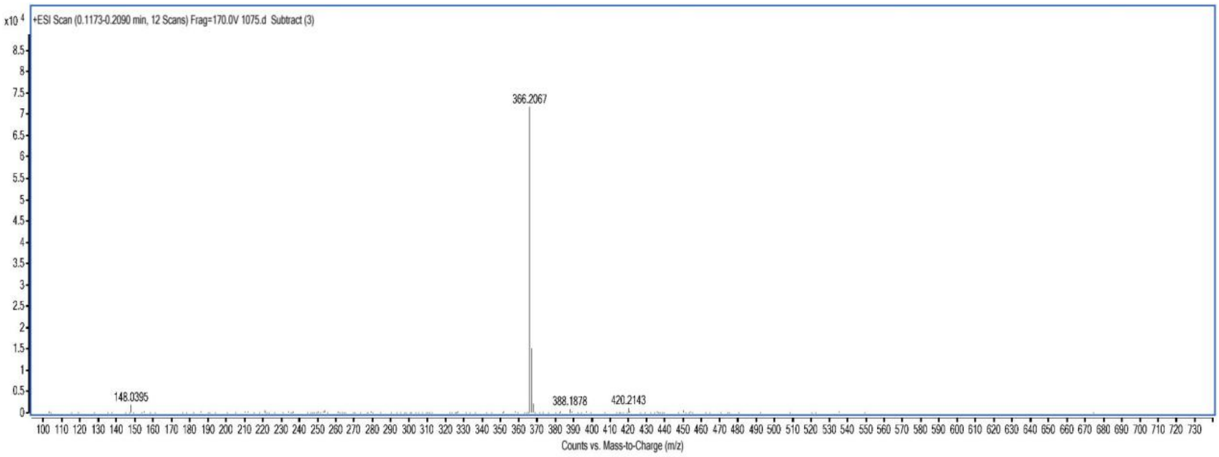

Fig S44. HRMS spectra of **6r.**

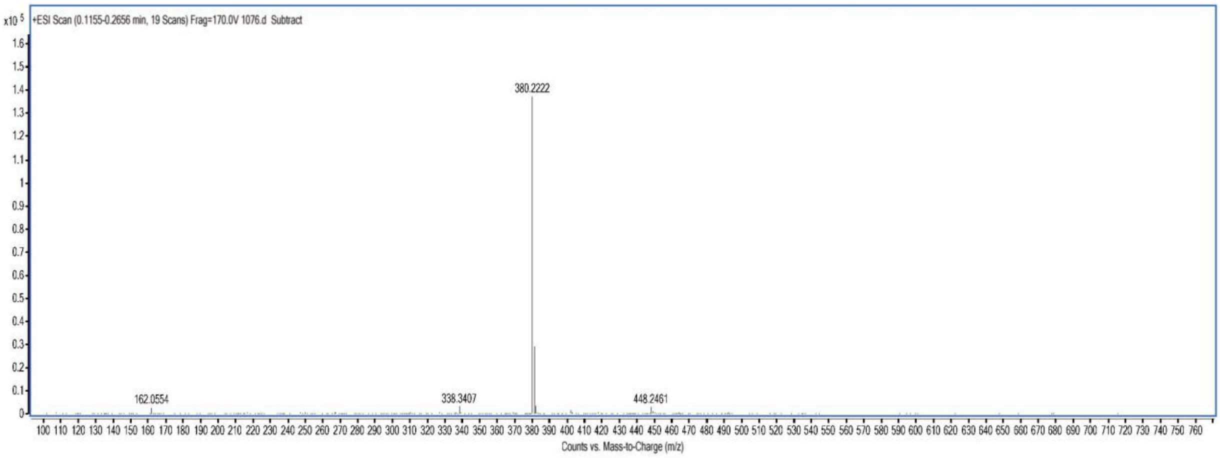

Fig S45. HRMS spectra of **8a.**

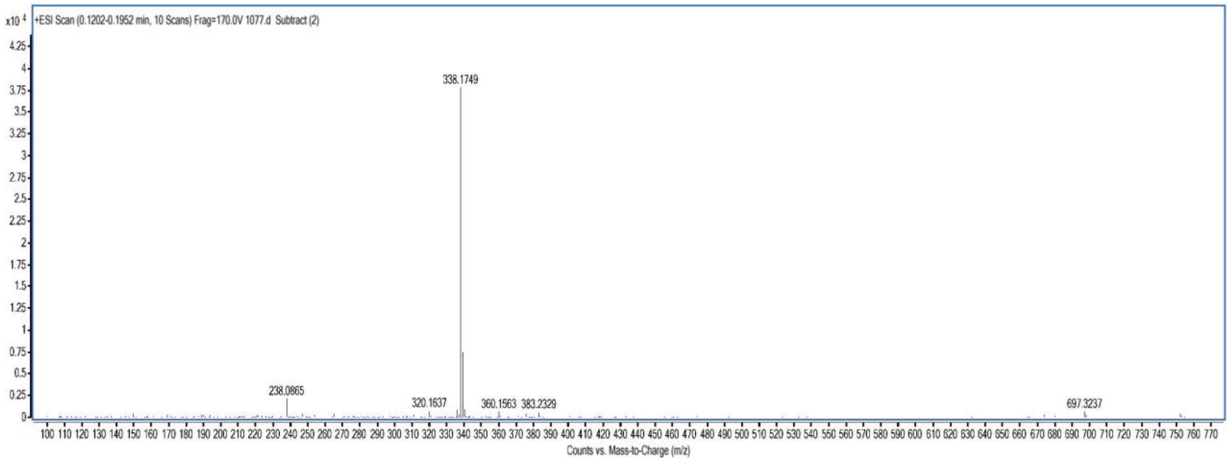

Fig S46. HRMS spectra of **8b.**

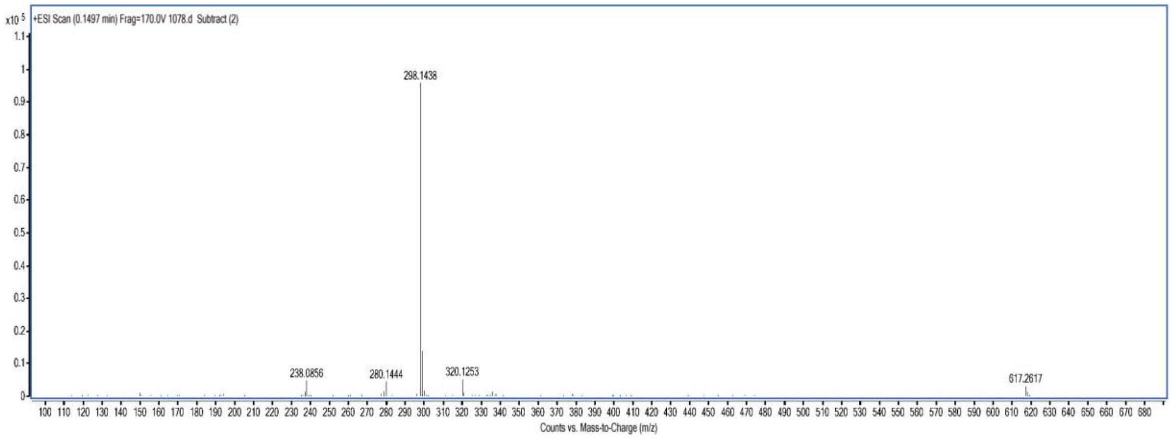

Fig S47. HRMS spectra of **8c.**

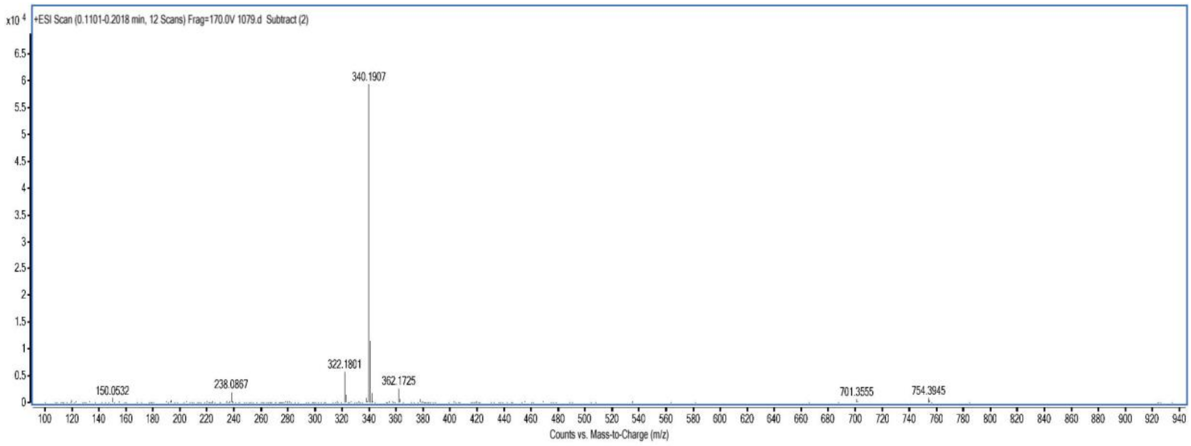

Fig S48. HRMS spectra of **8d.**

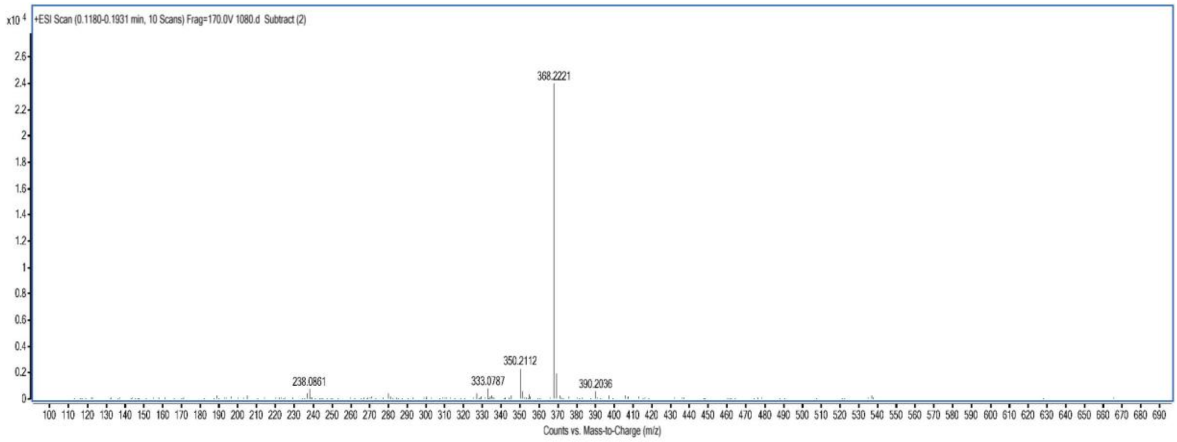

Fig S49. HRMS spectra of **9a.**

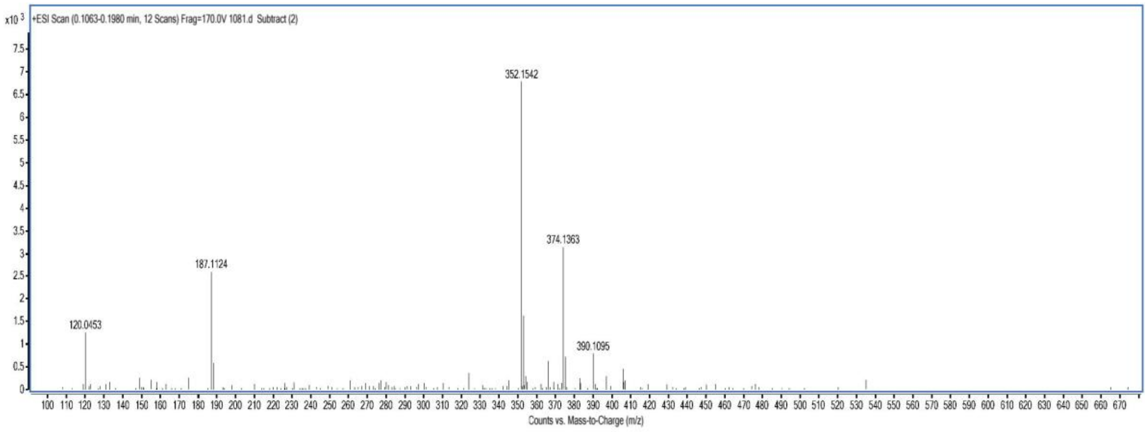

Fig S50. HRMS spectra of **9b.**

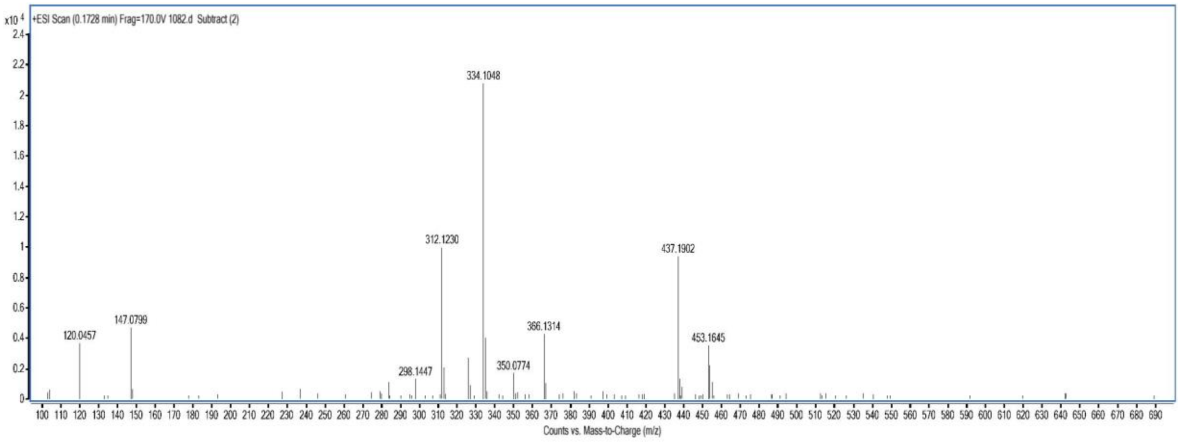

Fig S51. HRMS spectra of **9c.**

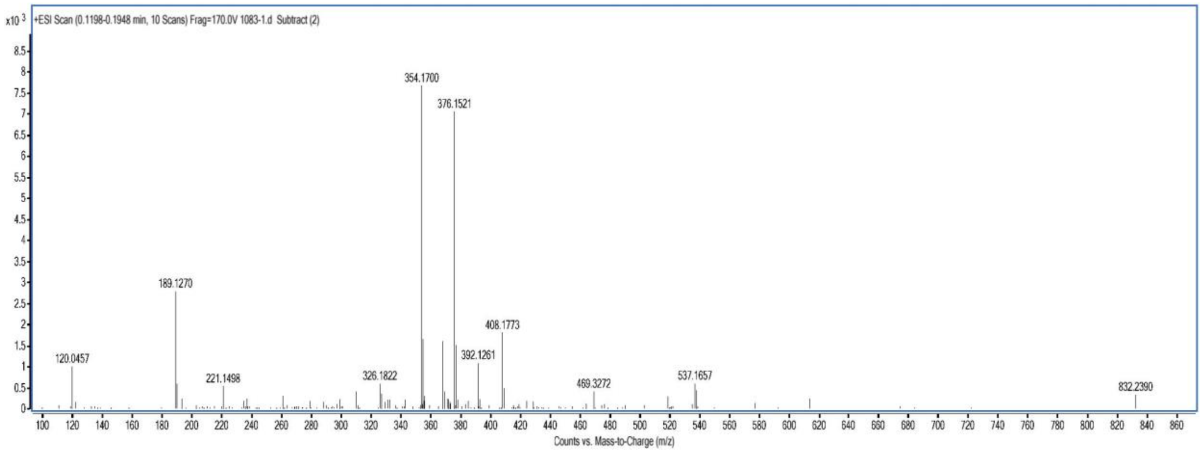

Fig S52. HRMS spectra of **9d.**

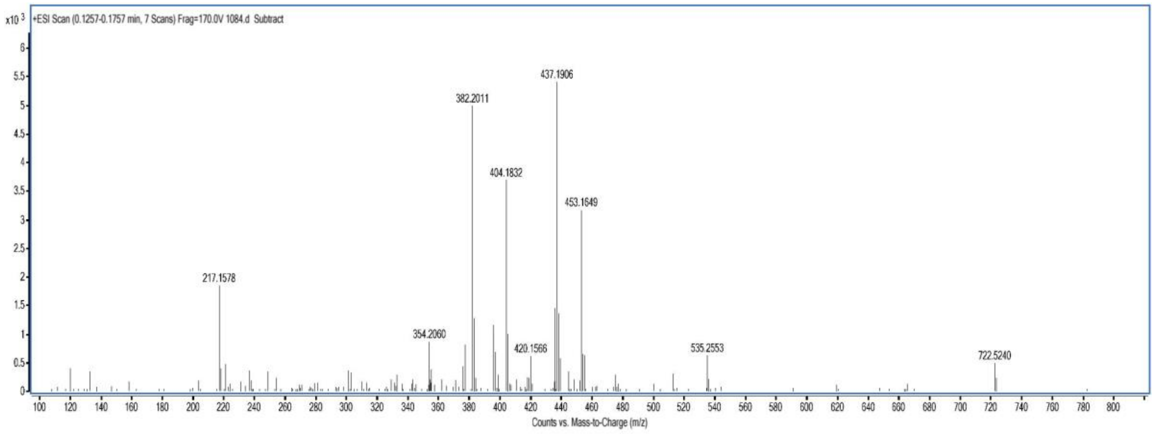

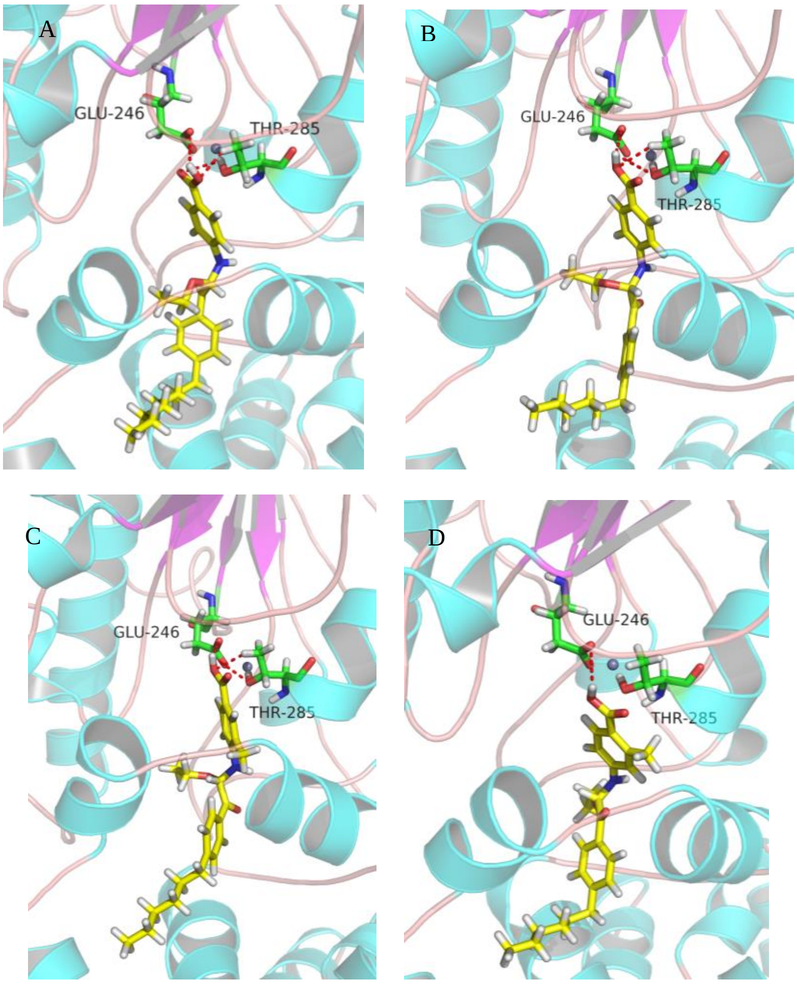

Fig.S53 Docking patterns of the interaction between compounds A: **6q**; B: **6n**; C: **6p**; D: **6o** and the full-length MCR-1 protein with H-bonds shown by red lines.
